# Therapy-induced senescence is a transient drug resistance mechanism in breast cancer

**DOI:** 10.1186/s12943-025-02310-0

**Published:** 2025-05-01

**Authors:** Eszter Bajtai, Csaba Kiss, Éva Bakos, Tamás Langó, Anna Lovrics, Éva Schád, Viktória Tisza, Károly Hegedűs, Péter Fürjes, Zoltán Szabó, Gábor E. Tusnády, Gergely Szakács, Ágnes Tantos, Sándor Spisák, József Tóvári, András Füredi

**Affiliations:** 1 Institute of Molecular Life Sciences, Center of Excellence of The Hungarian Academy of Sciences, HUN-REN Research Centre for Natural Sciences, Budapest, 1117 Hungary; 2 Semmelweis University Doctoral School, Budapest, 1085 Hungary; 3 Department of Experimental Pharmacology and the National Tumor Biology Laboratory, National Institute of Oncology, Budapest, 1122 Hungary; 4 National Laboratory for Drug Research and Development, Budapest, 1117 Hungary; 5 Doctoral School of Biology, Eötvös Loránd University, Budapest, 1117 Hungary; 6 Institute of Technical Physics and Materials Science, HUN-REN Centre of Energy Research, Budapest, 1121 Hungary; 7 Department of Medical Chemistry, Albert Szent-Györgyi Medical School, University of Szeged, Szeged, 6725 Hungary; 8 Department of Bioinformatics, Semmelweis University, Budapest, 1085 Hungary; 9 Center for Cancer Research, Medical University of Vienna, Vienna, 1090 Austria; 10 Physiological Controls Research Center, University Research and Innovation Center, Obuda University, Budapest, 1034 Hungary

**Keywords:** Therapy-induced senescence, Drug resistance, Breast cancer, Senescence escape, Tumor relapse

## Abstract

**Background:**

Therapy-induced senescence (TIS) is considered a permanent cell cycle arrest following DNA-damaging treatments; however, its irreversibility has recently been challenged. Here, we demonstrate that escape from TIS is universal across breast cancer cells. Moreover, TIS provides a reversible drug resistance mechanism that ensures the survival of the population, and could contribute to relapse.

**Methods:**

TIS was induced in four different breast cancer cell line with high-dose chemotherapy and cultured until cells escaped TIS. Parental, TIS and repopulating cells were analyzed by bulk and single-cell RNA sequencing and surface proteomics. A genetically engineered mouse model of triple-negative breast cancer was used to prove why current senolytics cannot overcome TIS in tumors.

**Results:**

Screening the toxicity of a diverse panel of FDA-approved anticancer drugs revealed that TIS meditates resistance to half of these compounds, despite their distinct mechanism of action. Bulk and single-cell RNA sequencing, along with surface proteome analysis, showed that while parental and repopulating cells are almost identical, TIS cells are significantly different from both, highlighting their transient nature. Furthermore, investigating dozens of known drug resistance mechanisms offered no explanation for this unique drug resistance pattern. Additionally, TIS cells expressed a gene set associated with immune evasion and a potential KRAS-driven escape mechanism from TIS.

**Conclusion:**

Our results reveal that TIS, as a transient drug resistance mechanism, could contribute to overcome the immune response and to relapse by reverting to a proliferative stage.

**Supplementary Information:**

The online version contains supplementary material available at 10.1186/s12943-025-02310-0.

## Background

Cancer claims 10 million lives annually, largely due to the emergence of drug resistance [1]. Drug resistant cancer cells employ various strategies, such as inactivating [2] or effluxing drug molecules [3], enhancing DNA repair to counteract DNA damage [4], evading apoptosis by distorting apoptotic pathways [5], reducing target proteins expression [6, 7] or temporarily arresting the cell cycle to avoid drugs that target rapidly dividing cells [8]. Recent discoveries have further expanded the repertoire of resistance mechanisms: disruption in the tumor microenvironment can promote drug resistance [9] and treatment induced epigenetic changes give rise to drug-tolerant persister cells [10]. Tumor heterogeneity, where different cell populations within the tumor exhibit diverse molecular characteristics and drug sensitivities, also promotes resistance [11] as does the ability to evade immunosurveillance [12]. However, despite these discoveries, attempts to translate these findings into clinical practice have largely failed, leading to the sobering realization that the contribution of each individual mechanism to overall drug resistance may be more limited than previously thought.

When cellular damage occurs, the subsequent response is contingent upon the severity of the injury. In cases of mild damage, cells may activate repair mechanisms to restore homeostasis. However, if the damage exceeds the cell's reparative capacity, programmed cell death pathways, such as apoptosis, or uncontrolled cell death via necrosis, become inevitable outcomes [13]. However, in the narrow range between reversible and irreversible damage there exists a third option, called cellular senescence. Senescence was first described by Hayflick as a response to cellular aging. They observed that fetal fibroblasts could undergo only a limited amount of cell divisions before irreversibly losing their ability to replicate, a state termed replicative senescence [14]. It was discovered that telomere shortening, caused by continuous cell division, triggers senescence in cells, which is why cancer cells frequently overexpress telomerase to avoid senescence [15–19]. In addition to aging, cellular senescence can be induced by various stress factors such as DNA damage, abnormal cell replication or oxidative stress [20]. Senescence serves as a crucial protective mechanism for damaged cells; while these cells lose their ability to replicate, they can still contribute to tissue homeostasis through processes like remodeling, repair, and regeneration in certain contexts [21].

Senescence is an enigmatic stress response mainly due to its many interconnected subtypes [21]. Replicative senescence, DNA-damage induced senescence, oncogene-induced senescence (OIS), oxidative stress-induced senescence, therapy-induced senescence (TIS) and epigenetically induced senescence all lead to similar outcomes, yet the molecular pathways driving these changes can vary significantly. Despite these differences, the irreversibility of senescence is so widely accepted that it has become a desired outcome in cancer therapy. If treatment cannot eliminate every tumor cell, it can still inflict enough damage to induce permanent cell cycle arrest, which effectively achieves the same goal [22, 23]. While senescence is widely regarded as a permanent state, emerging studies suggest that cancer cells can escape senescence more frequently than previously thought. For example, lung cancer cells lacking p53 and p16 can re-enter cell cycle after TIS by overexpressing Cdc2/Cdk1 affecting approximately 1 in a million cells [24]. Additionally, in locally advanced non-small cell lung cancer, cells that escape TIS are often polyploid, a transient state in which senescence escape is more likely to occur [25]. In B-cell lymphoma and lymphoblastic leukemia cells released from TIS by inducible alterations in H3 K9 trimethylation or p53 expression exhibited increased stemness, enhanced Wnt-signaling and leukemia-initiating properties [26]. Furthermore, lung, colon, and breast cancer cells have been shown to escape TIS in vitro and form tumors in vivo when injected into mice [27].

Here, we demonstrate that chemotherapy induces senescence in four distinct breast cancer cell lines, followed by a delayed escape from the TIS state, with cells re-entering the cell cycle weeks later. Remarkably, during TIS, cancer cells exhibit resistance to a wide range of drugs with diverse structures and mechanisms of action. Through bulk and single-cell RNA sequencing (scRNA-seq), we identify a novel TIS transcriptome shared across all cell lines, revealing that TIS cells are fundamentally distinct from both control (CTR) and repopulated (REPOP) cells. We propose that TIS is not only a reversible state but also a critical mechanism of drug resistance, with profound implications for cancer treatment and therapy design.

## Methods

### Cell lines

The human mammary carcinoma cells lines (MCF7, T47D, MDA-MB-231, Hs578T) were obtained from the National Cancer Institute’s Developmental Therapeutics Program (National Institutes of Health, Bethesda, MD, USA). Cells were cultured in Roswell Park Memorial Institute (RPMI) medium (Thermo Fisher Scientific, Massachusetts, USA), supplemented with 10% fetal bovine serum (FBS, Thermo Fisher Scientific, Massachusetts, USA), 5 mmol/L glutamine (Euroclone, Pero MI, Italy), and 50 units/mL penicillin and streptomycin (Capricorn Scientific, Ebsdorfergrund, Germany). All cell lines were cultured at 37°C with 5% CO_2_.

Human foreskin fibroblast (HFF) cell line was obtained from the National Cancer Institute’s Developmental Therapeutics Program. Cells were cultured in Dulbecco’s modified Eagle’s medium (DMEM-F12, Thermo Fisher Scientific, Massachusetts, USA) supplemented with 10% fetal bovine serum (FBS, Thermo Fisher Scientific, Massachusetts, USA), 1% L-glutamine (Thermo Fisher Scientific, Massachusetts, USA), 0.1% gentamicin (50 mg/mL, Thermo Fisher Scientific, Massachusetts, USA), and 16 ng/mL fibroblast growth factor 2 (Peprotech, London, UK). Cells were kept at 37°C and 5% CO_2_.

### Drugs

The complete list of drugs can be seen in the Supplemental Information.

### TIS induction

For TIS induction cells were treated with 120 nM (MCF7), 70 nM (T47D), 150 nM (MDA-MB-231), 200 nM (Hs578T), 210 nM (HFF) DOX and incubated for 5 days. After five days of incubation, treated cells were washed with pre-warmed PBS and the medium was renewed. MCF7, T47D, MDA-MB-231 and Hs578T plates remained in the incubator for seven days to ensure that the cells had become senescent. HFF plates remained in the incubator for 19 days with medium change every week. REPOP cells are those that survived DOX treatment, underwent TIS, expressed all canonical senescence markers, but later re-entered the cell cycle and lost TIS-associated attributes. These cells were analyzed when they reached ~ 80% confluency. re-TIS cells were established by replating REPOP cells and re-exposing them to DOX to induce TIS again. Visual representation of TIS induction and different cell states are summarized on Figure S1.

### SA-β-Gal staining

Cells were plated into 96-well tissue culture plates. SA-β-Gal staining was performed using the Senescence β-Galactosidase Staining Kit (Cell Signaling Technology, Danvers, MA, USA) according to the manufacturer’s instructions.

### In vitro cytotoxicity assay

10.000 cells/well were plated in 96-well plates. TIS induction was performed as described above. After TIS induction cells were counted in three wells and the results were averaged, after which the appropriate number of CTR cells was plated. The following day, both TIS and CTR cells were treated with a given concentration range of different drugs and incubated for five days. Drug testing on re-TIS cells was conducted under comparable conditions. A total of 10.000 REPOP cells per well were seeded onto 96-well plates, and TIS was induced by treatment with DOX over a period of five days. Following this incubation, the treated cells were washed with pre-warmed PBS, and the culture medium was replenished. The cells were then maintained in culture for an additional seven days. On Day 12, the cells were exposed to the selected compounds for further analysis. Viability was assessed using the PrestoBlue® assay (Thermo Fisher Scientific, Massachusetts, USA), according to the manufacturer’s instructions. Cells were incubated for 1.5 h with 5% PrestoBlue® Cell Viability Reagent diluted in PBS at 37°C/5% CO2. The fluorescence signal was measured spectrophotometrically using an EnSpire microplate reader (Perkin Elmer, Waltham, Massachusetts, USA). Data were normalized to untreated cells, curves were fitted by the GraphPad Prism version 8.0.1 for Windows (GraphPad Software, Boston, Massachusetts USA) using the sigmoidal dose–response model.

### Immunocytochemistry and fluorescent staining

Cells were seeded into glass-bottomed 8-well slides (ibidi, Gräfelfing, Germany) at 20.000 cells/well density. TIS induction was performed as described above. CTR cells were seeded at 20.000 cells/well density.

#### Immunocytochemistry

TIS and CTR cells were fixed with 4% PFA for 15 min at room temperature and washed twice with PBS. Complete blocking solution (0.5% BSA-PBS, 0.1% Triton X-100, 5% goat serum, and 1% fish gelatin) was used for one hour at room temperature. Samples were incubated overnight at 4°C with γ-H2A.X, Bcl-2 and Bcl-XL (Thermo Fisher Scientific, Waltham, MA, USA) primary antibodies. After incubation, the cells were washed with PBS, and the secondary antibody Alexa Fluor 488 (Thermo Fisher Scientific, Waltham, MA, USA) was added in complete blocking solution. Nuclei were labeled with DAPI (Dojindo EU, Munich, Germany). Cells were observed with a Zeiss LSM-710 confocal microscopy at 40 × magnification.

#### Fluorescent staining

Fluorescent staining with Nucleolus Bright Red, Cellular Senescence Detection Kit-SPiDER-βGal, MitoBright Red, LysoTracker Red (Dojindo EU, Munich, Germany) was performed according to the manufacturer’s instructions. Cells were observed with a Zeiss LSM-710 confocal microscopy at 40× magnification.

### Crystal violet staining

Cells were seeded into 6-well plates at 100.000 cells/well density. After five days of DOX treatment cells were washed with pre-warmed PBS and the medium was renewed. The day after the navitoclax treatment started, it was given three times a week for two weeks at an IC30 concentration.

For crystal violet staining a stock solution of 0.5% (w/w) crystal violet (Merck, Darmstadt, Germany) was prepared in 25% methanol, from which a 10 × dilution working solution was prepared in 25% methanol. Cells were washed twice with PBS. The 6-well plates were placed on ice and the cells were fixed with ice-cold 100% methanol for 5–10 min. After fixation 1 ml crystal violet working solution was added to the cells at room temperature for 5–10 min. Cells were washed several times with distilled water and then dried overnight.

### Western blotting

Cells were lysed in a lysis buffer (50 mM HEPES, pH 7.3, 150 mM NaCl, 10% glicerin, 1% Triton X-100, 1 mM EDTA, 1.5 mM MgCl2) supplemented with protease inhibitors. Samples were separated by sodium dodecyl sulfate-polyacrylamide gel electrophoresis (SDS-PAGE) then transferred to polyvinylidene difluoride (PVDF) membrane (Bio-Rad, Hercules, California, USA). The membranes were incubated with CDKN1A (p21), LMNB1 or Lamin B1 (Cell Signaling Technology, Danvers, MA, USA), Bcl-2 (Thermo Fisher, Waltham, MA, USA) and Bcl-XL (Proteintech, Rosemont, USA) primary antibodies at 4°C overnight and then incubated with HRP-conjugated secondary antibodies (List of all antibodies used in this study can be found in the Supplementary Material 1). Signals were detected using an ECL chemiluminescent detection system (WesternBright ECL kit, Advansta, San Jose, USA) and a Chemidoc MP device (Bio-Rad, Hercules, California, USA). The relative expression level of each protein was determined by densitometric analysis using Image Lab software (Bio-Rad, Hercules, California, USA).

### Statistical analysis of in vitro experiments

All experiments were repeated with a minimum of three biological replicates. For statistical analysis one-way ANOVA or unpaired t-test was performed on GraphPad Prism version 8.0.1 (GraphPad Software, Boston, Massachusetts USA). n.s. not significant; ^∗^ *p* ≤ 0.05, ^∗∗^ *p* ≤ 0.01, ^∗∗∗^ *p* ≤ 0.001, ^∗∗∗∗^ *p* ≤ 0.0001.

### In vivo experiments

All animal protocols were approved by the Hungarian Animal Health and Animal Welfare Directorate according to the EU’s most recent directives. All surgical procedures were performed according to the Committee on the Care and Use of Laboratory Animals of the Council on Animal Care at the Department of Experimental Pharmacology, National Institute of Oncology, Budapest, Hungary (PEI/001/1738–3/2015 and PE/EA/1461–7/2020).

1 mm^3^ cubes of Brca1^−/−^;p53^−/−^FVB mouse mammary tumors (a kind gift from Sven Rottenberg, NKI) were transplanted orthotopically into the mammary fat pad of wild type female FVB mice (Department of Experimental Pharmacology, National Institute of Oncology, Budapest, Hungary) under anesthesia (20 mg/kg zolazepam, 12.5 mg/kg xylazine, 3 mg/kg butorphanol, 20 mg/kg tiletamine) [28]. The tumor size was monitored at least 3 times per week by caliper measurements after the tumors became palpable. Tumor volume was calculated using the V = (length × width^2^)/2 formula. When the volume of the tumors reached ∼200 mm^3^, DOXIL treatment was initiated using the maximum tolerable dose (MTD, 6 mg/kg iv respectively). Two days after the DOXIL treatment, Navitoclax treatment was initiated, administered three times a week for two weeks at a dose of 50 mg/kg. Animals were sacrificed when the tumor volume reached ∼2000 mm^3^.

Kaplan–Meier survival curves were compared by Log-rank (Mantel-Cox) test.

### RNA isolation and transcriptome analysis

MCF7, T47D, MDA-MB-231 and Hs578T TIS cells were harvested at day 12, HFF TIS cells were harvested at day 24. CTR and REPOP cells were harvested at ~ 80% confluency. Cells were homogenized in TRIzol™ Reagent (Thermo Fisher Scientific, Massachusetts, USA). Total RNA was extracted from samples using Direct-zol® MiniPrep kit (Zymo Research, Irvine, California, USA) following the manufacturer's instructions. To prevent DNA contamination, an in-column DNAse I treatment was performed.

The prepared total RNA samples were sent to Xenovea Ltd. (Szeged, Hungary) for transcriptome analysis. The RNA concentration was determined by using the Qubit RNA HS Assay Kit on the Qubit 3.0 Fluorometer (Thermo Fisher Scientific, Waltham, MA, USA). Quality CTR was assessed by Labchip GX Touch HT instrument on DNA 5K/RNA/CZE Chip (Perkin Elmer, Waltham, MA, USA) with RNA Pico Sensitivity Assay Reagents (Perkin Elmer, Waltham, MA, USA). NextFlex PolyA beads 2.0 kit and NextFlex Rapid Directional RNA-seq Kit 2.0 with UDIs (Perkin Elmer, Waltham, MA, USA) were used for mRNA capture and strand-specific library preparation. The library quantities were measured by Quant-iT 1 × dsDNA HS Assay kit (Thermo Fisher, Waltham, MA, USA) with Fluostar Omega (BMG Labtech, Ortenberg, Germany). The fragment size distribution of the libraries was determined by capillary electrophoresis on Labchip GX Touch Nucleic Acid Analyzer on XMark HT chip by using DNA NGS 3K Assay kit (Perkin Elmer, Waltham, MA, USA). Pooled libraries were sequenced with 50 M 150 bp paired-end reads on NovaSeq 6000 platform (Illumina, San Diego, CA, USA).

The raw reads were preprocessed with Fastq Toolkit (v2.2.5) on Basespace Sequence Hub (Illumina), to remove low quality bases (< Q30) on 5'and 3'ends, trimming adapters (using sequences AGATCGGAAGAGCACACGTCTGAACTCCAGTCA,AGATCGGAAGAGCGTCGTGTAGGGAAAGAGTGT), and filter reads with mean read quality less than Q30. Splice aware aligner STAR (v2.7.10) was used to map the filtered, trimmed reads to genome assembly hg38. Gene counts were obtained by Subread's (v2.0.3) Feature counts function. Post-processing on the count matrix was executed using the R language (v4.3.3) [R core team]. The numeric matrix of read counts were preprocessed by edgeR (v4.0.16): genes with sufficiently large counts were retained after which the library sizes were recalculated and normalized. Next limma (v3.58.1) was used to transform the recalculated count data to log2-counts per million (logCPM) and to estimate the mean–variance trend for linear modeling. Throughout the rest of the analysis, the term'gene expression'refers to the logCPM values. Differentially expressed genes were calculated by the standard limma pipeline. First, a linear model was fitted for each gene using weighted least squares. Next, contrasts of interest (i.e. TIS vs CTR, REPOP vs TIS or REPOP vs CTR) were estimated. Finally, empirical Bayes smoothing of standard errors were applied. Differentially expressed genes were selected based on their log2 fold change (LFC) and Benjamini–Hochberg false discovery rate adjusted p-value (adj.p.val). A gene was designated as significantly differentially expressed if |LFC|> = 1 and adj.p.val < 0.05.

Overall similarities and differences in gene expressions were visualized by boxplots, heatmaps and principal component analysis (PCA) plots. Differentially expressed genes between two samples were visualized by a volcano plot. With more than two comparisons, Venn diagrams or upset graphs were used for visualization.

Gene set enrichment analysis (GSEA) was conducted by the fgsea (v1.28.0) package.

R Markdown (v2.28) code to reproduce results in Figs. 4 B-I, L-M, O, S3 C-G and S4 A is available at https://github.com/lovircsa/TIS.

### Single-cell RNA sequencing

MCF7 and T47D CTR, TIS (at day 12) and REPOP cells were collected by trypsinization, and 15.000 cells/samples were used. Two biological replicates of single-cell suspensions were prepared using Scipio Bioscience’s reversible hydrogel technology RevGel-seq™ with the instrument-free 3’ scRNA-seq benchtop kit Asteria™, allowing for coupling of solid polymer-barcoded beads and cells in a homogenous phase. Cell capture was followed by cell lysis, mRNA capture on barcoded beads, reverse transcription, PCR amplification and cDNA sequencing. The samples were sequenced to yield approx. 35.000 raw reads per analyzed cell.​​

Single-cell RNA sequencing (scRNA-seq) data were processed by aligning reads to the Human_Scipio_2022_A genome using the Cytonaut platform (v2.1.0). The resulting output files — features.tsv.gz, barcodes.tsv.gz, and matrix.mtx.gz — were imported into R for further analysis using the Seurat package (v5.1.0). To eliminate potential doublets, each sample was analyzed individually. Low-quality or dying cells often exhibit extensive mitochondrial contamination. Therefore, the PercentageFeatureSet() function was used to calculate mitochondrial indicators, and abnormal cells were subsequently removed based on a specified cutoff, which was determined as follows. Cells expressing fewer than 500 genes or fewer than 800 unique molecular identifiers (UMIs) were removed, along with those with over 10% mitochondrial content. The remaining cells were normalized and scaled using Seurat’s NormalizeData and ScaleData functions. Variable genes were identified using FindVariableFeatures, followed by principal component analysis (PCA). The optimal number of principal components (PCs) was determined using the ElbowPlot method, with PCs selected for each sample as follows: MCF7_CTR_1: PCs 1–15, MCF7_CTR_2: PCs 1–15, MCF7_TIS_1: PCs 1–17, MCF7_TIS_2: PCs 1–19, MCF7_REPOP_1: PCs 1–17, MCF7_REPOP_2: PCs 1–15, T47D_CTR_1: PCs 1–16, T47D_CTR_2: PCs 1–17, T47D_TIS_1: PCs 1–12, T47D_TIS_2: PCs 1–15, T47D_REPOP_1: PCs 1–17, T47D_REPOP_2: PCs 1–17. Cell clustering was conducted using the FindClusters function with a resolution of 0.8, and dimensionality reduction was performed using the RunUMAP function to generate UMAP embeddings. To identify and remove doublets, the DoubletFinder package (v2.0.4) was used. For each sample, pK (a tunable parameter representing the neighborhood size used for k-nearest neighbor clustering in DoubletFinder) selection was performed using the paramSweep function, and the optimal pK value was determined using the find.pK function, selecting the value corresponding to the maximum BCmetric score. The expected doublet rate was set to 7.5%, with an adjusted expected doublet count calculated using the modelHomotypic function to correct for homotypic doublet formation. The doublet classification step was performed using the doubletFinder function, with the following parameters: pN = 0.25, pK = sample-specific value, and nExp = adjusted expected doublet count. Following classification, cells labeled as "Doublet" were removed from each dataset.

### MCF7 and T47D sample processing for UMAP and downstream analysis

Following the removal of doublets, cells from MCF7 and T47D samples were processed separately, with all cells from each sample type merged into a single Seurat object. Further filtering was applied to remove cells with more than 10% mitochondrial RNA, more than 30,000 UMIs, fewer than 500 unique genes, or fewer than 1,000 UMIs. Standard Seurat processing was performed, starting with NormalizeData (using the LogNormalize method and a scale factor of 10,000). Variable genes were identified with FindVariableFeatures. The data was then scaled using ScaleData, and principal components were computed via RunPCA. Batch effect correction was not applied in this analysis. The samples exhibited high consistency, and applying batch correction could have undesirably diminished the biological information. Clustering analysis was conducted using FindNeighbors (first 15 principal components for MCF7 and first 20 principal components for T47D) and FindClusters, with clustering resolutions set to 0.3 for MCF7 and 0.5 for T47D samples. Finally, RunUMAP was used to calculate UMAP embeddings based on the first 15 principal components for MCF7 and the first 20 principal components for T47D. To assess cell cycle stages, CellCycleScoring in Seurat was used to calculate G2/M and S phase scores. Differential gene expression was analyzed using the Seurat function FindMarkers. For each comparison, ident.1 was set to the sample of interest, ident.2 was set to the background or control sample, and the Wilcoxon rank sum test ("wilcox") was used as the differential test. To account for multiple testing, adjusted p-values were calculated using the Bonferroni correction based on all genes in the dataset. A threshold of 0.05 was applied to the adjusted p-values (padj) to determine statistical significance. The gene set enrichment analysis (GSEA) was performed using clusterProfiler package (v4.6.2). For the combined analysis of MCF7 and T47D samples, cells from both datasets were merged into a single Seurat object. The same Seurat processing pipeline was applied, utilizing the first 15 principal components for both the FindNeighbors and RunUMAP steps. Clustering was performed with a resolution parameter set to 0.5.

#### Trajectory analysis

Monocle3 (v1.3.1) begins by projecting cells into a low-dimensional space that represents their transcriptional states, using UMAP. Next, it clusters similar cells through the Louvain community detection algorithm, which identifies distinct groups. These groups are then merged into larger "supergroups". Finally, Monocle 3 maps the developmental trajectories of individual cells within these supergroups, pinpointing key locations where cells branch or converge. Two distinct trajectories were generated for both MCF7 and T47D cell lines: one from the control population to TIS (therapy-induced senescence), and the other from TIS to repopulation. Genes that changed along these trajectories were identified, and the top 200 genes from each trajectory were selected. The top 200 genes from MCF7 control to TIS were then compared with the top 200 from T47D control to TIS. A similar comparison was performed for the TIS to repopulation trajectory. Next, differential gene expression was analysed for both MCF7 control vs. TIS and T47D control vs. TIS. The gene lists from both comparisons were overlapped, and this overlap was further compared with the overlapping trajectory genes. The final gene list included 92 genes, with CDKN1A added manually. Pseudobulk counts were generated for MCF7 and T47D, and TMM-normalized FPKM counts were produced. These were then cross-referenced with the earlier gene lists. After Z-score normalization, the gene expression data was visualized using a heatmap.

### Surface and secreted proteins/peptides characterization

To identify the targetable proteome on the surface of the CTR, TIS (at day 12) and REPOP MCF7 cells, we used a high-throughput surface biotinylation method similarly as described in the previous works [29, 30]. Parental and repopulated cells were cultured until they reached ~ 80% confluency before labeling, while TIS cells were processed on day 7 after doxorubicin removal. First, the culture medium was discarded, and the stage-specific cells were washed with pre-warmed PBS (137 mM NaCl, 2.7 mM KCl, 10 mM Na_2_HPO_4_ and 1.8 mM KH_2_PO_4_; pH 7.4) three times. The surface proteins of the cells were labeled by 2 mM Sulfo-NHS-SS-biotin at room temperature in PBS (at pH 8.0) for 20 min. We have systematically optimized these labeling conditions (PBS buffer, pH = 8.0, room temperature, incubation time) to maximize the biotinylation of cell surface proteins while maintaining cell viability. Over 99% of cells remained intact, as confirmed by trypan blue staining and propidium iodide uptake assays, consistent with our previous studies [29, 30]. Furthermore, the labeled transmembrane protein segments were verified to be topologically correct — i.e., labeling occurred exclusively on extracellular domains — based on data from the UniTmp database [31]. This confirms that the membrane-impermeable agent did not cross the cell membrane, a point also reinforced by a recent study [32]. The biotinylation process was stopped by Tris buffered saline (TBS: 25 mM Tris base, 150 mM NaCl, pH 7.2), the solutions were discarded and the cells were washed again three times with TBS. The cells were scraped into an ice-cold hypotonic lysis buffer (20 mM Tris–HCl, 10 mM KCl, 20 mM sucrose, 10 mM iodoacetamide (IA), pH 7.4). The cells were lysed on ice manually using a plastic micro pestle and a 1 mL syringe with 26-gauge ½ inch needle. A micro pestle was applied at least 40 times with a 180° rotation in both directions within an Eppendorf tube. Subsequently, cell lysis was facilitated using a ½ inch, 26-gauge needle with a 1 ml syringe, where the solution was aspirated up and down 40 times to ensure thorough disruption. The remained intact cells and cell debris and nuclei were pelleted at 1700 × g for 5 min at 4°C, and the supernatant was transferred into a 10.4 mL polycarbonate tube and centrifuged at 100,000 × g for 1 h at 4°C using a 70.1 Ti fixed rotor (Beckman Coulter). Biotinylated proteins were enriched in the pellet fractions, and resuspended in the 10-times diluted lysis buffer and homogenized by 25 strokes with a Potter–Elvehjem PTFE pestle in a glass tube on ice, finally stored at − 20°C. The protein concentration of the membrane preparations was measured by the Lowry method.

Membrane preparations with same protein content were solubilized in the presence of 0.1% (w/v) Rapigest SF Surfactant and the solutions were supplemented with 1.25 mM iodoacetamide and 1.25 mM 2,2′-Thiodiethanol. Denatured proteins were digested overnight (~ 16 h) at 37°C with proteomics grade trypsin, in a 1:50 (w/w) enzyme-to-protein ratio. Digestion was stopped by heat inactivation at 95°C for 10 min, thereafter the biotinylated surface peptides were pulled down neutravidin agarose resin for 1 h at room temperature. Non-specific peptides were removed by several washing steps. The biotinylated peptides were eluted by 10 mM Dithiothreitol in 50 mM NH_4_HCO_3_buffer using two consecutive incubations of 30 min, each at 37°C. The fractions were combined and alkylated with 25 mM iodoacetamide in dark at 37°C for 45 min. The solutions were dried in a pre-heated vacuum concentrator, then the peptides desalted with a reversed-phase C18 spin column as described in a previous work [30]. The samples were dried again and stored at − 20°C until further use.

### Mass spectrometry

All measurements were carried out on a Waters ACQUITY UPLC M-Class LC system (Waters, Milford, MA, United States) coupled with an Orbitrap Exploris 240 mass spectrometer (Thermo Fisher Scientific, Waltham, MA, United States). Symmetry C18 (100 Å, 5 µm, 180 µm × 20 mm) trap column was used for trapping and desalting the samples. Chromatographic separation of peptides was accomplished on an ACQUITY UPLC M-Class Peptide BEH C18 analytical column (130 Å, 1.7 µm, 75 µm × 250 mm) at 45°C by gradient elution. Water (solvent A) and acetonitrile (solvent B), both containing 0.1% formic acid were used as mobile phases at a flow rate of 200 nL/min. The sample temperature was maintained at 5°C. The mass spectrometer was operated using the equipped Nanospray Flex Ion Source. Data were collected using the data-dependent acquisition (DDA) method with MS1 scan between 360 and 2200 Th using 60,000 resolution, while ddMS2 scans with isolation windows of 2 Th were collected at 30,000 resolution keeping a 3 s cycle time. Data acquisition was performed using XcaliburTM 4.6 (Thermo Fisher Scientific, Waltham, MA, United States). Raw LC–MS data files were processed using Fragpipe v22.0 [33]. Uniprot Human reference proteome assuming 2 missed cleavage sites was used (20,575 proteins) for protein identification. An initial open search (using default settings) was performed to identify detectable peptide modifications. The final quantitative analysis was performed assuming Met oxidation, 3-(carbamidomethylthio)propanoyl Lys (effect of biotin labelling) and pyro Glu as variables and carbamidomethyl Cys as fixed modification. Protein quantification was performed within Fragpipe using IonQuant with default settings, and enabling match between runs. A contaminant database was created using a database search of LC–MS data collected from a labeled and enriched digest of cell medium using the same search settings and Uniprot Bovine reference proteome database. Contaminant proteins and proteins with only one identified peptide were removed from the dataset before statistical analysis. Statistical analysis and visualization of proteomics data was performed in Perseus 1.6.15 [34] and Instantclue v0.12.2 [35]. Protein and peptide intensity data were log2 transformed and median normalized before statistical analysis. Differential expression analysis (DEA) was performed in Perseus using the built in Student’s t-test with permutation-based false discovery rate (FDR) estimation. A limit of FDR < 0.05 was applied using the Significance Analysis of Microarrays (SAM) method with a s0 = 0.1 value (approximate weight of mean difference in FDR calculation) using 250 randomizations [36]. In order to confirm protein level changes, DEA was also performed on individual peptides using the same approach. To confirm cell surface/extracellular presence of proteins the number of biotin labelled peptides were calculated for each identified protein. Proteomics raw data, protein and peptide level DEA results are available online (see ‘Data and code availability’ section).

### qRT-PCR validation

Total RNA was extracted using the Qiagen RNeasy Mini Kit, and 1 µg of total RNA was reverse transcribed with the High-Capacity cDNA Reverse Transcription Kit (Thermo Fisher Scientific) following the manufacturer’s protocol. Quantitative SYBR Green-based qRT-PCR assays were performed in quadruplicates using the PCRBIO HS Taq Mix Red on a QuantStudio 7 Flex Real-Time PCR System (Applied Biosystems). Gene expression data were analyzed using the ΔΔCt method. ACTB was used as the housekeeping gene for internal control and normalization. Primer sequences used for relative quantification can be found in Supplementary Table S1.

## Results

### Therapy surviving breast cancer cells enter senescence before repopulating

**Fig. 1:**
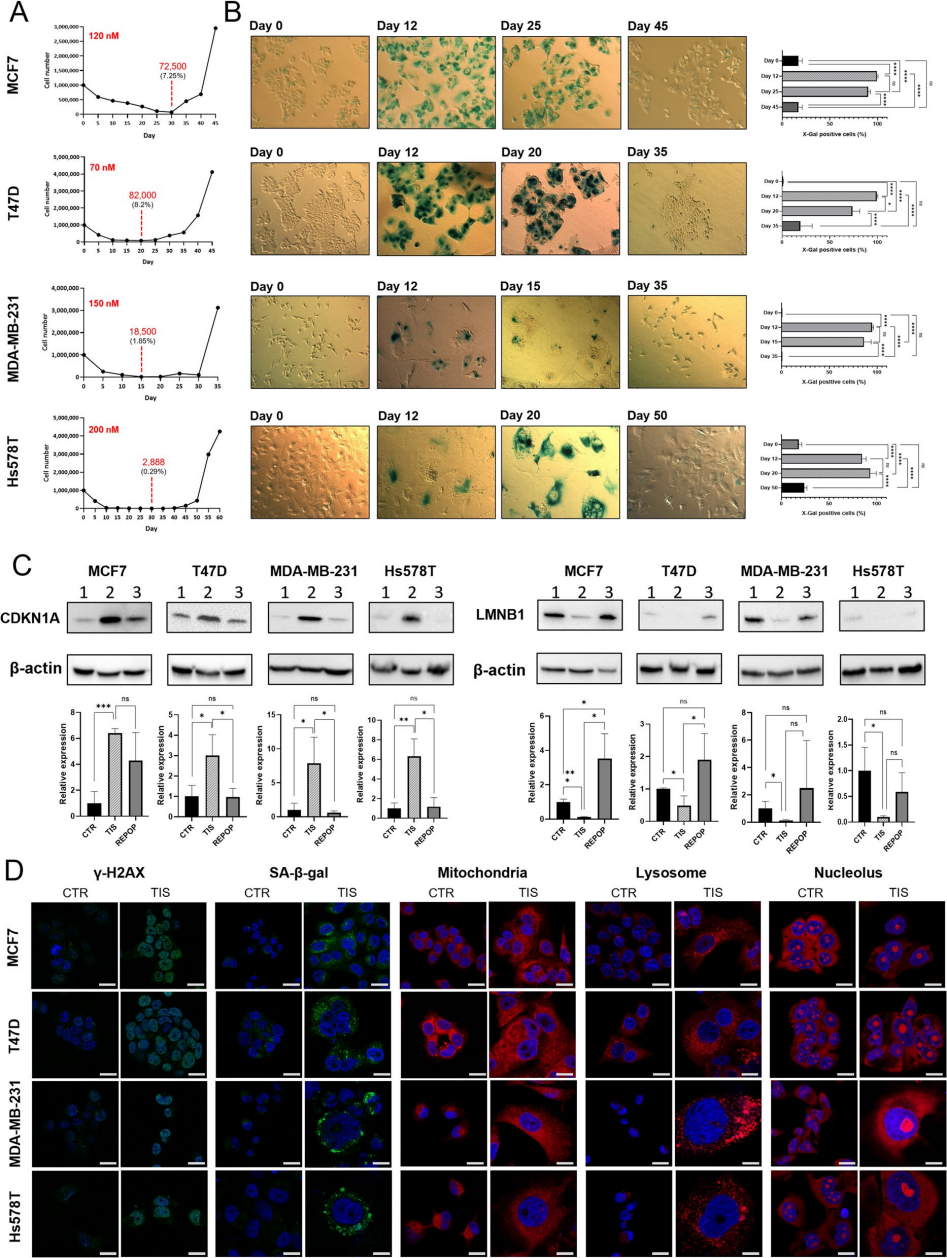
Chemotherapy-surviving cells transition into a transient senescent state. **A** Representative growth kinetics of cell cultures following 5-day doxorubicin (DOX) treatment. DOX was administered on day 0, and the medium was refreshed on day 5. The minimum cell counts are highlighted on the curves (red). **B** X-Gal staining of control (CTR), therapy-induced senescent (TIS), and repopulated (REPOP) cells, accompanied by quantification of staining intensity.**C** Western blot analysis of senescence marker CDKN1A and LMNB1 protein expression in CTR, TIS, and REPOP cells, with quantification of relative protein levels. **D** Fluorescence microscopy detection of DNA damage (γ-H2AX), senescence-associated β-galactosidase (SA-β-Gal) activity, mitochondria, lysosomes, and nucleoli in CTR and TIS cells. Scale bar: 20 µm

**Fig. 2:**
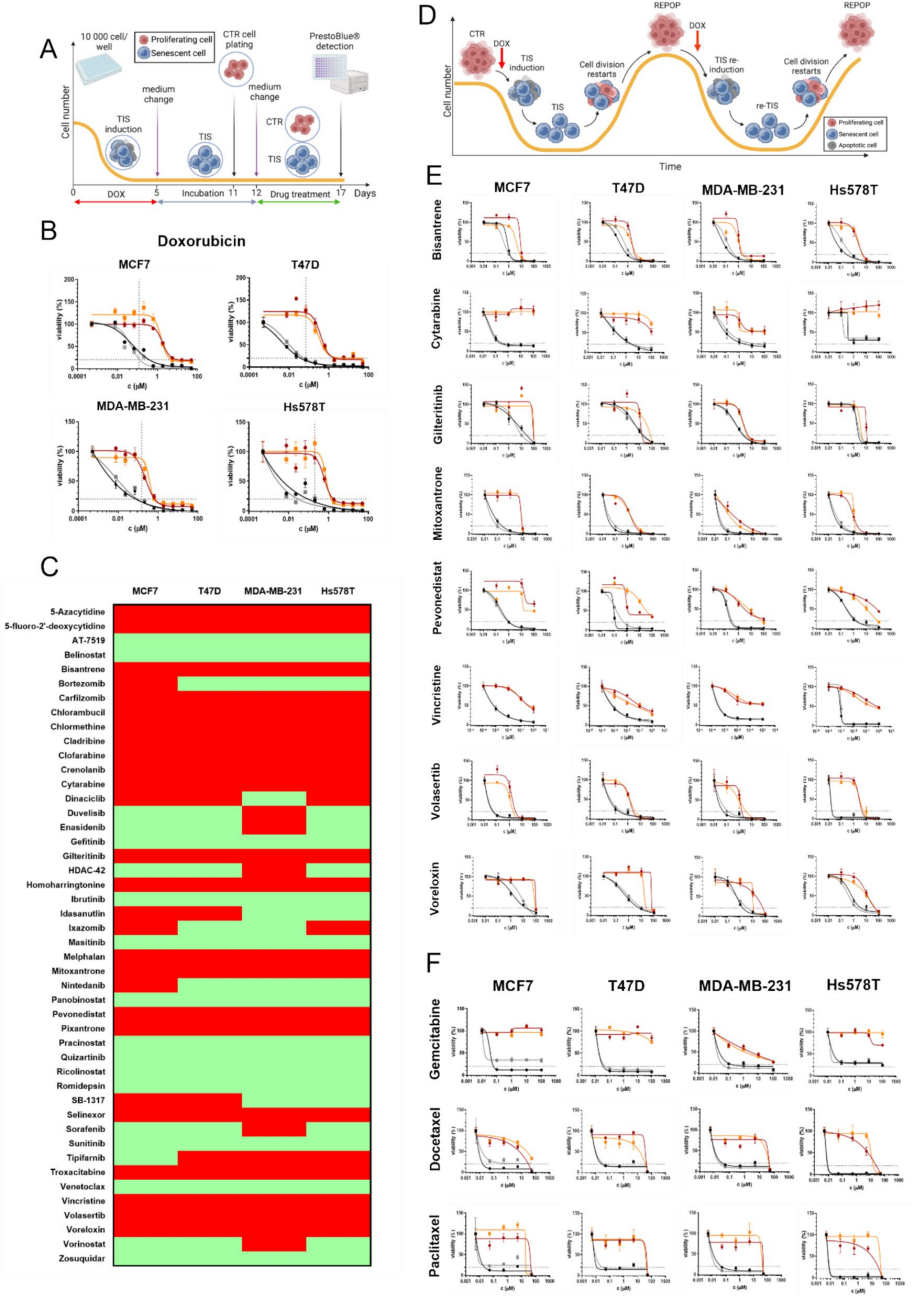
Therapy-induced senescent (TIS) cells exhibit resistance to a broad spectrum of compounds with diverse mechanisms of action. **A** Schematic representation of the experimental workflow, including senescence induction and subsequent drug screening assays. **B** Doxorubicin (DOX) sensitivity profiles of control (CTR, black), TIS (red), repopulated (REPOP, gray), and re-induced senescent (re-TIS, orange) cells. **C** Heatmap illustrating drug sensitivity to 46 FDA-approved anticancer compounds across the four breast cancer cell states. Red indicates at least a threefold increase in resistance of TIS cells relative to CTR cells, while green denotes no significant difference in sensitivity between CTR and TIS cells. **D** Schematic summary of all cell states (CTR, TIS, REPOP and re-TIS) investigated in the cytotoxicity experiments. **E** Dose–response analysis of CTR (black), TIS (red), REPOP (gray), and re-TIS (orange) cells treated with 8 selected compounds. **F** Sensitivity profiles of CTR (black), TIS (red), REPOP (gray), and re-TIS (orange) cells treated with clinically relevant breast cancer therapies

To replicate the clinically observed therapy response of tumors and to reproducibly establish treatment surviving breast cancer cells, we utilized an in vitro model previously developed in our laboratory for enriching drug tolerant persister cells following chemotherapy [37]. Four breast cancer cell lines, representing different molecular subtypes and characteristics were treated with high-dose doxorubicin (DOX), selected to kill > 90% of the cells (120 nM for MCF7, 70 nM for T47D, 150 nM for MDA-MB-231 and 200 nM for Hs578T, derived from Fig. 2B) via apoptosis (Figure S2), leaving only a small fraction of the population alive (Fig. 1A). Only approximately 7.25% MCF7, 8.2% T47D, 1.85% MDA-MB-231 and 0.29% Hs578T of the cells were able to evade apoptosis, while exhibiting prominent morphological and molecular characteristics of senescence. Most of the cells and their nuclei were enlarged, the cell bodies became flattened, and stained positively for senescence-associated ß-galactosidase (SA-ß-Gal) substrate X-gal (Fig. 1B). We also observed the overexpression of the cyclin-dependent kinase inhibitor protein CDKN1A (p21) [38] and the downregulation of the nuclear lamina building block LMNB1 [39], both widely used markers for the identifying senescent cells (Fig. 1C). Additionally, the cells exhibited extensive DNA damage, increased mitochondrial and lysosomal mass, and developed a single, fused nucleolus (Fig. 1D). Surprisingly, despite senescence being considered irreversible, a small number of cells (0.00001 - 0.00004%) managed to escape TIS in each cell line, leading to repopulation in all experiments. In these REPOP cells, the senescent phenotype was reversed, and proliferation rapidly resumed, suggesting that in breast cancer cells TIS is only transient.

### TIS breast cancer cells possess a unique pattern of drug resistance and sensitivity

Since chemotherapy is administered to patients in repeated cycles, we evaluated how TIS cells respond to a second dose of DOX 12 days after the initial treatment (Fig. 2A). Surprisingly, these cells demonstrated significant resistance to the repeated treatment. However, once the cells escaped senescence and repopulated, the DOX-resistant phenotype was reversed (Fig. 2B). Additionally, when REPOP cells were re-treated with DOX, they re-entered senescence and regained drug resistance, indicating that even after re-sensitization, TIS provides substantial protection against repeated treatments. To investigate cross-resistance to other drugs, we screened 63 FDA-approved compounds with various mechanisms of action and mapped the resistance/sensitivity profile of TIS cells. Resistance to antimetabolites was a common feature, as 5-azacytidine, 5-fluoro-2’-deoxycytidine, cladribine, cytarabine, clofarabine and troxacitabine were all ineffective at killing TIS cells (Fig. 2C, Table S2). Inhibition of topoisomerase II (mitoxantrone, pixantrone, voreloxin) and polo-like kinase 1 (volasertib) was also ineffective, as was DNA alkylation by agents such as chlormethine, chlorambucil, and melphalan. The TIS phenotype also showed resistance to the neddylation inhibitor pevonedistat, the farnesyltransferase inhibitor tipifarnib and the FLT3 inhibitor gilteritinib. However, surprisingly, TIS cells remained sensitive to quizartinib, another FLT3 inhibitor. In contrast, certain compounds were effective across all cell states, including CTR, TIS and REPOP. Given the reliance of breast cancer cells on histone deacetylation mediated epigenetic regulation, the response to HDAC inhibitors (belinostat, HDAC- 42, panobinostat, pracinostat, ricolinostat, romidepsin, vorinostat) was similar between CTR and TIS cells, as was the response to the PI3K inhibitor duvelisib and the proteasome inhibitors bortezomib and ixazomib. However, TIS cells were universally resistant to another proteasome targeting drug, carfilzomib. This unique drug resistance/sensitivity profile cannot be attributed solely to the lack of proliferation, as other compounds targeting rapidly dividing cells were still able to kill TIS cells. For example, while TIS cells were resistant to dinaciclib, the multi-CDK inhibitors AT7519 and SB-1317 which require an active cell cycle, were toxic to both proliferating and non-proliferating cells. Similarly, Bruton's tyrosine kinase inhibitor ibrutinib was toxic to both CTR and TIS cells across all cell lines. Multi-tyrosine kinase inhibitors generally showed toxicity to both non-senescent and TIS cells with masitinib, nintedanib, sorafenib and sunitinib killing nearly all cell types in at least three cell lines, while TIS cells remained completely resistant to crenolanib. Overall, 17 compounds were non-toxic at the tested concentrations, but of the remaining 46, TIS cells exhibited resistance to 23 drugs in at least three cell lines, indicating that TIS represents a transient yet significant phenotype of drug resistance.

To demonstrate that TIS alone can protect cancer cells repeatedly from subsequent rounds of treatment without the emergence of any other drug resistance mechanism, REPOP cancer cells were re-treated with DOX to induce TIS again (re-TIS, Fig. 2D). The cells were then exposed to 15 selected compounds to assess drug resistance (Fig. 2E, Figure S3A). The re-TIS cells displayed almost identical resistance profiles to original TIS cells. Compounds that were ineffective against TIS cells in all cell lines but were not tested on re-TIS cells shown in Figure S3B. Meanwhile drugs that exhibited limited efficacy in killing TIS cells in three or two out of four cell lines are detailed in Figure S3 C and D, respectively.

Finally, we tested drugs routinely used in breast cancer treatment in the same manner (Fig. 2F). The antimetabolite gemcitabine, along with the taxanes paclitaxel and docetaxel — cornerstones of breast cancer therapy for decades — effectively eliminated both CTR and REPOP cells, but were ineffective against the TIS and re-TIS phenotype.

### TIS breast cancer cells are only partially sensitive to Bcl-2 inhibition

**Fig. 3:**
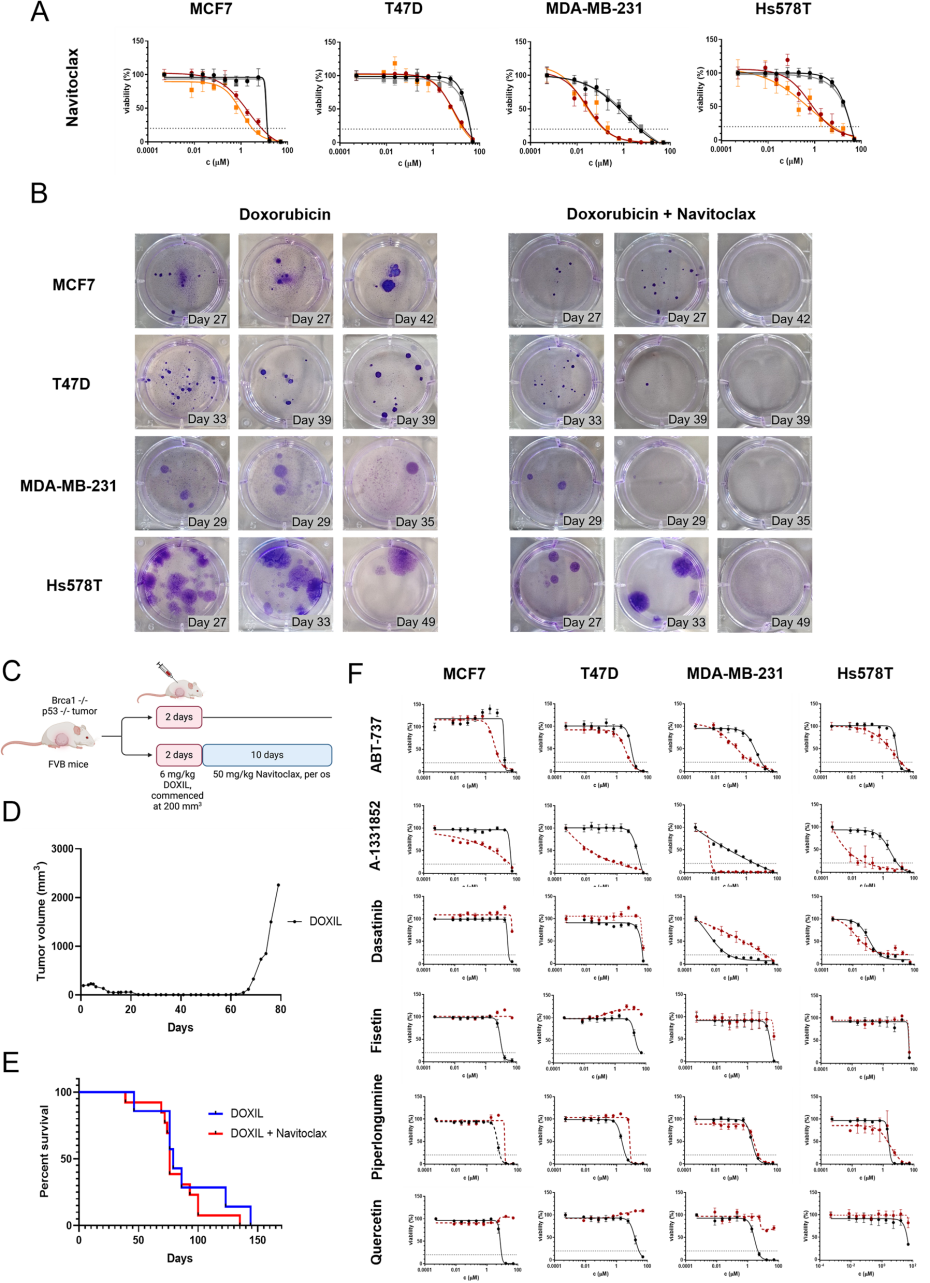
Therapy-induced senescence (TIS) breast cancer cells exhibit partial sensitivity to senolytic treatment.** A** Navitoclax sensitivity profiles of control (CTR, black), TIS (red), repopulated (REPOP, gray), and re-induced senescent (re-TIS, orange) cells. **B** Crystal violet staining of breast cancer cell cultures treated with doxorubicin (DOX) alone or in combination with Navitoclax (DOX + Navitoclax). **C** Schematic representation of the experimental design comparing the effects of DOXIL versus DOXIL + Navitoclax treatment in an in vivo model. **D** Representative tumor growth curve of Brca1-/-;p53-/- tumors treated with the maximum tolerated dose of DOXIL. **E** Kaplan–Meier survival analysis of Brca1-/-;p53-/- tumor-bearing mice treated with DOXIL (*n* = 7) or DOXIL + Navitoclax (*n* = 13). Statistical analysis shows p = 0.3888. **F** Sensitivity analysis of CTR (black) and TIS (red) cells treated with six different senolytic compounds

To further investigate the TIS phenotype we tested navitoclax, a well-known Bcl-2 inhibitor, which earned wide recognition as a senolytic compound. While we confirmed the senolytic activity of navitoclax in cytotoxicity assays (Fig. 3A), long-term treatment produced ambiguous results (Fig. 3B, Figure S4 A). Given the ongoing debate over the specificity and selectivity of Bcl-2 inhibitors, we conducted an experiment combining DOX and navitoclax during senescence induction to investigate whether depleting the TIS cells could prevent repopulation. Interestingly, navitoclax had to be continuously administered from the onset of the treatment to significantly reduce relapse in our assay, suggesting that BCL2 overexpression may be an early response to DOX rather than a senescence-specific alteration. However, the outcomes varied widely, ranging from complete eradication of cells to no observable effect. In a clinically relevant mouse model of triple-negative breast cancer (TNBC, Fig. 3C), a single dose of pegylated liposomal DOX (DOXIL) induced complete tumor response, with no detectable or palpable tumors for 40–60 days, mirroring our in vitro findings (Fig. 3D). Despite this, navitoclax failed to extend survival or demonstrate any disease-modifying effect in this model (Fig. 3E). Senescent cells are hypothesized to evade apoptosis by overexpressing Bcl-2, an anti-apoptotic factor that inhibits pro-apoptotic proteins BAX and BAK. Therefore, inhibiting Bcl-2 should selectively induce cell death in senescent cells. To test this hypothesis, we applied several senolytic drug treatments in our cellular assay. TIS cells responded differently depending on the potency and selectivity of the tested molecules (Fig. 3F, Table S3). The selective Bcl-2 inhibitor venetoclax (from Fig. 2C) exhibited equal toxicity toward both CTR and TIS cells, while navitoclax, ABT-737 and A-1331852 — inhibiting Bcl-2/Bcl-XL/Bcl-w, Bcl-2/Bcl-XL and Bcl-XL, respectively, — were selectively toxic to TIS cells. This observation suggests that bypassing apoptosis in TIS could be driven by Bcl-XL rather than Bcl-2. While this explanation appeared plausible, repeated monitoring of Bcl-2 and Bcl-XL during and after DOX treatment revealed no correlation between Bcl inhibitor efficacy and protein expression levels (Figure S4B). Similarly, the localization and/or heterogeneity of these proteins did not support the link between Bcl-2 inhibition and viability (Figure S4C). Moreover, the mRNA expression levels of BCL2 and BCL2L1 were comparable across CTR, TIS, and REPOP cells (Figure S4D). Although compounds like dasatinib, fisetin, quercetin and piperlongumine have been reported to selectively kill senescent cells via different targets, TIS cells in our assays displayed resistance to these agents. To further support that apoptosis can be induced in TIS cells, we performed live-cell monitoring with Annexin V staining during belinostat treatment (Figure S4E,F). However, apoptotic pathways in TIS cells remained largely unchanged (Figure S4G,H).

### The TIS phenotype is transient and transcriptomically distinct compared to CTR and REPOP cells

**Fig. 4:**
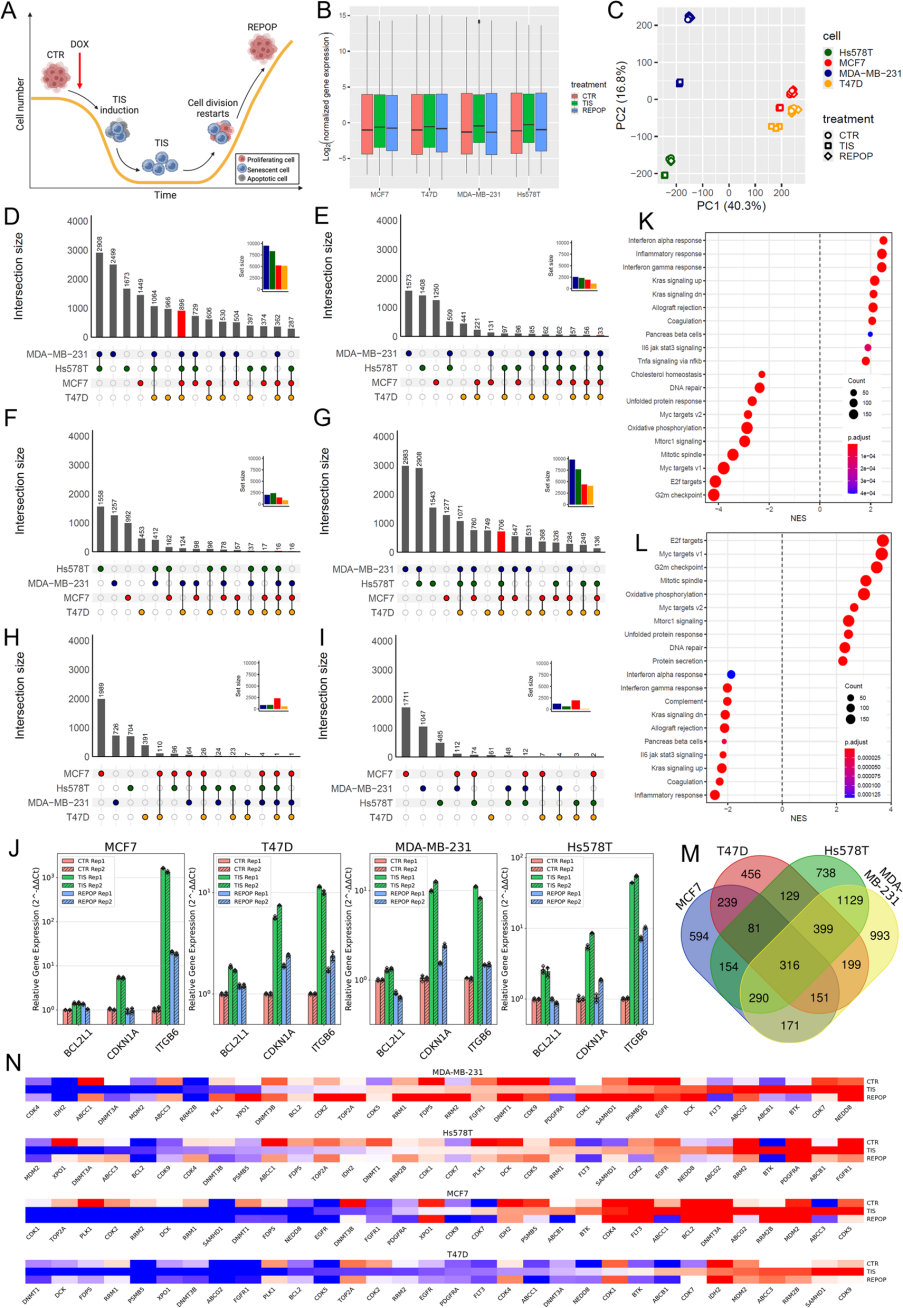
Therapy-induced senescence (TIS) cells display a unique gene expression profile. **A** Workflow of bulk RNA sequencing. Samples were collected from untreated control (CTR) cells on Day 0. Doxorubicin (DOX) treatment was applied (red arrow) for 5 days, and RNA was isolated from TIS cells on Day 12, 7 days post-drug removal. REPOP cells were harvested between Days 24–36 when culture confluency reached 80–90%. **B** Box plots representing global gene expression differences between TIS and CTR/REPOP cells across all cell lines. **C** Principal component analysis (PCA) of the gene expression data showing in the case of all four cell lines clustering of CTR and REPOP cells, which exhibit transcriptomic similarity, while TIS cells form a distinct cluster, highlighting their unique gene expression profile. **D**, **E** UpSet plots of differentially expressed genes (DEGs) from CTR vs. TIS comparisons, illustrating upregulated (**D**) and downregulated (**E**) genes. Set sizes are indicated, with red columns representing DEGs shared across all cell lines. **F**, **G** UpSet plots of upregulated (**F**) and downregulated (**G**) DEGs from TIS vs. REPOP comparisons, with set sizes and shared DEGs (red columns) shown. **H**, **I** UpSet plots of upregulated (**H**) and downregulated (**I**) DEGs from CTR vs. REPOP comparisons, with set sizes indicated. **J** Relative quantification of BCL2L1, CDKN1A, and ITGB6 expression in MCF7, T47D, MDA-MB-231, and Hs578T breast cancer cell lines under CTR, TIS, and REPOP conditions using qRT-PCR. The bar plot illustrates the relative mRNA expression levels (2^-ΔΔCt) of BCL2L1, CDKN1A, and ITGB6 across the four breast cancer cell lines under the three experimental conditions: CTR (red), TIS (green), and REPOP (blue), with biological replicates distinguished by solid fill (Rep1) and diagonal hatching (Rep2,///). Bars represent mean expression levels, with error bars indicating standard deviation, and individual technical replicates (n = 4 per condition) displayed as light gray diamond markers. The qRT-PCR measurements were performed in two biological replicates per condition, ensuring reproducibility. The y-axis is set to a symlog scale to accommodate the broad range of expression levels, and gene labels are rotated for clarity. **K**, **L** Pathway analysis based on RNA-seq data from MCF7, T47D, MDA-MB-231, and Hs578T cells, highlighting upregulated and downregulated pathways in CTR vs. TIS (**K**) and TIS vs. REPOP (**L**) comparisons. Top and bottom 10 pathways are shown based on the normalised enrichment score (NES). **M** Venn diagram of mRNAs significantly upregulated (log2 fold change > 1, adjusted *p*-value < 0.05) in the CTR-to-TIS transition and simultaneously downregulated (log2 fold change < 1, *p*-value < 0.05) in the TIS-to-REPOP transition. **N** Heatmap of scaled gene expression changes in genes associated with resistance to tested compounds. Expression values were centered and scaled for each gene across all samples

To characterize the TIS cells across all 4 breast cancer lines, we analyzed the differentially expressed gene (DEG) sets using bulk RNA sequencing (Fig. 4A). Normalized gene expression data revealed distinct differences between TIS samples and CTR and REPOP cells (Fig. 4B). This was further supported by PCA analysis, which clearly separated TIS cells from both CTR and REPOP cells, indicating that all cell lines responded similarly to DOX and developed the TIS phenotype in a consistent manner (Fig. 4C). DEG analysis revealed that while there were notable differences between the four cell lines, all TIS cells share 929 DEGs compared to CTR cells, regardless of their origin (Fig. 4D and E). Strikingly, the vast majority of these genes (896) were overexpressed, with only 33 (3.5%) being downregulated. In contrast, during the transition from TIS to REPOP, the expression of 722 genes changed, with only 16 (2.2%) being upregulated while the expression of 706 genes significantly decreased (Fig. 4F and G). By comparing DEGs between CTR and REPOP cells, we found only 1 gene that remained overexpressed and none that remained downregulated across all four cell lines, indicating that the original gene expression pattern was largely re-established in the REPOP cells (Fig. 4H and I). To confirm that the TIS state is transcriptionally transient, we analyzed the direction of gene signature changes. The transition from CTR to TIS induced a dramatic overexpression of genes, while the transition from TIS to REPOP showed the opposite effect (Figure S5). Furthermore, when we followed the expression of 13 genes that were significantly upregulated in TIS cells, we observed that in REPOP cultures these same genes exhibited the reverse trend, becoming downregulated. We further validated the RNA sequencing results using qPCR (Fig. 4J, Figure S6), expanding our analysis to include 11 selected genes. This set comprised senescence markers (CDKN1A, LMNB1, MKI67), apoptosis regulators (BCL2, BCL2L1), a highly overexpressed gene (ITGB6), and drug targets (ACTA2, GSDMC, KRT6A, PDE1A, PSMA8), all of which showed strong concordance with our RNA-seq data in all four breast cancer cell lines.

Given the significant similarities among TIS cells, across the four tested cell lines, we conducted gene set enrichment analysis on the mRNA expression data to identify common characteristics of the TIS phenotype (Fig. 4K). The analysis revealed that TIS cells: (1) are non-proliferative, as 5 out of 6 proliferation-related gene sets (G2M Checkpoint, E2F Targets, MYC Targets V1 and V2, Mitotic Spindle) from the “hallmark” sets of the Molecular Signatures Database (MSigDB), were significantly downregulated, with the exception of the p53 pathway, (2) rely on KRAS signaling, as gene sets comprising both up- and downregulated genes related to KRAS activation (KRAS Signaling UP and DN) were significantly upregulated, and (3) exhibit significantly reduced DNA repair capacity, as the DNA Repair gene set was downregulated. Additionally, all 7 immune-related hallmark gene sets (Allograft Rejection, Coagulation, Complement, Interferon alpha Response, Interferon gamma response, IL6-JAK-STAT3 Signaling, Inflammatory Response) were altered, suggesting that TIS may have a significant immune modulatory effect. After escaping TIS, all of these changes were reversed (Fig. 4L).

Senescence was also induced in healthy human foreskin fibroblast (HFF) cells using DOX to compare TIS-related changes in malignant and non-cancerous cells (Figure S7). While the treatment caused significant DNA damage in HFF cells (Figure S7 A), induced TIS in virtually all surviving cells (Figure S7B), the difference between CTR and TIS cells in the PCA analysis were less pronounced than it was observed in the case of breast cancer cell lines (Figure S7 C). Additionally, the distribution of up- and downregulated genes was more balanced in the HFF cells (Figures S7D). A comparison of hallmark pathways of MSigDB in the four cancer cell lines and in HFF cells revealed 11 shared gene sets (KRAS signaling up, KRAS signaling down, Coagulation, IL6-JAK-STAT Signaling, TNFa Signaling via NFκB, Unfolded protein response, Myc targets v2, Mtorc1 signaling, Mitotic Spindle, E2F targets, G2M checkpoint). However, one of these, TNFa Signaling via NFκB, showed changes in the opposite direction in non-malignant HFF cells (Figure S7E). A similar analysis focusing on biological processes (KEGG) identified five shared gene sets between TIS HFF and breast cancer cells that were altered in the same manner (Neuroactive ligand receptor interaction, Retinol metabolism, Cytokine cytokine receptor interaction, DNA replication, Cell Cycle), while the other 15 most differentially expressed pathways were dissimilar (Figure S7 F,G). Taken together, the analysis revealed that while HFF cells share some common TIS-related pathways with breast cancer cells, the response is less pronounced in non-malignant cells, with a more balanced gene regulation and some key pathways, such as TNFa signaling via NFκB, showing opposite trends. Additionally, significant differences were observed in 15 key biological processes between TIS in HFF and breast cancer cells.

To find the core gene set exclusive to the TIS cells, we identified genes that were transiently overexpressed only in TIS cells across all four cell lines (Fig. 4M). A total of 316 mRNAs which were upregulated in TIS but downregulated once the cells resumed proliferation (Figure S8 A). This TIS-specific gene set suggests that TIS cells secrete elevated levels of cytokines, likely due to the Senescent-associated Secretory Phenotype (SASP), rely on the JAK/STAT signaling pathway and retinol metabolism, and express a wide array of ABC transporters (Figure S8B).

While the transcriptomes of CTR and REPOP cells were highly similar, indicating that the REPOP cells indeed resemble their parental lines, we could identify residual gene expression changes that may serve as a molecular memory of the cells recovering from TIS. We selected the genes that were overexpressed (log2 FC > 1, adjusted p-value < 0.05) in TIS cells of the breast cancer cell lines and maintained these changes in the REPOP cells when compared to the CTR. As mentioned earlier, there was only one gene (IFIT1) that fulfilled these stringent conditions, but 22 genes fell into this category in at least 3 of the studied cell lines (Figure S8C). MDA-MB-231 cells appear to be outliers in this respect, but out of the 22, 12 genes from this group show similar changes in these cells, only with less pronounced expression changes or lower significance (Table S4). GO biological function analysis (Figure S8D) indicates that the genes in this category are related to interleukin and interferon signaling, indicating long-term changes in immunogenecity after TIS.

To determine whether TIS cells actively modulate their immune environment, we conducted a sensitive cytokine expression analysis, focusing on ten well-characterized immunomodulatory cytokines (Figure S9 A,B). In both MCF7 and T47D TIS cells, mRNA expression levels of IL2, IL2Rα, IL3, IL6, IL10, IL13, TNFα, IFNγ, and CSF2 were elevated compared to CTR cells, whereas IL4 expression remained negligible across both cell lines and cell states. Notably, IL2Rα, IL6, and IL13 exhibited robust upregulation in TIS cells from both cell lines, with 18-, 70-, and sevenfold increases in MCF7 and 11-, 32-, and fivefold increases in T47D, respectively. While T47D TIS cells displayed a pronounced increase in IL10 expression (19-fold), MCF7 TIS cells showed only a modest elevation (1.7-fold).

Functionally, several of these cytokines are well-documented mediators of immune suppression and tumor progression. IL6 plays a key role in senescence induction and tumor promotion by regulating myeloid-derived suppressor cells (MDSCs), which suppress T-cell-mediated antitumor immunity [40]. IL10 is a potent immunosuppressive cytokine with immune-regulatory and angiogenic properties, facilitating tumor cell survival, proliferation, and metastasis by inhibiting antitumor immune responses [41]. IL13 has been implicated in indirect immunosuppression through the promotion of tumor-associated macrophage (TAM) differentiation, leading to TGF-β secretion and the establishment of a tumor-promoting microenvironment [42].

Interestingly, the drug resistance observed in TIS cells can be explained only in a few cases with known mechanisms. We examined the expression levels of the usual drug resistance factors, such as efflux transporters, DNA repair mechanisms and genes which are targets of the screened compounds or known to inactivate drugs or counter their effects (Fig. 4N, Figure S10A). CDK1, 2, 4, 5, 7 and 9 are inhibited by AT7519, SB-1317 and dinaciclib, but the expression profile of these genes did not correlate with either resistance or sensitivity to CDK inhibitors. The loss or reduced expression of FDPS gene should have sensitized cells to tipifarnib [43], but TIS cells were showed resistance. Overexpression of the antiapoptotic Bcl-2 could explain TIS cell tolerance to many drugs and their sensitivity to venetoclax [44], but no such link was found. Increased expression of RRM1, 2 and 2B is often detected in DOX, gemcitabine and docetaxel resistant tumors [45], yet these genes were rather downregulated than overexpressed in TIS cells. SAMHD1 and DCK have been reported to mediate wide scale resistance to antimetabolites such as cytarabine, clofarabine and cladribine [46–48], but we found increased expression only of SAMHD1 and only in two cell lines in TIS cells. Investigating the expression of targets for biological therapies produced surprisingly mixed results. Targets of crenolanib/giltertinib/quizartinib (FLT3/PDGFRA, KIT), gefitinib (EGFR), ibrutinib (BTK), idasanutlin (MDM2), bortezomib/ixazomib (PSMB5) and nintedanib (FGFR1) were not upregulated in TIS cells. On the other hand, IDH2, the target of enasidenib, was downregulated in TIS cells, but not resulted in resistance as CTR and TIS cell were similarly sensitive to the drug. Similarly, despite TIS cells overexpressed NEDD8, the main mediator of neddylation, the cells were still resistant to the NEDD8 inhibitor pevonedistat. In two cases, the resistance against Selinexor and volasertib in TIS cells can be explained by the deregulation of their specific targets XPO1 and PLK1, respectively. Out of three investigated DNMTs, 1 and 3B were almost always downregulated in TIS cells, while decreased expression of 3A was found in two cell lines, suggesting a possible mechanism to reduce efficiency of DNMT inhibitors like 5-azacytidine and 5-fluoro-2’-deoxycytidine [49, 50]. Downregulation of TOP2A, which could be a key resistance mechanism against DOX, mitoxantrone, pixantrone and voreloxin, was detected in three cell lines. ABCB1, ABCC1, ABCC3 and ABCG2 drug transporters are known to recognize and expel a wide range of structurally and mechanistically diverse compounds [51]. ABCC1 and C3 were expressed cell line specifically, but ABCB1 – in accordance with our previous finding [37] – and ABCG2 were overexpressed in TIS cells in all cell lines. Surprisingly, due to the wide substrate specificity of ABCB1, TIS cells should have been resistant to belinostat, bortezomib, gefitinib, sorafenib and sunitinib too, a feature TIS cells clearly not possess, therefore we tested whether ABCB1 inhibition with tariquidar has any effect on DOX sensitivity (Figure S10B). This experiment proved that despite ABCB1 is significantly overexpressed in TIS in all cell lines, it's not enough to protect cells from drug treatment. Despite our extensive gene expression analysis, no discernible alterations were found in the classical resistance pathways or drug target genes that could account for the unique drug resistance and sensitivity profile observed in TIS cells, suggesting that the mechanisms underlying TIS-associated drug responses may involve non-canonical or context-dependent regulatory networks yet to be fully understood.

### Single-cell transcriptome profiling reveals TIS cells as a unique and distinct cell population

**Fig. 5:**
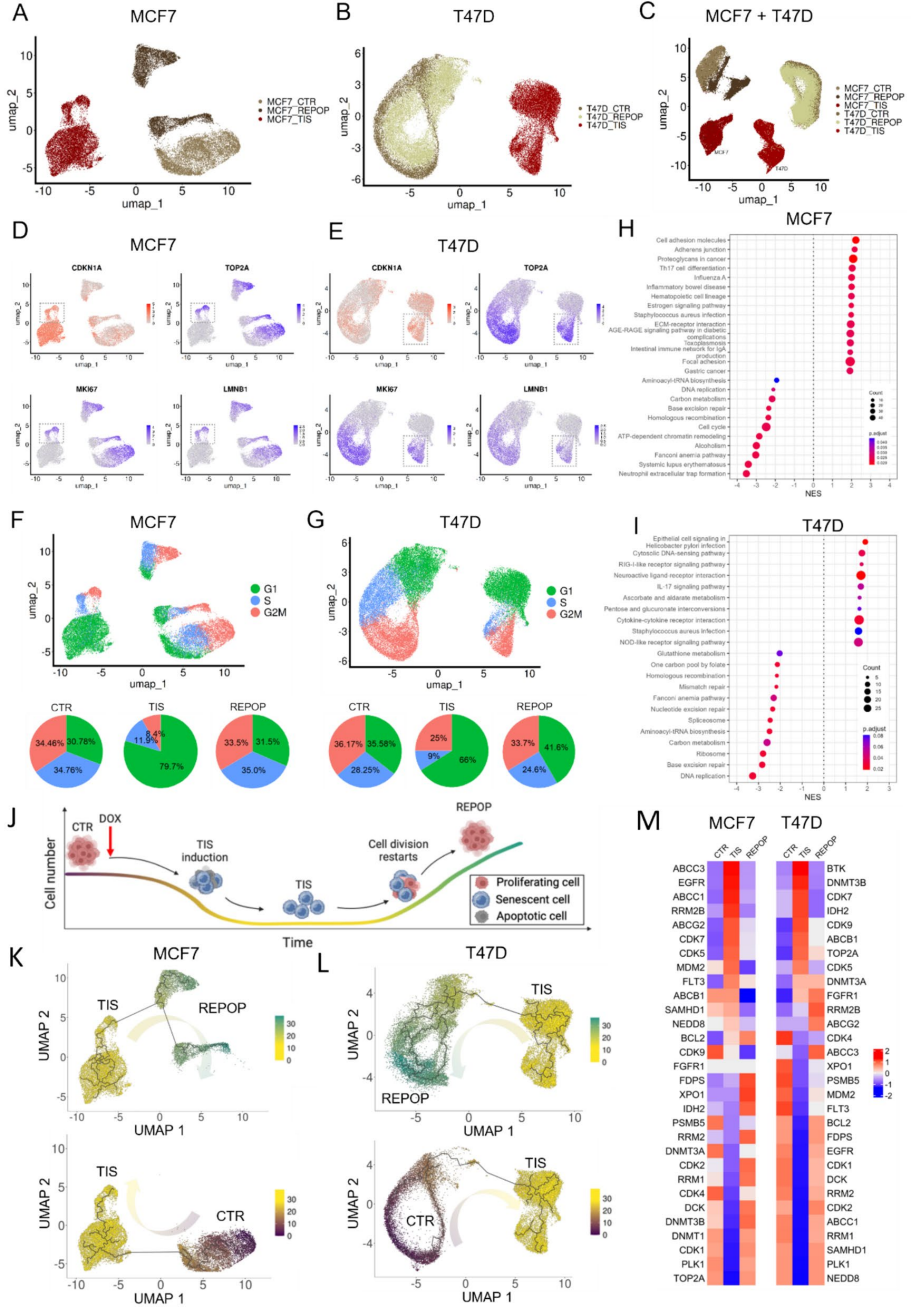
Therapy-induced senescence (TIS) cells that escape senescence drive repopulation after chemotherapy. **A** UMAP projection of single-cell transcriptomes from the MCF7 breast cancer cell line, illustrating distinct clustering of control (CTR, dark brown), therapy-induced senescent (TIS, scarlet), and repopulating (REPOP, light brown) cell populations. The separation of these clusters indicates transcriptionally distinct states, with TIS cells forming a well-defined cluster distinct from CTR and REPOP populations. The REPOP population shows a partial transcriptional shift toward CTR, reflecting its reversal from the TIS state. **B** UMAP projection of single-cell transcriptomes from the T47D breast cancer cell line, similarly showing distinct clustering of CTR (dark green), TIS (crimson), and REPOP (light green) cell populations. The clustering pattern resembles that observed in MCF7 cells, with a well-separated TIS population and REPOP cells positioned between TIS and CTR clusters, suggesting partial transcriptional reversion. **C** Integrated UMAP analysis of MCF7 and T47D cell lines, combining data from both models to highlight cell type-specific transcriptomic profiles. Control populations (MCF7 CTR in dark brown; T47D CTR in dark green) form distinct clusters, whereas TIS populations from both cell lines (scarlet for MCF7; crimson for T47D) also exhibit clear separation from their respective CTR counterparts. The integration further reveals that despite the transcriptional similarities in senescence-associated programs, MCF7 and T47D maintain cell-line-specific transcriptomic differences, as reflected in their segregated distributions. **D** Feature plots showing the expression of key senescence and proliferation-related markers in individual MCF7 cells. CDKN1A (p21) is upregulated in TIS cells (top left panel), confirming cell cycle arrest. Conversely, markers associated with proliferation, including LMNB1, TOP2A, and MKI67, are downregulated (top right and bottom panels), consistent with the senescent phenotype. The presence of scattered high-expressing cells within the TIS population suggests the existence of ‘escaper’ subpopulations that may retain some proliferative capacity. **E** Feature plots of CDKN1A, LMNB1, TOP2A, and MKI67 expression in T47D cells, showing a similar transcriptional profile to MCF7 cells. CDKN1A is significantly upregulated in TIS cells, while LMNB1, TOP2A, and MKI67 are markedly downregulated. As in MCF7, a fraction of TIS cells display non-uniform expression of these markers, indicating potential escape from the senescent state. **F** UMAP projection of MCF7 cells overlaid with cell cycle phase information (G1: green; S: blue; G2/M: red). Pie charts illustrate the proportional distribution of cells in each phase across the CTR, TIS, and REPOP populations. TIS cells predominantly reside in the G1 phase (79.7%), reflecting irreversible cell cycle arrest, whereas REPOP cells show an increased proportion of cycling cells, particularly in the G2/M phase, indicating their proliferative re-entry. **G** UMAP projection of T47D cells colored by cell cycle phases, with corresponding pie charts depicting phase distributions in CTR, TIS, and REPOP populations. TIS cells in T47D exhibit a similar G1 arrest phenotype as observed in MCF7, while REPOP cells regain a more balanced cell cycle distribution, mirroring their recovery from senescence. **H** Gene set enrichment analysis (GSEA) of differentially expressed genes in MCF7 TIS cells compared to CTR, highlighting significant enrichment of senescence-associated pathways (e.g., inflammatory response, DNA damage signaling) and suppression of proliferation-associated pathways. Normalized enrichment scores (NES) are shown, with positive values indicating upregulated pathways and negative values indicating suppressed pathways. **I** GSEA results for T47D TIS cells, demonstrating pathway-level alterations similar to those observed in MCF7, with enrichment of senescence-associated programs and suppression of cell cycle progression. **J** Schematic representation of the senescence induction and reversion process. Cells undergo therapy-induced senescence (TIS) following exposure to doxorubicin (DOX). Over time, a subset of TIS cells escape growth arrest and re-enter the cell cycle, forming the REPOP population. This process involves transcriptional reprogramming, with a balance between senescent, proliferating, and apoptotic fates. **K** UMAP trajectory analysis of MCF7 cells depicting the transition from CTR to TIS (bottom panel) and from TIS to REPOP (top panel). Cells are colored based on pseudotime, capturing the progressive shift in transcriptional states. TIS cells form a distinct branch, while REPOP cells demonstrate convergence toward CTR, reflecting their transcriptional plasticity. **L** UMAP trajectory analysis of T47D cells, analogous to MCF7. The bottom panel illustrates the transition from CTR to TIS, while the top panel depicts the transition from TIS to REPOP. The REPOP population becomes almost identical to the CTR cluster, exhibiting highly overlapping transcriptional profiles, even more profoundly than in MCF7. This supports a model of senescence escape and complete proliferative recovery. **M** Heatmap of gene expression changes in a curated set of genes associated with drug resistance, senescence regulation, and cell cycle control across CTR, TIS, and REPOP states in both MCF7 and T47D cell lines. Genes exhibit dynamic expression patterns, with key senescence markers upregulated in TIS and downregulated upon REPOP transition, while drug resistance-associated genes show variable trends between cell lines. This highlights the complex interplay between senescence, proliferation, and therapy resistance

To further characterize the transcriptional changes and study the dynamics of TIS, as well as the molecular basis of potential resistance and escape mechanisms, we performed single-cell RNA sequencing (scRNA-seq) of MCF7 and T47D breast cancer cell lines (Fig. 5A and B). To ensure the quality and usability of our scRNA-seq data, we first assessed the datasets by analyzing replicates and comparing differentially expressed genes from pseudo-bulk analysis to bulk RNA-seq. We observed minimal to no batch effect among the replicates and high concordance between bulk and pseudo-bulk comparisons, confirming the reliability of our scRNA-seq datasets (Figure S10C and D). As expected from our bulk RNA-seq analysis, unsupervised clustering followed by uniform manifold approximation and projection (UMAP) of single-cell gene expression profiles revealed a distinctly different transcriptional state for TIS cells compared to the CTR in both cell lines. In contrast, REPOP cells clustered closely with the CTR population and, in the case of T47D cells, were nearly identical to the original CTR population, underscoring the reversible and transient nature of the TIS state. Interestingly, the replicates of MCF7 REPOP samples exhibited substantial variability, even though they were collected at the same time post-treatment, indicating that the escape from senescence may occur with distinct and variable kinetics. Importantly, when the 316 shared genes, previously identified from the bulk RNA-seq (Fig. 4N), were projected onto single cell RNA sequencing results from MCF7 and T47D, the pattern specifically identified TIS cells (Figure S10E and F) confirming that the discovered gene set is TIS-specific. Integrated analysis and joint normalization of MCF7 and T47D cell lines highlighted both cell type-specific and senescence-related transcriptomic alterations (Fig. 5C). This analysis demonstrates that while the inherent gene expression profile of each cell type remains the most defining feature, the TIS cell state introduces a secondary, yet dominant and distinct, dimension. We observed no overlap between TIS and CTR populations on the different UMAPs and did not find batch effect among the replicates (Figure S11 A-D).

### Senescence factors arresting cell cycle measured by scRNA-seq

By visualizing the expression levels of the CDKN1A, a cell cycle inhibition and known senescence marker gene, in individual cells, we observed its significantly elevated expression in the TIS populations (Fig. 5D and E, top left panels). Along with the mostly undetectable expression of the nuclear lamina component LMNB1 gene (Fig. 5D and E, bottom right panels), another frequently used marker of senescence, these findings strongly indicate a robust cell cycle arrest in the TIS cell population, which is not observed in the CTR or REPOP cells. The expression of these genes showed a strong correlation with the previously observed protein levels (Fig. 1C). Detailed analysis of additional cell cycle markers, such as TOP2A and MKI67, further corroborates the presence of non-dividing cells within the senescent population (Fig. 5D and E, top right and bottom left panels). However, we identified a subpopulation of cells, termed'escapers,'which, while simultaneously expressing growth activators and suppressors, may re-enter the cell cycle and form the basis for repopulation, as indicated by the dotted-line rectangles in Fig. 5D and E. In alignment with this observation, we projected cell cycle-specific gene expression patterns onto all cells to calculate the ratios of the G1 (gap), S (synthesis), G2, and M (mitosis) cell cycle phases among the three experimental conditions (CTR, TIS, and REPOP) in both cell lines (Fig. 5F and G). In the CTR populations of both cell lines, we observed approximately equal ratios of cells in the G1, S, and G2/M phases. This distribution shifted dramatically towards the G1 phase in senescent cells, with 79.7% and 66% in MCF7 and T47D cell lines, respectively. This further supports the notion that senescent cells are in cell cycle arrest, potentially serving as a crucial mechanism for evading the effects of various drugs. After repopulation, the cell cycle balance was almost completely restored to resemble the CTR population.

### Single cell transcriptomics identifies activated and suppressed pathways related to TIS cell state

Single-cell transcriptomics has provided a detailed view of the molecular landscape in TIS cells, revealing specific alterations that may endow these cells with unique properties to survive or resist drug treatments. In the MCF7 cell line (Fig. 5H), three key DNA repair pathways — Homologous Recombination, Base Excision Repair and Fanconi Anemia Pathway — were significantly suppressed (Figure S12 A,C,E,G,I). This downregulation suggests a reduced capacity to maintain genomic stability, contributing to TIS cell survival despite DNA damage. ATP-dependent Chromatin Remodeling was also suppressed, limiting chromatin changes for DNA repair and gene regulation during senescence. Both Cell Cycle and DNA Replication pathways were suppressed, likely keeping TIS cells in growth arrest and enabling them to evade the cytotoxic effects of chemotherapeutics targeting dividing cells. Conversely, pathways like Estrogen Signaling, ECM-Receptor Interaction, and Cell Adhesion Molecules were activated, suggesting increased cellular signaling and adhesion, potentially altering the tumor microenvironment to support TIS cell survival.

In the T47D cell line (Fig. 5I), similar to MCF7 cells, all three previously mentioned DNA repair pathways, as well as Mismatch Repair and Nucleotide Excision Repair, were suppressed (Figure S12B,D,F,H,J). In addition to these pathways, Spliceosome and One-Carbon Pool by Folate pathways were also suppressed, indicating a broader disruption in cellular metabolism and mRNA processing, which may further stabilize the TIS state.

These findings underscore the complexity and adaptability of TIS cells, revealing reduced DNA repair capabilities and significant alterations in signaling pathways. This molecular reprogramming likely supports the maintenance of the TIS cell state and the resistance of the cells to a variety of therapeutic agents.

### Trajectory analysis reveals the reversible nature of TIS

To better understand cell state transitions and transcriptomic changes, we performed trajectory analysis on CTR, TIS and REPOP cells (Fig. 5J). Differential gene expression analysis was conducted on MCF7 and T47D (CTR vs. TIS). The overlapping genes from both comparisons were then cross-referenced with the trajectory analysis results. All 93 genes were found to be differentially expressed. This analysis confirmed that the CTR and REPOP stages are closely related in terms of their transcriptomic profiles (Fig. 5K and L). Furthermore, trajectory analysis indicated that release from cell cycle arrest is critical for the transition from TIS to REPOP. However, these trajectories could be heavily influenced by the expression of genes directly related to the cell cycle which could falsely separate TIS cells from the others. Therefore, the analysis was performed again with the exclusion of these genes, but the results were the same (Figure S13). These findings emphasize the reversible nature of TIS, highlighting the potential for cells to re-enter the cell cycle and proliferate once the arrest is lifted.

### Gene expression patterns cannot explain the drug resistance of TIS cells

By investigating the expression of the previously selected drug resistance genes we found no common mechanism that could explain the significantly increased drug tolerance of TIS cells from both cell lines (Fig. 5M). Similar to the results obtained from bulk RNA sequencing, there are genes that could seemingly play a role in drug resistance in one of the cell lines, but comparing their expression levels to the other line or REPOP cells disproves it. For example, TOP2A downregulation could be the reason why MCF7 cells are resistant to topoisomerase II inhibitors, however T47D cells are also resistant to topo II poisons, while overexpressing TOP2A. Conversely, decreased NEDD8 levels can cause pevonedistat resistance in T47D TIS cells, but MCF7 TIS cells are also insensitive to the drug while significantly overexpressing NEDD8 compared to CTR and REPOP cells. These findings, along with the bulk RNA-seq results, suggest that TIS cells exhibit an inherent drug resistant phenotype that is not directly linked to single genes or mechanisms.

### Targeting the top overexpressed genes in TIS cells revealed no exploitable sensitivity

**Fig. 6:**
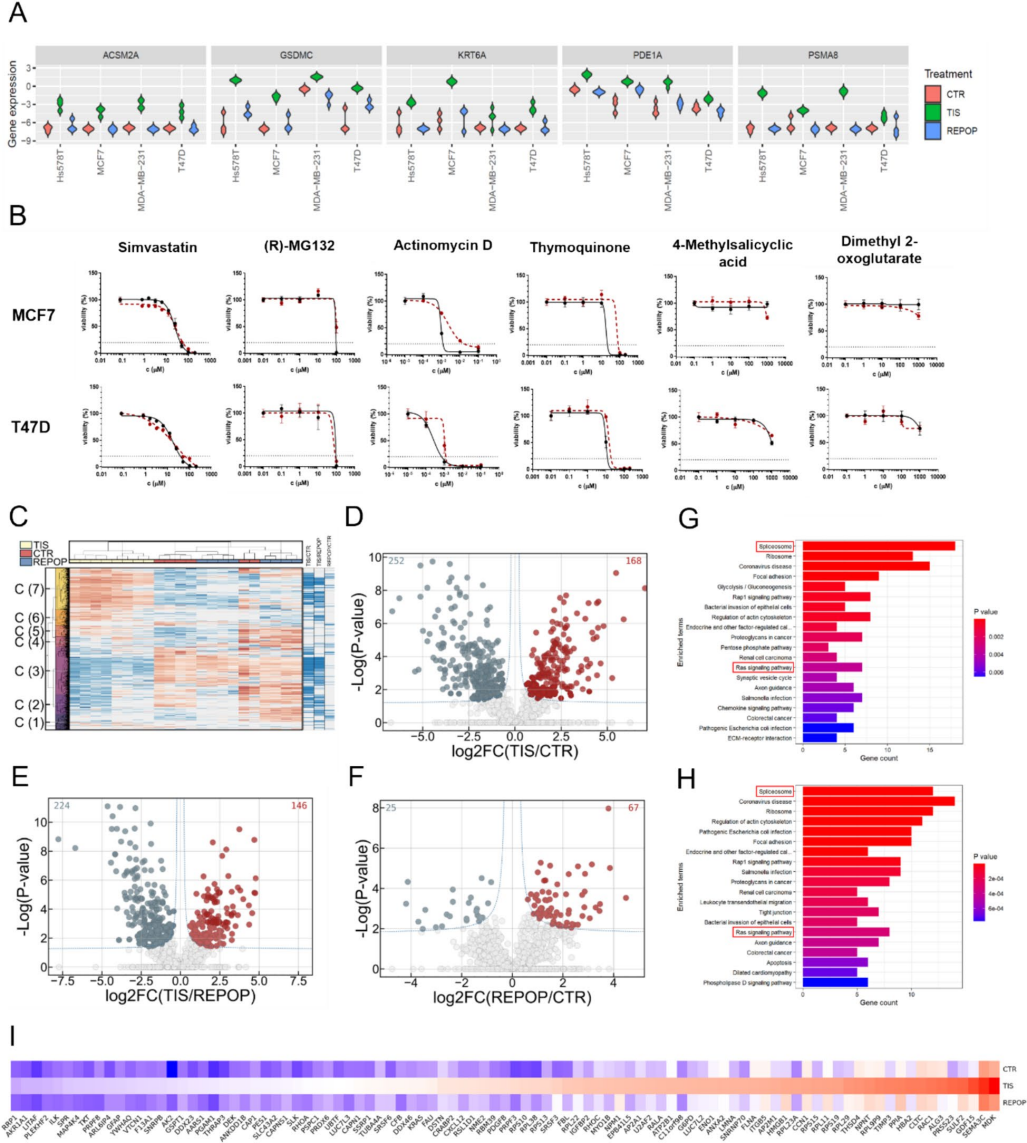
TIS cells exhibit a unique proteomic profile. **A** Expression levels of the top five proteins, for which inhibitors are available, in control (CTR, red), TIS (green), and repopulated (REPOP, blue) cells. **B** Sensitivity of CTR (black) and TIS (red) cells to six different inhibitors targeting the proteins identified in panel (**A**). **C** Hierarchical clustering analysis of Z-score normalized proteomic profiles from MCF7 CTR, TIS, and REPOP samples, demonstrating distinct clustering of each state. Proteins with significantly different expression levels (FDR < 0.05) in different comparisons are marked on the right. **D**, **E** Volcano plots illustrating protein expression changes during the CTR-to-TIS transition (**D**) and the TIS-to-REPOP transition (**E**). Significance limits (FDR < 0.05) are shown by dotted blue lines, significantly increased or decreased proteins are colored red or blue, respectively. Number of proteins with significant change are shown at the top corners. **F** Volcano plot showing protein expression differences between CTR and REPOP states. Significance limits (FDR < 0.05) are shown by dotted blue lines, significantly increased or decreased proteins are colored red or blue, respectively. Number of proteins with significant change are shown at the top corners. **G**, **H** Signaling pathway enrichment analysis of differentially expressed proteins, highlighting altered pathways in the TIS vs. CTR (G) and TIS vs. REPOP (H) comparisons. **I** Heatmap of proteins uniquely overexpressed in TIS cells, underscoring their distinct proteomic signature

Identifying compounds or strategies capable of selectively eliminating TIS cells represents a transformative step toward developing novel therapeutic options for cancer treatment. To achieve this, we first pinpointed the most overexpressed genes in TIS cells across all four cell lines using our bulk RNA-seq data. We then selected the top five genes for which commercially available inhibitors were accessible (Fig. 6A, Table S5). Previous studies have shown that statins effectively reduce Keratin 6A (KRT6A) gene promoter activity, leading to their use as a first-line treatment for pachyonychia congenita, a rare skin disorder linked to KRT6A [52]. Based on this, we treated CTR and senescent cells with simvastatin; however, no difference in sensitivity was observed (Fig. 6B). The proteasome 20S subunit alpha 8 (PSMA8), a proteasomal protein sensitive to MG132, a potent proteasome inhibitor, was also tested to determine if MG132 selectively targets senescent cells. However, the response was consistent in both CTR and senescent cells, similar to results with other proteasome-targeting drugs previously tested [53]. Additionally, although thymoquinone (TQ) has been reported to repress phosphodiesterase 1A (PDE1A) expression [54], our assay demonstrated that TQ induced comparable cytotoxicity in both CTR and senescent cells. We also investigated gasdermin C (GSDMC), which induces pyroptosis upon cleavage by caspase-8, as it was highly expressed in TIS cells. We tested the effects of dimethyl 2-oxoglutarate (DM-αKG) and actinomycin D, compounds known to elevate caspase-8 levels and trigger GSDMC cleavage [55, 56]. Regrettably, DM-αKG was non-toxic, while TIS cells exhibited resistance to actinomycin D. Lastly, we inhibited acyl-CoA synthetase medium chain family member 2A (ACSM2A) using 4-Methylsalicylic acid [57], but it showed no toxicity in either cell type.

Due to we based our treatment approach on mRNA expression, the protein levels of each target were measured (Figure S14). While no consistent upregulation of these was observed across all cell lines, certain cases exhibited increased expression upon TIS induction. Specifically, ACSM2A and GSDMC were overexpressed in MDA-MB-231, PSMA8 in MCF7, and PDE1A in both MCF7 and T47D cells. Despite these expression changes, none led to collateral sensitivity in TIS cells, reinforcing the hypothesis that TIS itself confers a protective advantage to cancer cells, independent of drug target protein overexpression.

Taken together, these findings reveal that despite targeting a spectrum of highly expressed senescence-associated genes with carefully selected inhibitors, none of the tested compounds demonstrated the selective cytotoxicity required to effectively eliminate senescent cells – highlighting the significant challenge of overcoming therapy-induced senescence in cancer treatment.

### Proteomics analysis of TIS cells highlights SASP and a plausible escape mechanism

Recognizing the limited potential of traditional therapeutic approaches, we advanced toward a novel strategy centered on immune and cell surface/extracellular targets. Using a specific biotin labeling technique to pull down membrane and extracellularly secreted proteins – developed by Langó et al. [29, 30] (Figure S15) – on MCF7 CTR, TIS, and REPOP cells, we aimed to identify TIS-specific proteome expression variations, uncovering new targets for selective inhibitors or immunotherapy. Clustering of 6, 8 and 8 biological replicates of CTR, TIS and REPOP samples, respectively, revealed that, while CTR and REPOP cells exhibit nearly identical proteomes, TIS cells display a distinct protein profile (Fig. 6C). Specifically, 420 proteins were differentially expressed by TIS cells compared to CTR, and 370 compared to REPOP samples, whereas only 92 proteins varied between CTR and REPOP cells (Fig. 6D, E and F). From these distinct protein sets, two pathways – spliceosome and RAS signaling – emerged as recurrent features, aligning with findings from our transcriptome analysis described above (Fig. 6G,H). Although the role of spliceosome in cellular senescence [58–60] and SASP [61] is a rapidly developing area of research, the large-scale presence of splicing proteins on the cell surface and/or secreted form is unprecedented. Interestingly, 85 out of the 95 differently expressed proteins were earlier detected in extracellular vesicles secreted by senescent tumor cells [62]. Furthermore, RAS overexpression has previously been associated with senescence, primarily as an oncogenic signal triggering [63], rather than as part of TIS dynamics. KRAS protein was overexpressed in MCF7 TIS cells (Fig. 6I), despite its typically marginal expression in breast cancer cells and tumors [64]. This finding aligns with our bulk RNA-seq results, which revealed substantial changes in both the up- and downregulated gene sets associated with KRAS expression (Fig. 4K, L). Unexpectedly, TIS cells did not exhibit elevated KRAS mRNA expression compared to CTR and REPOP cells (Figure S16A), nor did they show increased KRAS protein levels (Figure S16B). It was hypothesized that the differential regulation of KRAS-related genes could still result from an oncogenic mutation induced by high-dose DOX treatment. However, deep sequencing of KRAS confirmed that both CTR and TIS cells in both cell lines retained the non-mutated wild-type gene (Figure S16 C,D). The lack of KRAS involvement in breast cancer cells was further supported by treating CTR and TIS cells with RMC-6236, a pan-KRAS inhibitor, which did not elicit significant sensitivity in either condition (Figure S16E). These findings strongly suggest that KRAS pathway activation in TIS cells occurs through a non-canonical mechanism.

The analysis of proteomics results further indicated extensive secretion of SASP-related proteins (Fig. 6I). Of the 95 proteins overexpressed exclusively in TIS cells, 41 were associated with senescence and 17 with alternative splicing and the spliceosome (Table S6 A and B). Furthermore, we found that FBL [65], NME2 [66], FLNA [67], ENO1 [68, 69] and ANXA2 [70, 71] were described in a recently identified set of SASP proteins, found in irradiated, RAS-transformed and atazanavir treated senescent fibroblasts, further strengthening the similarity between TIS in cancer cells and TIS and OIS in healthy cells [65]. These findings underscore the distinct molecular landscape of TIS cells, with KRAS overexpression, SASP protein secretion, and unique associations with senescence and splicing pathways, highlighting potential new avenues for therapeutic targeting.

We also observed the translocation of high mobility group box 1 (HMGB1) protein from the nucleus to the plasma membrane – a recently discovered Damage-associated molecular pattern (DAMP) signal originating from senescent cells [72–76] as well as the secretion of growth differentiation factor 15 (GDF15) a newly recognized marker of senescence and aging [65, 77]. Validation of the proteomic data was performed using individual peptide sequence analysis (Figure S17 A,B).

Interestingly, partially overlapping with the senescence markers, a group of proteins related to the spliceosome were also identified in our assay (Table S6B). Each of these proteins either plays a role in constitutive and/or alternative splicing or forms part of the spliceosome, indicating a significant involvement of RNA splicing in establishing and maintaining the TIS phenotype.

The additional 52 proteins had not previously been identified as contributors to senescence, suggesting that some may play roles in the maintenance or regulation of TIS, or even serve as biomarkers of this phenotype. The mRNA expression levels of the 95 protein encoding genes were investigated in bulk and scRNA-seq results (Figure S18 A and B). Notably, ribosomal proteins were enriched within this group, supporting prior findings that link senescence with ribosome biogenesis defects [78].

Consistent with our RNA-seq results, no single resistance mechanism or mechanism group was identified that could fully account for the heightened drug tolerance observed in TIS cells. In addition to transcriptional alterations, therapy-induced senescence may be accompanied by widespread chromatin remodeling, which could contribute to its persistence and heterogeneity.

## Discussion

Therapy-induced senescence (TIS) has recently emerged as a promising strategy in cancer treatment [79, 23]. The concept of inducing cytostasis in cancer cells, rather than eliminating them, has gained attention for its potential to expand therapeutic options across malignancies by shifting away from solely relying on high doses of toxic drugs and/or radiation — which carry severe side effects and the risk of drug resistance — TIS offers an approach to permanently arrest cell proliferation in tumors [22]. However, our findings suggest that TIS may function less as an ideal therapeutic endpoint and more as a robust resistance mechanism, underscoring the need to reconsider its implications in treatment strategies.

To effectively compare studies and findings, a clear definition of TIS in cancer cells is essential. Senescence is widely regarded as an irreversible growth arrest in both healthy and malignant cells [80]. However, recent studies suggest that some subpopulations of senescent cancer cells can evade this arrest, regaining proliferative capacity through escape or reversion mechanisms [24, 81].

The primary challenges in TIS-studies and defining senescence in cancer cells is the absence of specific markers [82, 38] and the diversity of treatment protocols, encompassing a range of drugs and treatment duration to induce senescence.

Senescence is generally considered irreversible only if the inducing stimulus persists for at least 4 days [83, 84]; cancer cells treated for less than 96 h can readily escape TIS. For example, early studies showed that H1299 lung cancer cells could evade TIS after 1–3 days of camptothecin treatment, with escape observed approximately 20–25 days post-treatment — similar to our observations. Notably, p16- and p53-negative H1299 cells were used, as p53 and p16 are known to limit senescence reversibility [24]. In another model, a 2 h DOX treatment induced TIS in MCF7 cells, and a small subset escaped long-term arrest despite wild-type p53 expression, forming clonal outgrowths within 2–3 weeks [85]. More recent studies similarly observed TIS escape in various cell lines, but proliferation resumed significantly earlier, 5–7 days after treatment removal, with treatment durations not exceeding 24 h [27, 86]. Saleh et al. conducted a series of studies on TIS escape of H460, A549, and HCT116 BTG1-RFP cells, using various drugs. In most cases, cells regained proliferative capacity approximately 7 days after treatment [86, 27].

Tóth et al. investigated TIS induction in MCF7 and MDA-MB-231 cell lines using bromodeoxyuridine, gemcitabine, and palbociclib. Remarkably, despite drug treatments lasting up to 1–2 weeks, TIS-escaped cells began appearing within 7 days upon finishing the treatment [87]. The early escape from TIS raises the question about whether fully developed TIS was achieved or if cells entered a transient growth arrest [88] or a reversible phase of senescence due to short-term or lower-dose treatments. A study published in 2023 systematically examined the duration dependency of TIS induction, demonstrating that MCF7 cells commit irreversibly to TIS only after continuous MEK and CDK4/6 inhibition for over 4 days [89]. Our experimental design ensured the senescent phenotype was established by applying high-dose DOX for 120 h, with surviving cells analyzed one week post treatment. The observed repopulation time (> 30 days) closely mirrored relapse times in a clinically relevant mouse model of triple negative breast cancer following complete response to DOX [90, 91].

Using multiple markers and features to confirm the presence of the senescent phenotype is not only recommended but essential due to the complexity of senescence. In our system, both the initially selected markers and additional findings supported our hypothesis that the surviving cells were in TIS. For example, the TIS cell surfaceome revealed extensive secretion of different factors, including numerous SASP proteins, HMGB1 translocation, and ribosomal elements. Alongside with the established senescence markers like CDKN1A and LMNB1, our transcriptome analysis identified a distinctive expression pattern, comprising genes upregulated across all cell lines exclusively during TIS, which may serve as a specific TIS signature in breast cancer. This signature includes genes known for their roles in cellular senescence regulation such as matrix metallopeptidase 12 (MMP12) [92–94], cytochrome P450 family 1 subfamily A member 1 (CYP1A1) [95, 96], baculoviral IAP repeat containing 3 (BIRC3) [97–99], collagen type IV alpha 3 chain (COL4A3) [100], and neurotrophic receptor tyrosine kinase 2 (NTRK2) [101], as well as genes associated with aging, like C–C motif chemokine ligand 26 (CCL26) [102], glutamate ionotropic receptor delta type subunit 2 (GRID2) [103], carbonic anhydrase 10 (CA10) [104, 105], and C-type lectin domain family 12 member A (CLEC12A) [106]. Interestingly, many genes in this set are not previously linked to senescence, offering a novel pool of potential TIS markers. Additionally, genes with functions that protect against senescence, such as reelin (RELN) [107–109], were also present, potentially indicating a molecular toolkit supporting cell survival and repopulation.

Our surfaceome analysis revealed a substantial secretion of spliceosome-associated proteins. Although spliceosome involvement in cellular senescence [58–60] and the senescence-associated secretory phenotype (SASP) [61] is an emerging field, no direct evidence currently links spliceosome components to intercellular communication during cellular senescence. However, recent studies suggest a potential role for spliceosome components in mediating intercellular communication, particularly under stress conditions in cancer. Under cellular stress induced by chemotherapeutic agents, γ-irradiation, or hypoxia, spliceosome elements-including heterogeneous nuclear ribonucleoproteins (hnRNPs), members of the family of serine/arginine (SR)-rich proteins, and small nuclear RNAs (snRNAs) are known to translocate from the nucleus to the cytoplasm and aggregate in stress granules. This redistribution, notably of overexpressed factors like serine/arginine-rich splicing factors 1 and 3 (SRSF1, SRSF3), has been observed under various stressors. Furthermore, spliceosome components can be secreted in extracellular vesicles from stressed tumor cells. When taken up by neighboring tumor cells, these splicing factors may play a pivotal role in modulating molecular processes within the recipient cells, potentially enhancing their resilience to chemotherapy [110]. Our findings suggest that the secretion and subsequent internalization of spliceosome components may confer adaptive advantages under stress conditions within the context of cellular senescence.

When cells enter senescence, they inherently become more resistant to apoptosis [111], which has led to the use of antiapoptotic inhibitors as senolytics. However, our findings suggest that targeting these pathways in TIS cancer cells may be unproductive. Given that TIS cells are non-proliferative and supposedly overexpress antiapoptotic proteins from the Bcl-2 family, one would expect them to show higher tolerance to most anticancer agents – yet, this is not the case. Apoptosis resistance in senescent cells was first observed in healthy fibroblasts under serum deprivation [112], which prompted the application of Bcl-2 inhibitors as senolytics against non-malignant senescent cells [113]. Although some reports suggest that antiapoptotic inhibitors might target senescent cancer cells, our experiments with venetoclax, navitoclax, ABT-737 and A1331852 yielded mixed results. Venetoclax showed no selectivity for TIS cells, while the other three compounds displayed some TIS-specific cytotoxicity; however, the outcomes in long-term assays were inconsistent (Fig. 3). Strikingly, these results appeared unrelated to Bcl-2 or Bcl-XL expression, as both proteins were already overexpressed in the parental cell lines (Figure S2). This raises the question of whether antiapoptotic inhibitors truly act as senolytics by targeting Bcl-2/XL in TIS cancer cells or through an alternative, yet unidentified mechanism.

Although an apoptosis-resistant phenotype would theoretically render TIS cells resistant to a broad spectrum of drugs [114], our findings did not support this expectation. For example, while HDAC inhibition has been proposed as a senolytic approach in various cancer types [115], it was equally toxic to both CTR and TIS cells in our screening (Fig. 2) and is known to induce cell death via apoptosis [116]. Similarly, apoptosis-inducing proteasome inhibitors [117] also proved toxic to both CTR and TIS cells in our experiments, challenging the notion that TIS cells are resistant to apoptosis. Tyrosine kinase inhibitors, which also kill cancer cells by inducing apoptosis [118], showed no selective resistance in TIS cells either. These findings strongly argue against apoptosis resistance in TIS cancer cells.

The hallmark of senescent cells is their complete lack of proliferation [38]. It is widely accepted that slowly or non-proliferating cells tolerate significantly higher concentrations of chemotherapeutics compared to rapidly dividing populations [119]; thus, the drug resistance profile of TIS cells could theoretically be attributed to their long-term exit from the cell cycle. However, several findings challenge this interpretation. Drugs that typically rely on cell proliferation, such as AT7519 [120], ibrutinib [121], masitinib, sorafenib and sunitinib [122] should theoretically have no effect on non-dividing cells, yet they effectively killed TIS cells. Conversely, compounds without cell cycle specificity or with documented efficacy against quiescent cells – such as carfilzomib [123, 124], homoharringtonine [125] and melphalan [126], failed to harm TIS cells. This unexpected combination of effects underscores the unique chemoprotection provided by TIS and draws a parallel with drug-tolerant persister cells, as both arise following high drug exposure without involving known resistance mechanisms [127].

One of the most intriguing findings in our study is the recurring pattern of immune-modulatory gene and protein expression. While some reports suggest that senescent cells are immunogenic and cleared by the immune system [128, 129], others indicate that these cells evade the immune response [130, 131]. Our results demonstrate that TIS cells can significantly influence immune cell behavior through secreted factors and altered gene expression. Notably, we observed suppression of the Cytokine-Cytokine Receptor Interaction pathway, which is essential for immune cell communication and response. This downregulation may enable TIS cells to evade immune surveillance, reducing their chances of immune clearance. Combined with the activation of Neuroactive Ligand-Receptor Interaction pathways, these changes suggest that TIS cells undergo substantial alterations in environmental interactions, potentially creating a supportive niche for their long-term survival – even in a controlled culture setting where tumor microenvironmental factors are absent, but would otherwise be crucial. The activation of the Cytosolic DNA-Sensing Pathway, along with the Helicobacter pylori and Staphylococcus aureus Infection pathways, underscores an altered immune response in TIS cells. These pathways may represent adaptive mechanisms through which TIS cells manage chronic inflammatory signals or potential bacterial challenges, aiding their survival in a hostile microenvironment. Certain genes within these pathways are known to suppress or attenuate T cell responses. For instance, TIS cells in all tested cell lines overexpressed galectin 9 (LGALS9), a secreted galectin, recently shown to induce Gal-9/TIM-3 mediated apoptosis in T cells, thereby limiting their expansion and essentially acting as an immune inhibitor [132, 133]. C-X-C motif chemokine ligand 12 (CXCL12) is a cytokine which, if secreted in high concentration by senescent cells in the tumor microenvironment (TME), can create a “cytokine shield” that inhibits CD8+ T cell infiltration by downregulating C-X-C chemokine receptor type 4 (CXCR4) and impairing directional migration [134]. Triggering receptor expressed on myeloid cells 1 (TREM1) was found to suppress the antitumor immune response, and its inhibition significantly delays melanoma growth. TREM1 was also universally overexpressed in TIS cells across all four cell lines [135]. Metastasis-associated in colon cancer 1 (MACC1), an established metastasis regulator and marker, facilitates immune evasion in malignancies through various processes, primarily by regulating the expression of immune cell-modifying surface molecules [136]. The presence of these factors alone suggests a profound alteration of the local immune landscape and response, but TIS cells overexpress not only these, but many additional modulators, underscoring the complexity of targeting these cells through immune mechanisms.

Modulated immune reactivity may be an even more important consequence of TIS as it seems at first glance. REPOP cells are highly similar to the CTR lines in terms of drug tolerance and gene expression patterns, as shown by both the bulk and single cell sequencing results. However, comparing parental and repopulating cells and identifying the remaining gene expression changes may reveal mechanisms which enable repopulating cells to escape TIS with a higher efficiency upon repeated treatment. By comparing the transcriptomic data of CTR and REPOP cells, we found a signature of 23 genes, that were overexpressed in the malignant cells during TIS and remained highly expressed in the REPOP cells. This gene set contained several members of the IFIT (Interferon Induced proteins with Tetratricopeptide repeats) and the OAS (2-5A synthetase) family, which are primarily involved in the defense against viral infections. However, recent results indicate both protein family’s involvement in other pathologies, like cancer [137]. Several genes among the identified signature have already been connected to cancers or cellular senescence. Importantly, specific representatives of the residual signature genes (ISG15, IFI27, IFI6, RSAD2) appear to aid cancer cells in evading immunosurveillance and inflammatory cells by inhibiting apoptotic processes [138]. IFIT1, which was significantly overexpressed in all four malignant TIS cell types, has been shown to be significantly upregulated in residual breast cancer tumors [139]. It was also shown to be part of an IFN-related DNA damage resistance signature in breast cancer, together with ISG15, another gene in the signature [140]. IFIT3 was part of a SASP-related prognostic signature to assess therapeutic effect and prognosis in AML [141], indicating its role in the regulation of cellular senescence. Its high expression level was connected to increased resistance of different therapeutics in oral squamous cell carcinoma [142] and was identified as a hub gene in triple negative breast cancer [143]. IFITM1 was found to be overexpressed in SCLC tumors and was significantly increased in cisplatin-resistant SCLC tissues [144]. IFITM1 inhibition was also suggested to enhance the sensitivity to tamoxifen in ER-positive breast cancer cells [145]. OAS1 overexpression influenced survival and immune cell infiltration in patients with lung adenocarcinoma [146] and OAS2 was shown to participate in a sustained senescence response of human primary melanocytes upon repeated UVB exposure [147]. Genes implicated in the modulation of tumor microenvironment and immunomodulation include CLIC5 [148], RSAD2 [149] and OASL [150]. Collectively, this shows that TIS initiates long-term changes in malignant cells that remain even after returning to the proliferating state. These residual gene expression changes may facilitate immune evasion and anti-apoptotic processes, rendering the repopulating cells more resistant to future anti-cancer treatments.

Afifi et al. recently proposed that MCF7 cells could escape TIS through sustained overexpression of the MYC oncogene, demonstrating that MYC degradation makes senescence irreversible [89]. Although we did not observe MYC upregulation in TIS cells across any of the cell lines (data not shown), the hypothesis that a proto-oncogenic signal or pathway could compromise the irreversibility of senescence and enable certain cells to evade TIS warranted further investigation. As non-canonical KRAS pathway activation was observed in TIS cells throughout our study, from bulk RNA-seq data to surfaceome analysis, it could be an escape mechanism similar to MYC overexpression. Seemingly, KRAS protein itself is not required in TIS cells, but its pathway remains essential. The KRAS signaling pathway is well known for promoting cancer cell survival and immune evasion in tumors, and some studies suggest that it may also support repopulation under certain conditions. For example, KRAS overexpressing hematopoietic stem cells are less prone to irradiation-mediated damage and repopulate quicker after treatment, leading to accelerated hematologic recovery in a mouse model due to increased Erk1/2 phosphorylation and cyclin-dependent kinase 4 and 6 (Cdk4/6) activation [151]. The amplification of KRAS also renders lung cancer cells resistant to crizotinib, a MET inhibitor, by hypersensitizing them to ligand stimulation [152]. Since we also observed similar changes in TIS cells, we hypothesize that non-canonically activated KRAS pathway could act as a “doorstop” for senescence, inhibiting full commitment to irreversible TIS.

## Conclusion

In summary, our study redefines therapy-induced senescence (TIS) in cancer treatment by uncovering its complex adaptive mechanisms and resistance traits. Although TIS has emerged as a promising strategy to halt tumor progression, our findings suggest it functions more as a dynamic resistance state than a permanent growth arrest. Transcriptomic analyses revealed extensive shifts in gene expression within TIS cells, yet these changes do not reveal any definitive resistance mechanism based on current knowledge, underscoring the unique, elusive nature of TIS resistance.

The pronounced shift in the transcriptome profile within TIS cells suggests the potential involvement of epigenetic modulation or reprogramming in TIS formation and maintenance. Additionally, disparities between mRNA and protein levels imply that post-translational modifications (PTMs) may play a critical role in establishing and stabilizing senescence. Our data also show that TIS cells suppress immune signaling pathways, such as the Cytokine-Cytokine Receptor Interaction pathway, while activating immune-evasive and survival pathways, including increased secretion of galectin 9, CXCL12, and TREM1. These immune-modulatory factors likely help TIS cells evade immune clearance, fostering their long-term persistence.

These findings establish TIS not only as a novel mechanism of drug resistance but also as a formidable immune barrier. Growing evidence suggests that chemotherapy’s primary contribution to tumor reduction extends beyond direct cytotoxic effects, as it also enhances tumor immunogenicity, facilitating immune-mediated clearance. However, our results demonstrate that TIS cells actively evade immune elimination through a repertoire of secreted and surface-expressed factors, effectively shielding themselves from immune attack. Notably, these same immune evasion mechanisms may constitute exploitable therapeutic vulnerabilities. Targeting these pathways could prolong relapse-free survival and improve overall patient outcomes by enabling the immune system to eliminate TIS cells post-chemotherapy, thereby increasing the likelihood of durable remission and potentially achieving a cure.

These findings collectively highlight the limitations of TIS as a therapeutic endpoint. Rather than serving as a stable state, TIS equips cancer cells with adaptive mechanisms that complicate treatment. The potential involvement of epigenetic reprogramming and PTMs in TIS opens new avenues for investigation, suggesting that interventions targeting protein-level modifications or epigenetic alterations may be crucial for overcoming TIS-driven resistance. Future studies integrating epigenomic profiling, chromatin activity analysis, and transcription factor mapping will be essential to fully elucidate the regulatory landscape of TIS and its potential vulnerabilities. Understanding how TIS transitions to an active resistance mechanism could offer promising strategies for developing targeted treatments capable of dismantling this evasive phenotype.

## Supplementary Information

Supplementary Material 1

Supplementary Material 2

## Data Availability

The scRNA-seq and bulk RNA-seq datasets generated from this study are available in the NCBI GEO database under the accession numbers GSE280381 and GSE287953, respectively. Our analysis code has been uploaded to GitHub (https://github.com/lovricsa/TIS). The mass spectrometry proteomics data from this study were deposited in the ProteomeXchange Consortium with the dataset identifier PXD058028 and detailed results of quantitative analysis are available at DOI: 10.6084/m9.figshare.28595498. Any additional information required to reanalyze the data reported in this paper is available from the lead contact upon request.
