# Supplementary material for: Therapy-induced senescence is a transient drug resistance mechanism in breast cancer
Source: Mol Cancer. 2025 May 1;24:128. doi: 10.1186/s12943-025-02310-0 (PMC12044945; doi:10.1186/s12943-025-02310-0)
Supplement: Supplementary file 1 — Supplementary Material 1 [file 12943_2025_2310_MOESM1_ESM.pdf]

Supplemental Information

Therapy-induced senescence is a transient drug resistance mechanism in breast cancer

Eszter Bajtai, Csaba Kiss, Éva Bakos, Tamás Langó, Anna Lovrics, Éva Schád, Viktória Tisza, Károly Hegedűs, Péter Fürjes, Zoltán Szabó, Gábor E. Tusnády, Gergely Szakács, Ágnes Tantos, Sándor Spisák, József Tóvári, András Füredi

Table of contents

Supplementary Figure S1……………………………………………………………...2

Supplementary Figure S2……………………………………………………………...4

Supplementary Figure S3……………………………………………………………...6

Supplementary Figure S4……………………………………………………………...8

Supplementary Figure S5…………………………………………………………….....12

Supplementary Figure S6……………………………………………………………14

Supplementary Figure S7……………………………………………………………17

Supplementary Figure S8……………………………………………………………19

Supplementary Figure S9……………………………………………………………21

Supplementary Figure S10……………………………………………..……………23

Supplementary Figure S11……………………………………………..……………25

Supplementary Figure S12……………………………………………..……………27

Supplementary Figure S13……………………………………………..……………31

Supplementary Figure S14……………………………………………..……………33

Supplementary Figure S15……………………………………………..……………35

Supplementary Figure S16……………………………………………..……………37

Supplementary Figure S17……………………………………………..……………39

Supplementary Figure S18……………………………………………..……………41

Supplementary Table S1…………………………………………………………..….43

Supplementary Table S2…………………………………………………………..….44

Supplementary Table S3……………………………………………………………45

Supplementary Table S4…………………………………………………………….….46
Supplementary Table S5…………………………………………………………..…47
Supplementary Table S6…………………………………………………………..…48
REAGENT or RESOURCE…………………………………………………...………49

A

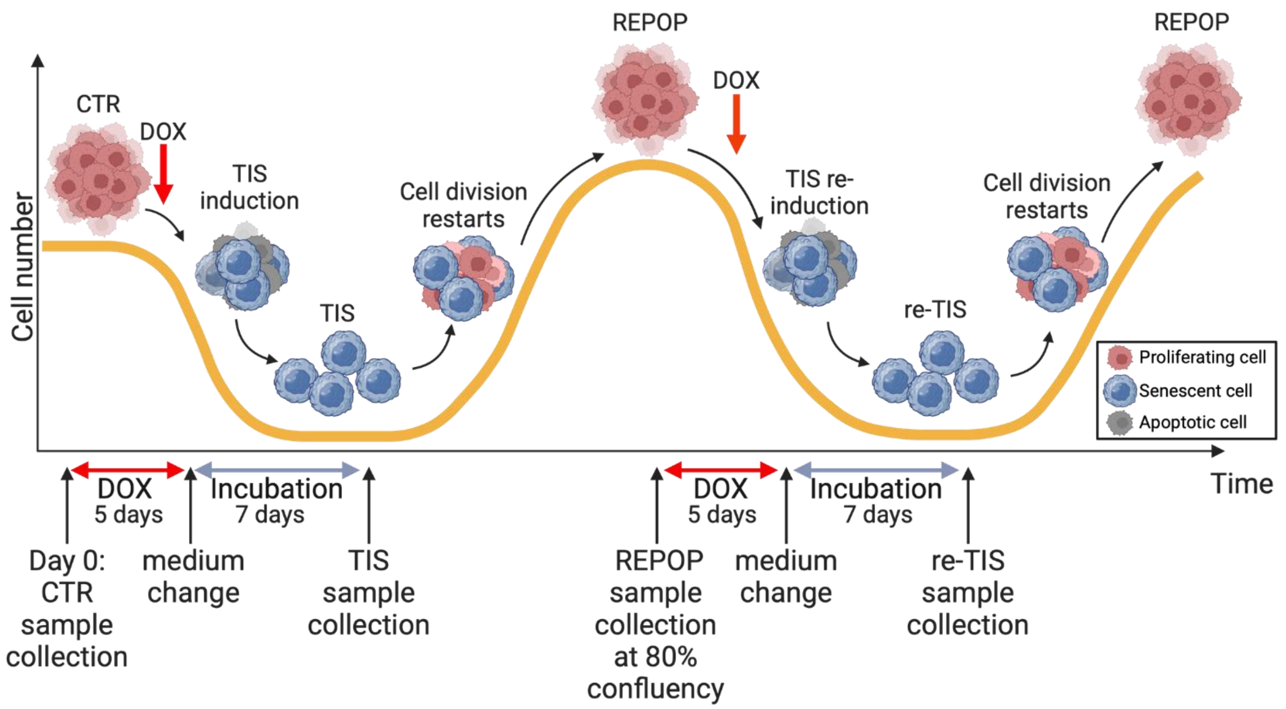

B

Experiment	CTR	TIS	REPOP	re-TIS
Bulk RNA Seq	✓	✓	✓	✗
scRNA Seq	✓	✓	✓	✗
Proteomics	✓	✓	✓	✗
Drug resistance	✓	✓	✓	✓
Cytokine expression	✓	✓	✗	✗

C

	Inducing stimulus persists >4 days	Changes in morphology and size	Canonical molecular marker positive	Reversible	Fading of markers	Escape in <10 days	Escape in >20 days
Transient growth arrest	no	no	yes (dim)	yes	yes	yes	no
Irreversible senescence	yes	yes	yes (strong)	no	no	no	no
Therapy-induced senescence	yes	yes	yes (strong)	yes	yes	no	yes

Supplementary Figure S1. Therapy-induced senescence, repopulation, and reinduction dynamics in breast cancer cells and the corresponding experimental analyses.

(A) Schematic representation of the therapy-induced senescence (TIS) cycle, repopulation (REPOP), and reinduction of senescence (re-TIS) in breast cancer cells. Doxorubicin (DOX) treatment induces TIS in proliferating cells, leading to growth arrest. After an extended incubation period, a fraction of TIS cells escape senescence and resume proliferation (REPOP). These REPOP cells can undergo TIS reinduction upon a second DOX treatment, demonstrating that the senescent phenotype is reversible. Sample collection points for control (CTR), TIS, REPOP, and re-TIS conditions are indicated along the timeline.

(B) Summary of experimental analyses performed on each cell population. Bulk RNA sequencing, single-cell RNA sequencing (scRNA-seq), proteomics, drug resistance assays, and cytokine expression profiling were conducted on CTR, TIS, and REPOP cells. However, re-TIS cells were excluded from RNA sequencing and proteomic analyses. Green check marks indicate completed experiments, while red crosses denote analyses that were not performed.

(*C*) A set of criteria was used to characterize TIS and differentiate it from transient growth arrest and irreversible senescence.

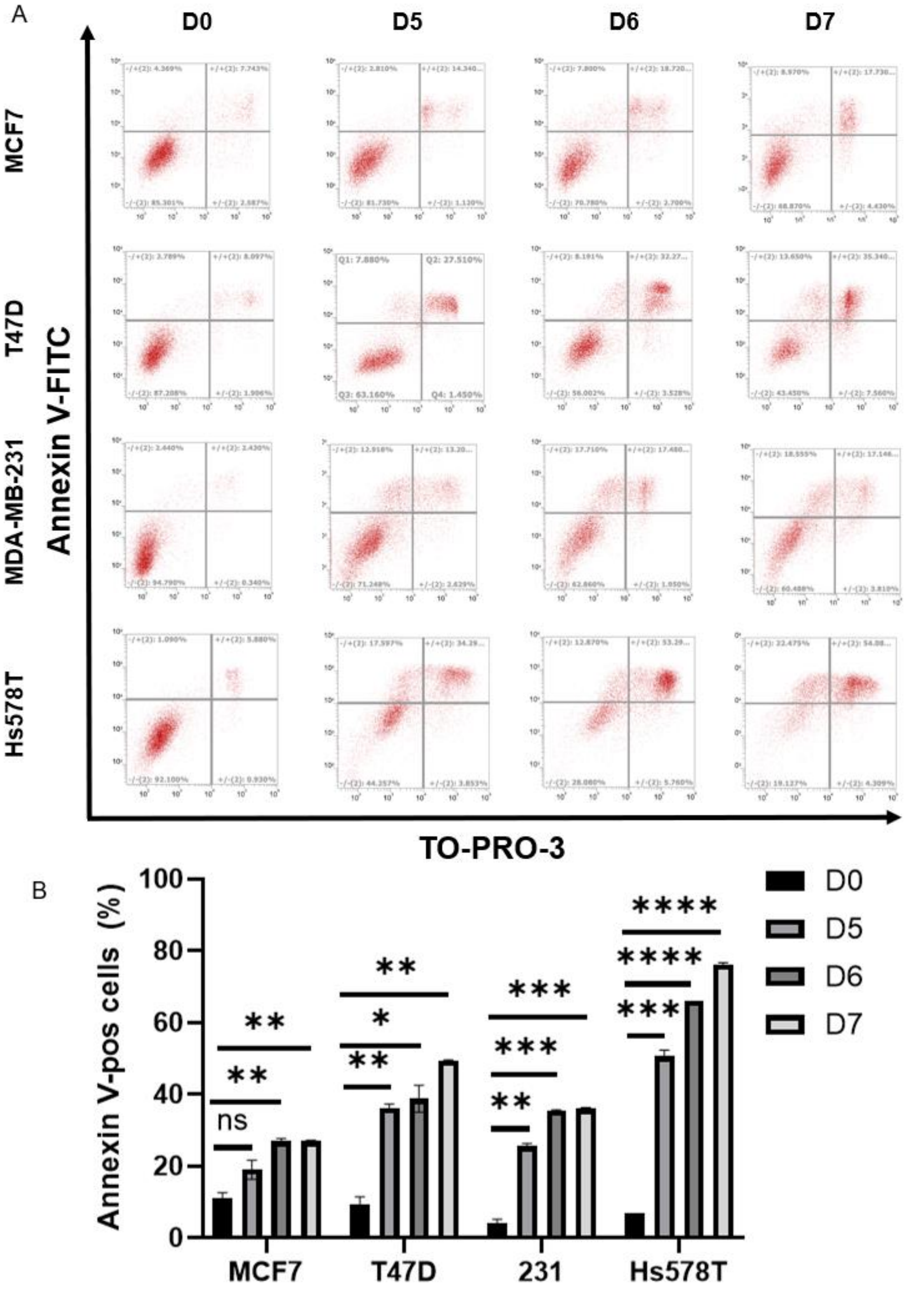
A
D0
D5
D6
D7
MCF7
T47D
MDA-MB-231
Hs578T
Annexin V-FITC
TO-PRO-3
B
Annexin V-pos cells (%)
100
80
60
40
20
0
D0
D5
D6
D7
ns
**
*
***
****
MCF7
T47D
231
Hs578T

Supplementary Figure S2. Flow cytometry assessment of apoptosis in four breast cancer cell line during TIS-induction using Annexin V-FITC staining.

(A) Flow cytometry analysis of apoptosis induction in breast cancer cell lines (MCF7, T47D, MDA-MB-231, and Hs578T) at the beginning of the experiment (Day 0 = D0) and following DOX treatment (D5, D6, D7). Annexin V-FITC and TO-PRO-3 staining were used to distinguish early apoptotic (Annexin V^+/TO-PRO-3^-, upper left quadrant), late apoptotic/necrotic (Annexin V^+/TO-PRO-3^+, upper right quadrant), and viable (Annexin V^-/TO-PRO-3^-, lower left quadrant) cell populations. Apoptotic cell fractions increased in all cell lines upon DOX treatment (D5–D7), with variations in sensitivity across different lines.

(B) Quantification of summarized Annexin V-positive cells at different time points (D0, D5, D6, and D7) across the four breast cancer cell lines. Apoptotic cell percentages significantly increased following DOX treatment, with Hs578T cells displaying the highest levels of apoptosis. Statistical significance was determined using one-way ANOVA; *$p < 0.05$, **$p < 0.01$, ***$p < 0.001$, ****$p < 0.0001$, ns = not significant. Error bars represent mean ± SD.

Apoptosis assay

Cells were seeded in 6-well plates at a density of 2×10^5 cells per well and incubated overnight under standard culture conditions. The following day, cells were treated with doxorubicin at concentrations 120 nM (MCF-7), 70 nM (T47D), 150 nM (MDA-MB-231), or 200 nM (Hs578T). On days 5, 6, and 7, both adherent and floating cells were collected by trypsinization, followed by centrifugation. Cells were washed twice with phosphate-buffered saline (PBS, pH 7.4), centrifuged, and resuspended in 100 μL of Annexin V binding buffer. 5 μL Annexin V-FITC (AD10, DOJINDO, Japan) and 1 μL TO-PRO-3 (R37170, Invitrogen, Waltham, MA, USA) were added to the cell suspension, followed by incubation in the dark for 15 minutes at room temperature. Subsequently, 400 μL of Annexin V binding buffer was added, and apoptotic cells were analyzed using an Attune™ NxT flow cytometer (Thermo Fisher Scientific, Waltham, MA, USA).

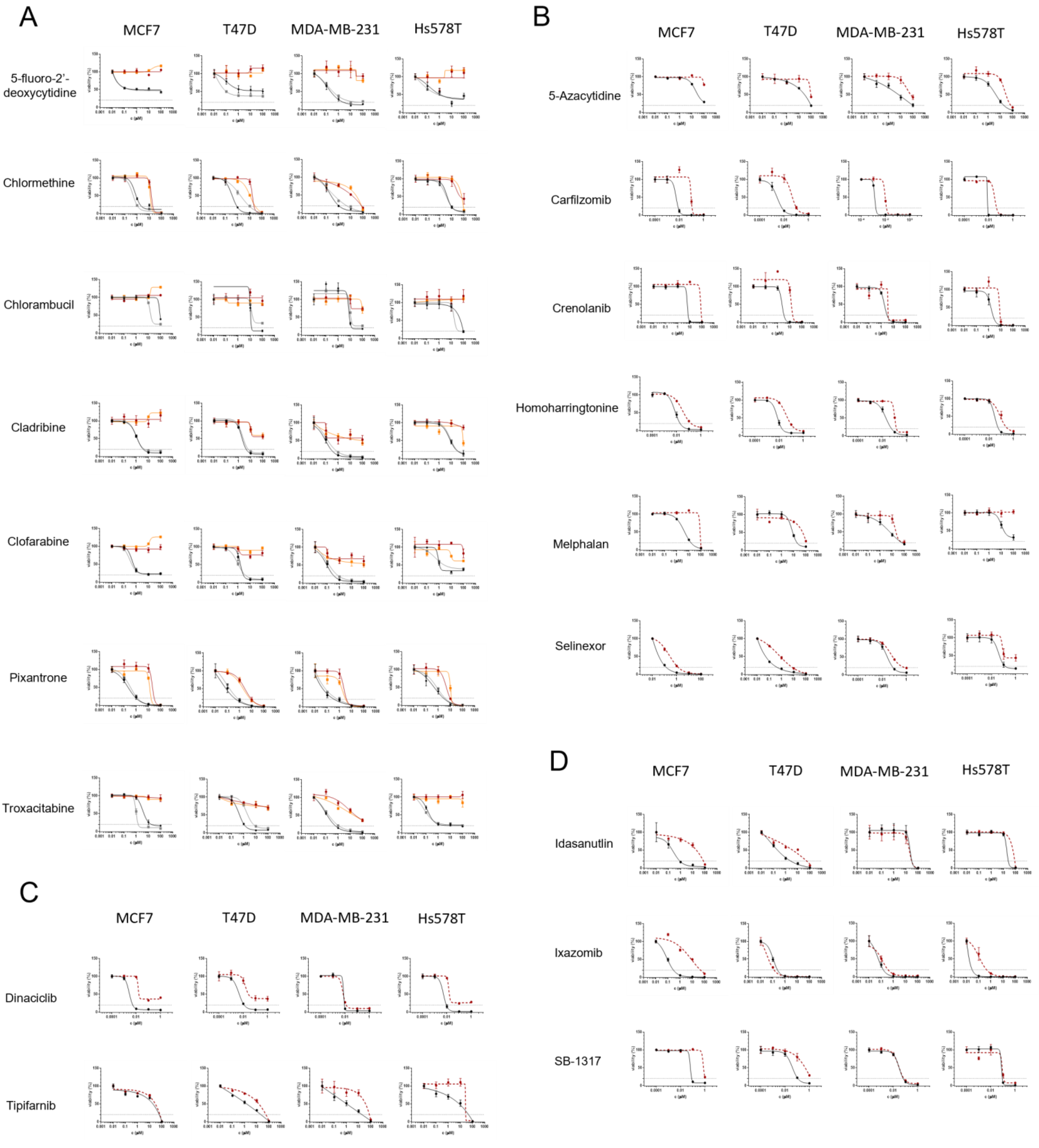
A
MCF7
T47D
MDA-MB-231
Hs578T
5-fluoro-2'-deoxycytidine
Chlormethine
Chlorambucil
Cladribine
Clofarabine
Pixantrone
Troxacitabine
B
MCF7
T47D
MDA-MB-231
Hs578T
5-Azacytidine
Carfilzomib
Crenolanib
Homoharringtonine
Melphalan
Selinexor
C
MCF7
T47D
MDA-MB-231
Hs578T
Dinaciclib
Tipifarnib
D
MCF7
T47D
MDA-MB-231
Hs578T
Idasanutlin
Ixazomib
SB-1317

Supplementary Figure S3. Cytotoxicity curves of FDA-approved drugs measured on CTR, TIS, REPOP, and re-TIS breast cancer cells.

(A) Dose-response curves comparing the sensitivity of CTR (black), TIS (red), REPOP (gray), and re-TIS (orange) cells treated with seven FDA-approved compounds. This panel expands upon the findings presented in Figure 2C.

(B) Cytotoxicity of six compounds in CTR (black) and TIS (red) cells. These drugs were largely ineffective in targeting TIS cells across tested conditions.

(C) Cytotoxicity of compounds that demonstrated limited efficacy in eliminating TIS cells in three out of four breast cancer cell lines. Responses in CTR (black) and TIS (red) cells are shown.

(D) Cytotoxicity of compounds with limited efficacy in two out of four breast cancer cell lines. Sensitivity curves for CTR (black) and TIS (red) cells are presented.

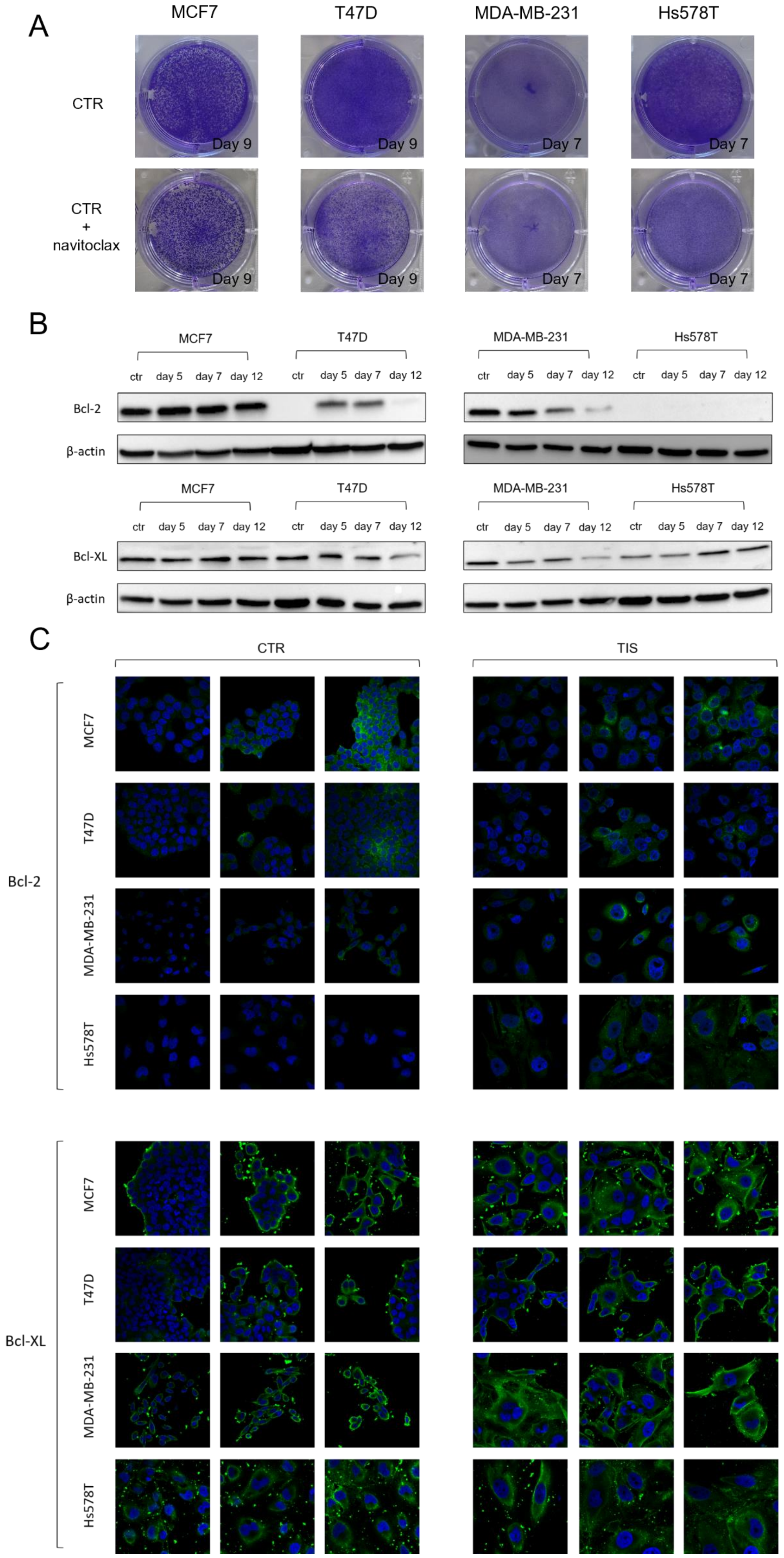
A
MCF7
T47D
MDA-MB-231
Hs578T
CTR
CTR + navitoclax
Day 9
Day 7
B
ctr
day 5
day 7
day 12
Bcl-2
β-actin
Bcl-XL
C
CTR
TIS

D

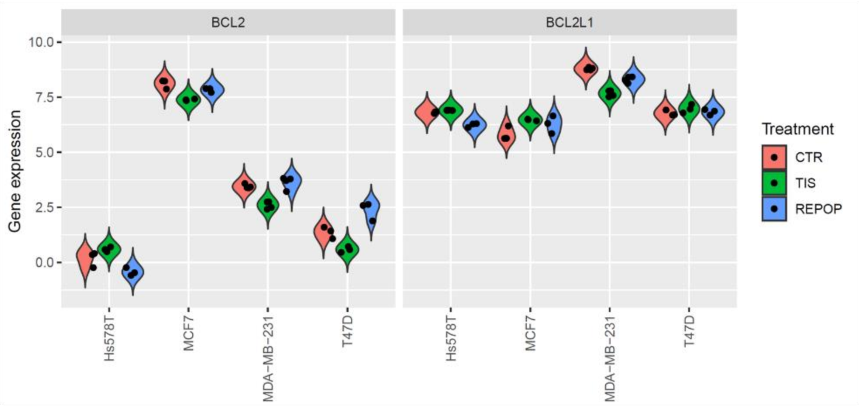
BCL2
BCL2L1
Gene expression
10.0
7.5
5.0
2.5
0.0
Hs578T
MCF7
MDA−MB−231
T47D
Treatment
CTR
TIS
REPOP

E

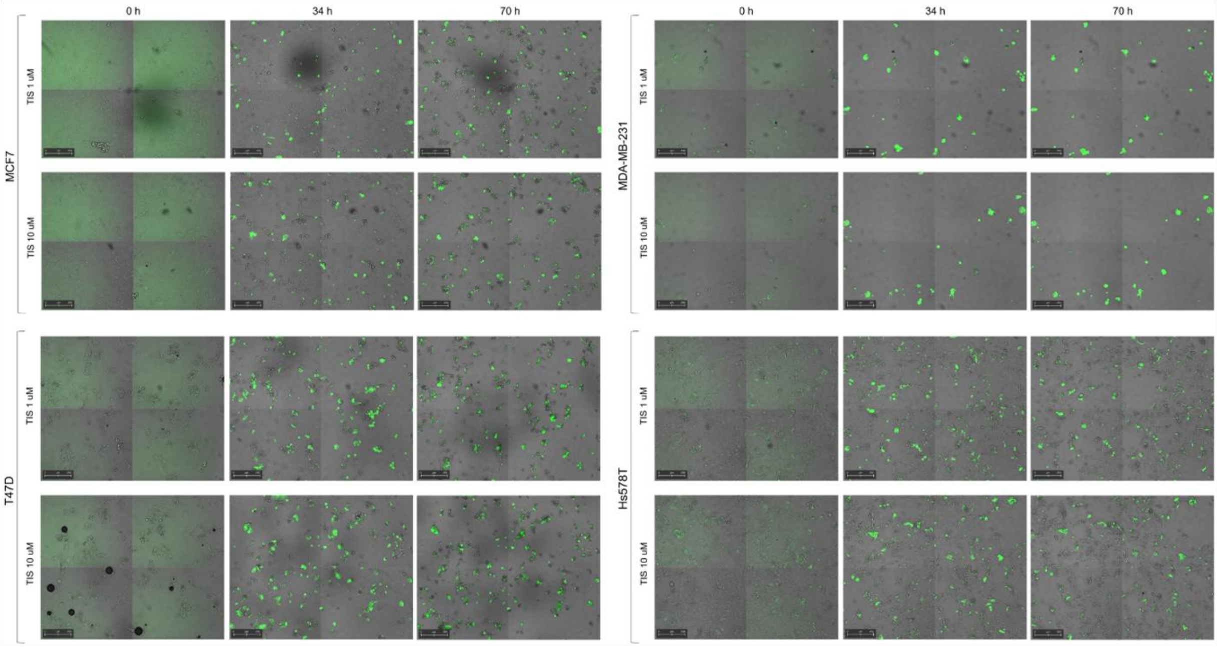
0 h
34 h
70 h
MCF7
TIS 1 uM
TIS 10 uM
MDA-MB-231
T47D
Hs578T

F

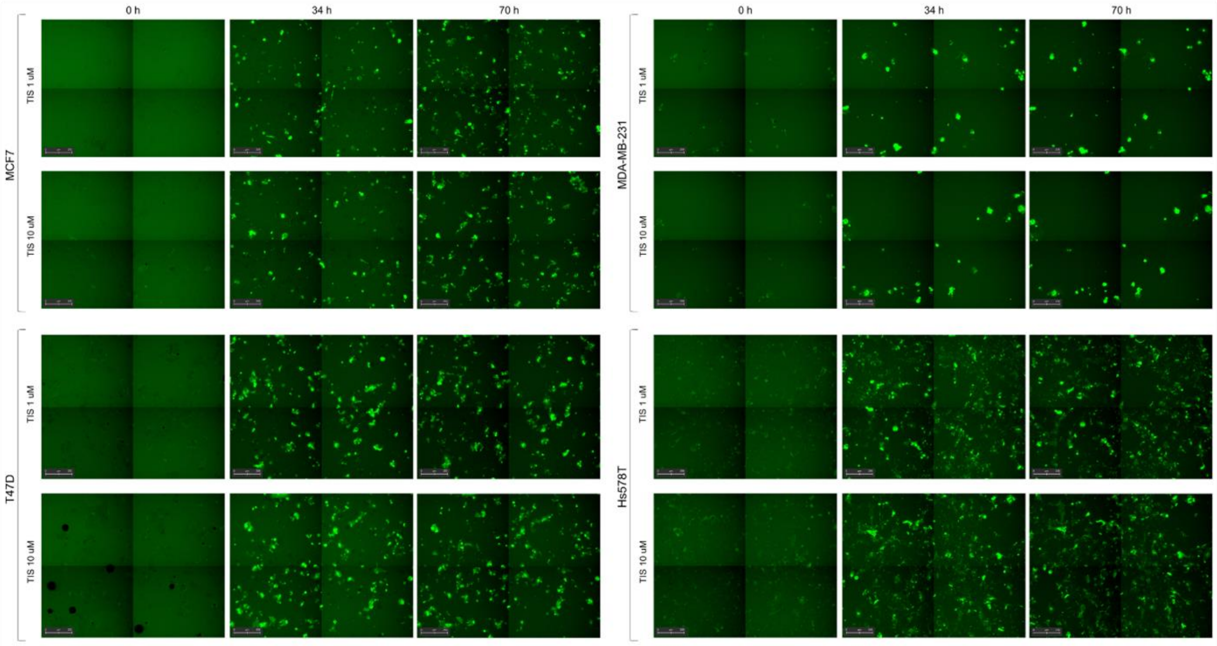
0 h
34 h
70 h
MCF7
TIS 1 uM
TIS 10 uM
MDA-MB-231
T47D
Hs578T

G

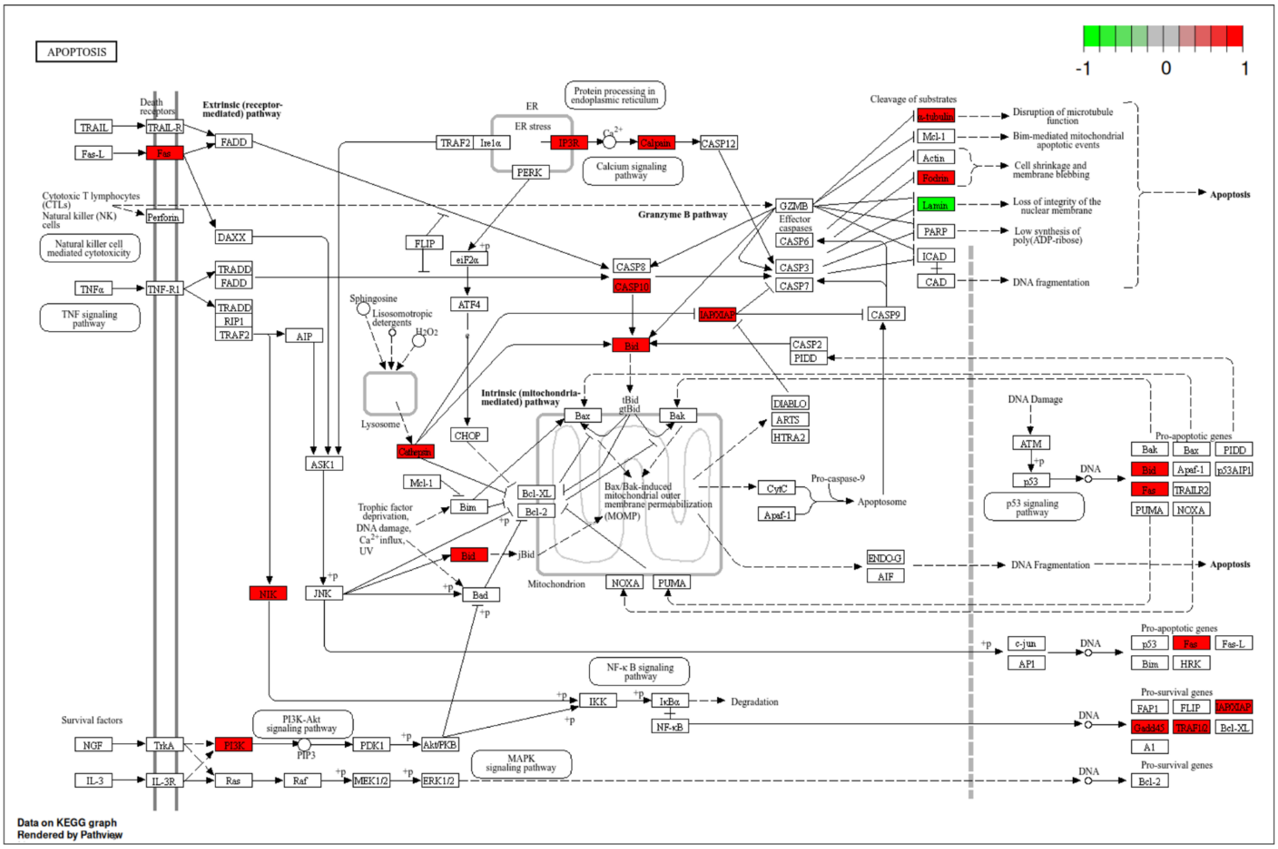
APOPTOSIS
-1
0
1
Death receptors
Extrinsic (receptor-mediated) pathway
TRAIL
TRAIL-R
Fas-L
Fas
FADD
Cytotoxic T lymphocytes (CTLs)
Natural killer (NK) cells
Perforin
Natural killer cell mediated cytotoxicity
DAXX
TNFα
TNF-R1
TRADD
RIP1
TRAF2
TNF signaling pathway
ER
ER stress
Ire1α
IP3R
Calpain
CASP12
PERK
Protein processing in endoplasmic reticulum
Calcium signaling pathway
FLIP
eiF2α
ATF4
CHOP
Sphingosine
Lysosomotropic detergents
H2O2
Lysosome
Cathepsin
AIP
ASK1
JNK
NIK
CASP8
CASP10
IAP/XIAP
Bid
CASP2
PIDD
Intrinsic (mitochondria-mediated) pathway
tBid
Bax
Bak
Mcl-1
Bim
Bcl-XL
Bcl-2
Bad
jBid
Trophic factor deprivation, DNA damage, Ca2+ influx, UV
Bax/Bak-induced mitochondrial outer membrane permeabilization (MOMP)
Mitochondrion
NOXA
PUMA
DIABLO
ARTS
HTRA2
CytC
Apaf-1
Pro-caspase-9
Apoptosome
CASP9
ENDO-G
AIF
DNA Fragmentation
GZMB
Effector caspases
CASP6
CASP3
CASP7
Granzyme B pathway
Cleavage of substrates
α-tubulin
Actin
Fodrin
Lamin
PARP
ICAD
CAD
Disruption of microtubule function
Bim-mediated mitochondrial apoptotic events
Cell shrinkage and membrane blebbing
Loss of integrity of the nuclear membrane
Low synthesis of poly(ADP-ribose)
DNA fragmentation
Apoptosis
DNA Damage
ATM
p53
p53 signaling pathway
DNA
Pro-apoptotic genes
PIDD
p53AIP1
TRAILR2
c-jun
AP1
HRK
NF-κB signaling pathway
IKK
IκBα
NF-κB
Degradation
Pro-survival genes
IAP1
IAP/XIAP
Gadd45
TRAF1/2
A1
Survival factors
NGF
TrkA
PI3K
PIP3
PDK1
Akt/PKB
PI3K-Akt signaling pathway
IL-3
IL-3R
Ras
Raf
MEK1/2
ERK1/2
MAPK signaling pathway
Data on KEGG graph
Rendered by Pathview

H

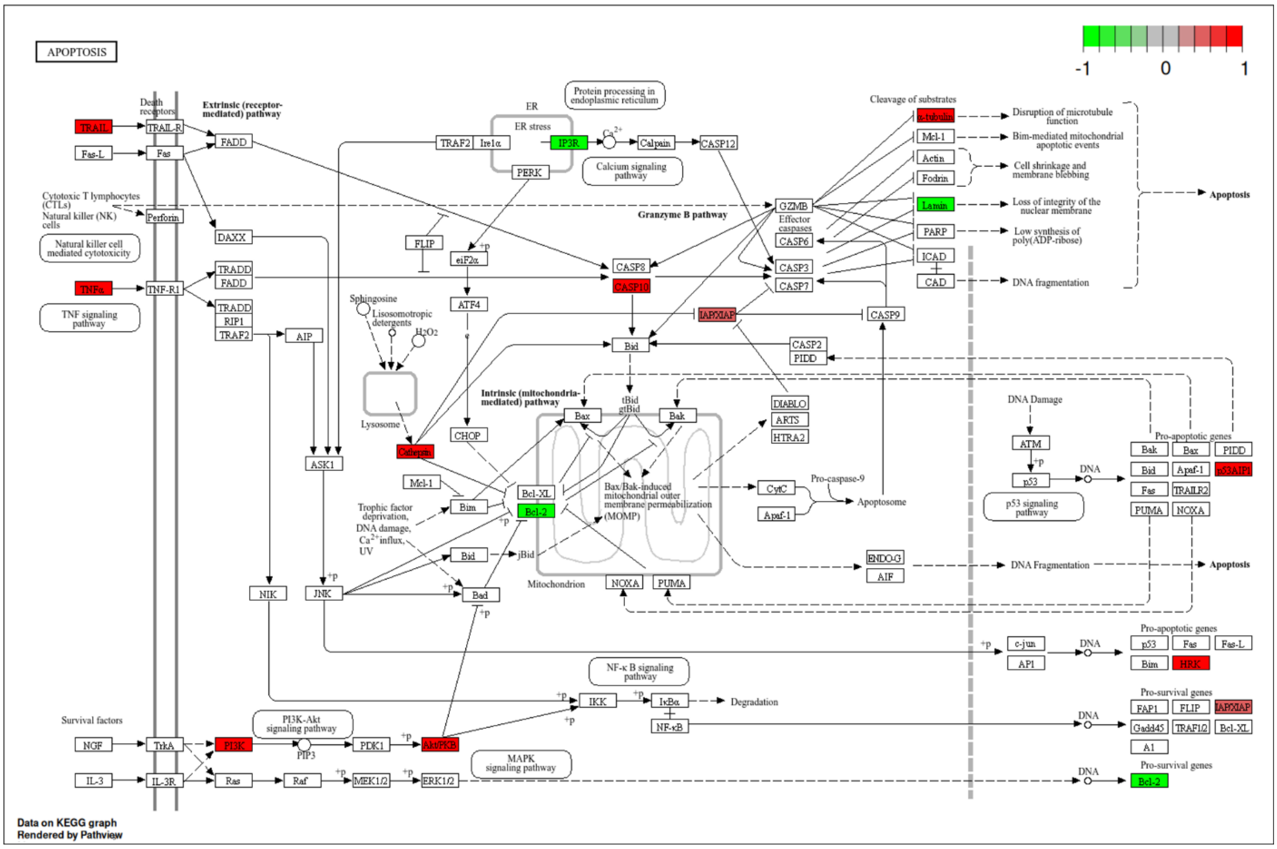
APOPTOSIS
-1
0
1
Death receptors
Extrinsic (receptor-mediated) pathway
TRAIL
TRAIL-R
Fas-L
Fas
FADD
Cytotoxic T lymphocytes (CTLs)
Natural killer (NK) cells
Perforin
Natural killer cell mediated cytotoxicity
DAXX
TNFα
TNF-R1
TRADD
RIP1
TRAF2
TNF signaling pathway
ER
ER stress
Ire1α
IP3R
Calpain
CASP12
PERK
Protein processing in endoplasmic reticulum
Calcium signaling pathway
FLIP
eiF2α
ATF4
CHOP
Sphingosine
Lysosomotropic detergents
H2O2
Lysosome
Cathepsin
AIP
ASK1
JNK
NIK
CASP8
CASP10
IAP/XIAP
Bid
CASP2
PIDD
Intrinsic (mitochondria-mediated) pathway
tBid
Bax
Bak
Mcl-1
Bim
Bcl-XL
Bcl-2
Bad
jBid
Trophic factor deprivation, DNA damage, Ca2+ influx, UV
Bax/Bak-induced mitochondrial outer membrane permeabilization (MOMP)
Mitochondrion
NOXA
PUMA
DIABLO
ARTS
HTRA2
CytC
Apaf-1
Pro-caspase-9
Apoptosome
CASP9
ENDO-G
AIF
DNA Fragmentation
GZMB
Effector caspases
CASP6
CASP3
CASP7
Granzyme B pathway
Cleavage of substrates
α-tubulin
Actin
Fodrin
Lamin
PARP
ICAD
CAD
Disruption of microtubule function
Bim-mediated mitochondrial apoptotic events
Cell shrinkage and membrane blebbing
Loss of integrity of the nuclear membrane
Low synthesis of poly(ADP-ribose)
DNA fragmentation
Apoptosis
DNA Damage
ATM
p53
p53 signaling pathway
DNA
Pro-apoptotic genes
PIDD
p53AIP1
TRAILR2
c-jun
AP1
HRK
NF-κB signaling pathway
IKK
IκBα
NF-κB
Degradation
Pro-survival genes
IAP1
IAP/XIAP
Gadd45
TRAF1/2
A1
Survival factors
NGF
TrkA
PI3K
PIP3
PDK1
Akt/PKB
PI3K-Akt signaling pathway
IL-3
IL-3R
Ras
Raf
MEK1/2
ERK1/2
MAPK signaling pathway
Data on KEGG graph
Rendered by Pathview

Supplementary Figure S4. Impact of Bcl-2 and Bcl-XL expression on senolytic efficacy against TIS cells.

(A) Navitoclax treatment does not affect viability or growth kinetics of CTR breast cancer cells significantly, indicating selective activity against TIS cells.

(B) Temporal dynamics of Bcl-2 and Bcl-XL expression over 12 days of TIS induction, highlighting key shifts during senescence establishment.

(C) Analysis of Bcl-2 and Bcl-XL expression patterns, heterogeneity, and subcellular localization in CTR and TIS breast cancer cells at day 12 of TIS induction, revealing potential associations with senolytic resistance mechanisms.

(D) mRNA expression levels of BCL2 and BCL2L1 genes in CTR, TIS and REPOP cells across four breast cancer cell lines.

(E) Composite images of brightfield and GFP (Annexin V-FITC) fluorescent channels captured during live-cell monitoring of apoptosis in TIS cells induced by 1 and 10 µM belinostat treatment. Rows show different drug concentrations, while different timepoints (0, 34 and 70 hours) can be seen in the columns.

(F) GFP channel isolated from panel E.

(G) Expression changes of genes involved in Apoptosis in MCF7 TIS cells. Green and red marking show increased and decreased expression in TIS cells, respectively, compared to CTR.

(H) Expression changes of genes involved in Apoptosis in T47D TIS cells. Green and red marking show increased and decreased expression in TIS cells, respectively, compared to CTR.

Apoptosis Live-cell Imaging

10.000 cells/well were plated in 96-well plates. TIS induction was performed as described above. After TIS induction cells were counted in three wells and the results were averaged, after which the appropriate number of CTR cells was plated. The following day cells were washed twice with PBS, then 200 µL of FluoroBrite (Thermo Fisher Scientific, Waltham, MA, USA) containing 1 µL of Annexin V-FITC and 1 µL of Annexin V binding buffer (AD10, DOJINDO, Japan) was added to each well, supplemented with either 1 µM or 10 µM Belinostat. Time-lapse videos were recorded with the JuLI™ Stage fluorescence live-cell imaging system (NanoEntek, Korea), JuLI Stat was used for the evaluation of the images.

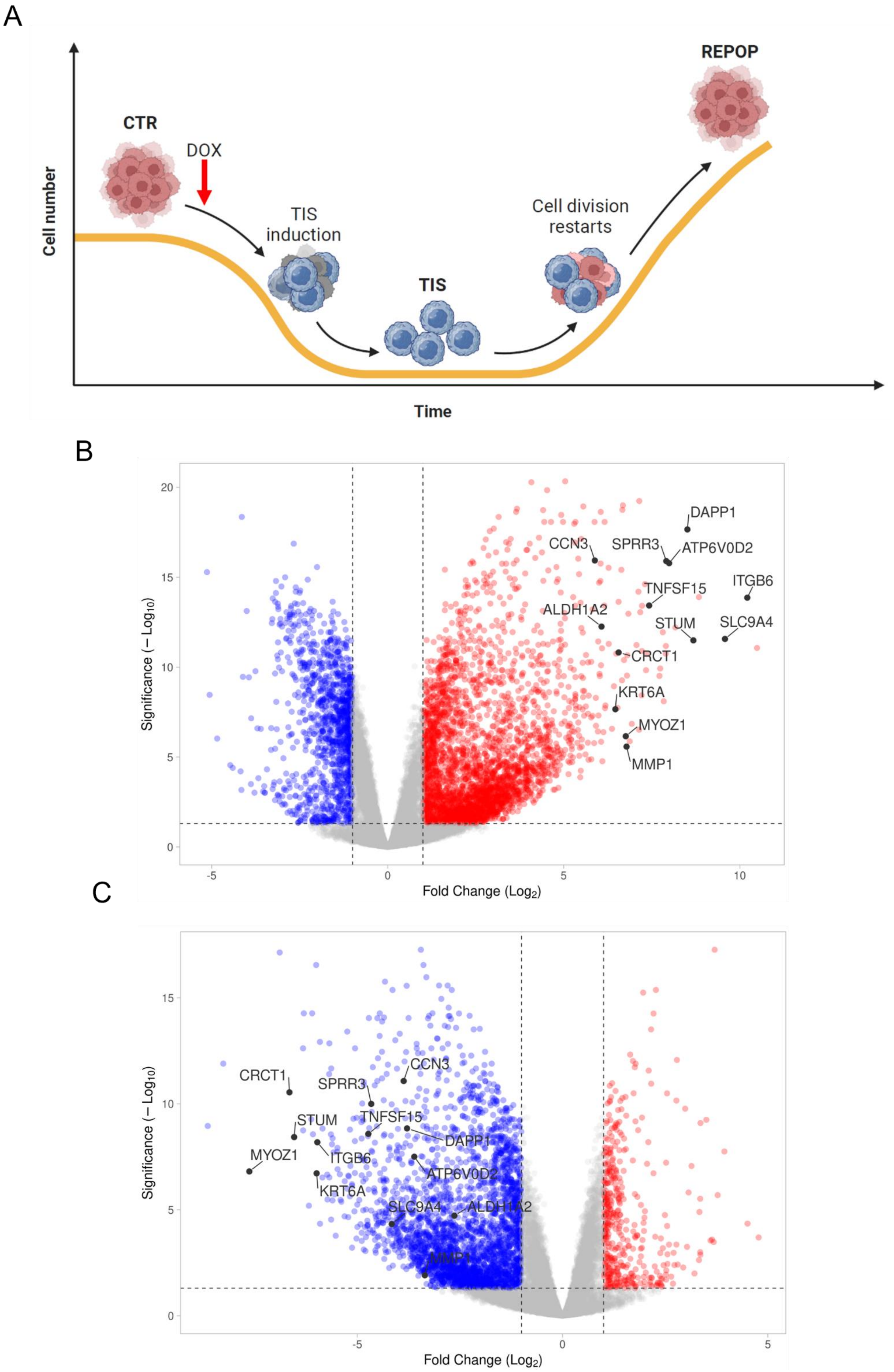
A
Cell number
Time
CTR
DOX
TIS
induction
TIS
Cell division
restarts
REPOP
B
Significance (− Log10)
Fold Change (Log2)
DAPP1
CCN3
SPRR3
ATP6V0D2
ITGB6
TNFSF15
ALDH1A2
STUM
SLC9A4
CRCT1
KRT6A
MYOZ1
MMP1
C
Significance (− Log10)
Fold Change (Log2)
CRCT1
SPRR3
CCN3
STUM
TNFSF15
DAPP1
MYOZ1
ITGB6
ATP6V0D2
KRT6A
SLC9A4
ALDH1A2
MMP1

Supplementary Figure S5. Schematic representation and transcriptional reprogramming during therapy-induced senescence and repopulation in breast cancer cells.

(A) Schematic representation of therapy-induced senescence (TIS) and subsequent escape leading to repopulation (REPOP) in breast cancer cells. Doxorubicin (DOX) treatment induces TIS, characterized by a transient growth arrest. Over time, a subset of cells overcomes senescence, re-entering the cell cycle and forming the repopulated (REPOP) population.

(B) Volcano plot displaying differentially expressed genes (DEGs) between TIS and control (CTR) cells. Significantly upregulated genes in TIS cells are shown in red, downregulated genes in blue, and non-significant genes in gray. The most overexpressed genes are labeled.

(C) Volcano plot comparing gene expression between REPOP and TIS cells. Upregulated genes in REPOP cells are highlighted in red, while downregulated genes are in blue. The shift in gene expression suggests that REPOP cells undergo a distinct transcriptional reprogramming compared to their senescent precursors.

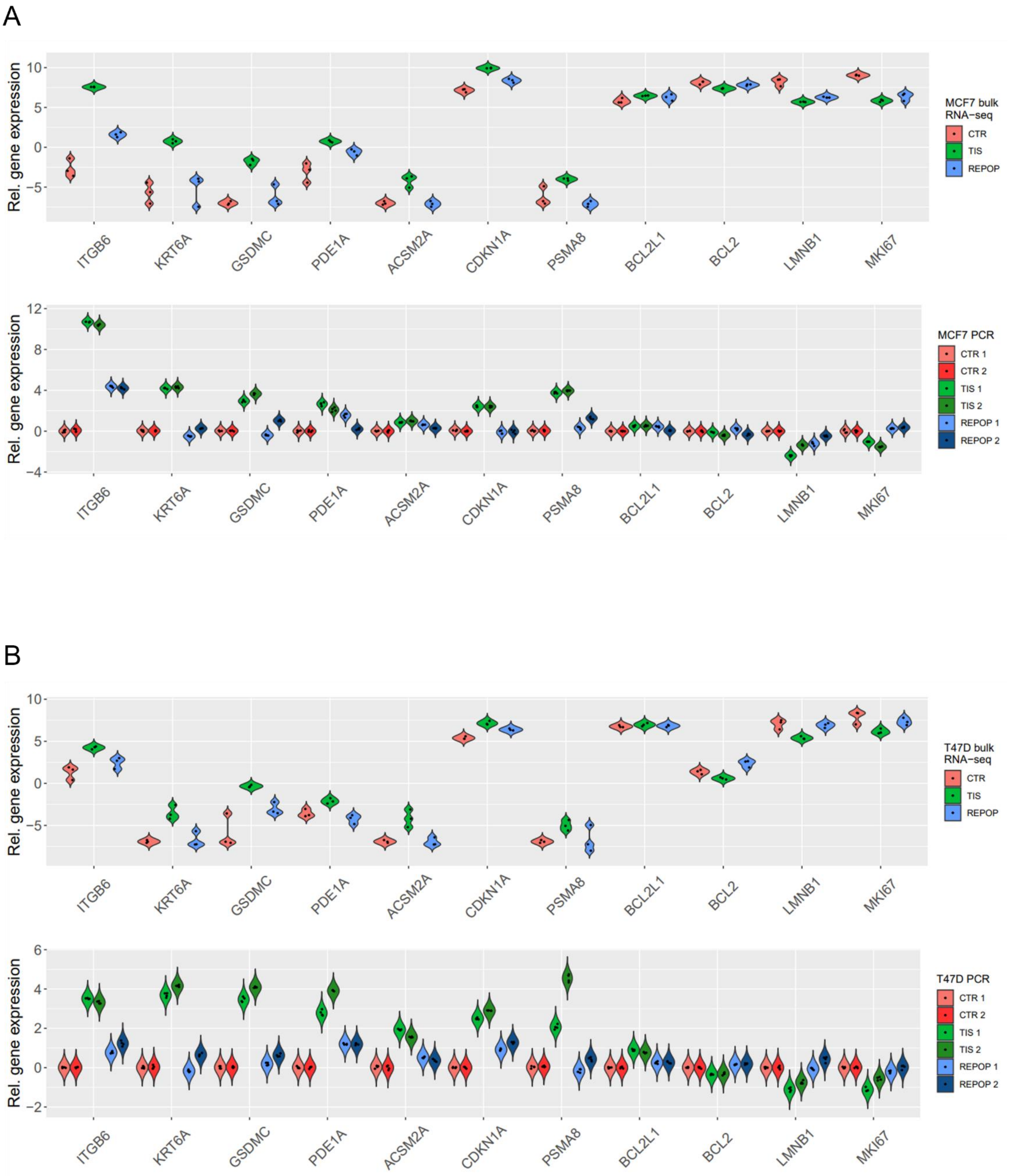
A
B
Rel. gene expression
ITGB6
KRT6A
GSDMC
PDE1A
ACSM2A
CDKN1A
PSMA8
BCL2L1
BCL2
LMNB1
MKI67
MCF7 bulk RNA−seq
CTR
TIS
REPOP
MCF7 PCR
CTR 1
CTR 2
TIS 1
TIS 2
REPOP 1
REPOP 2
T47D bulk RNA−seq
T47D PCR

C

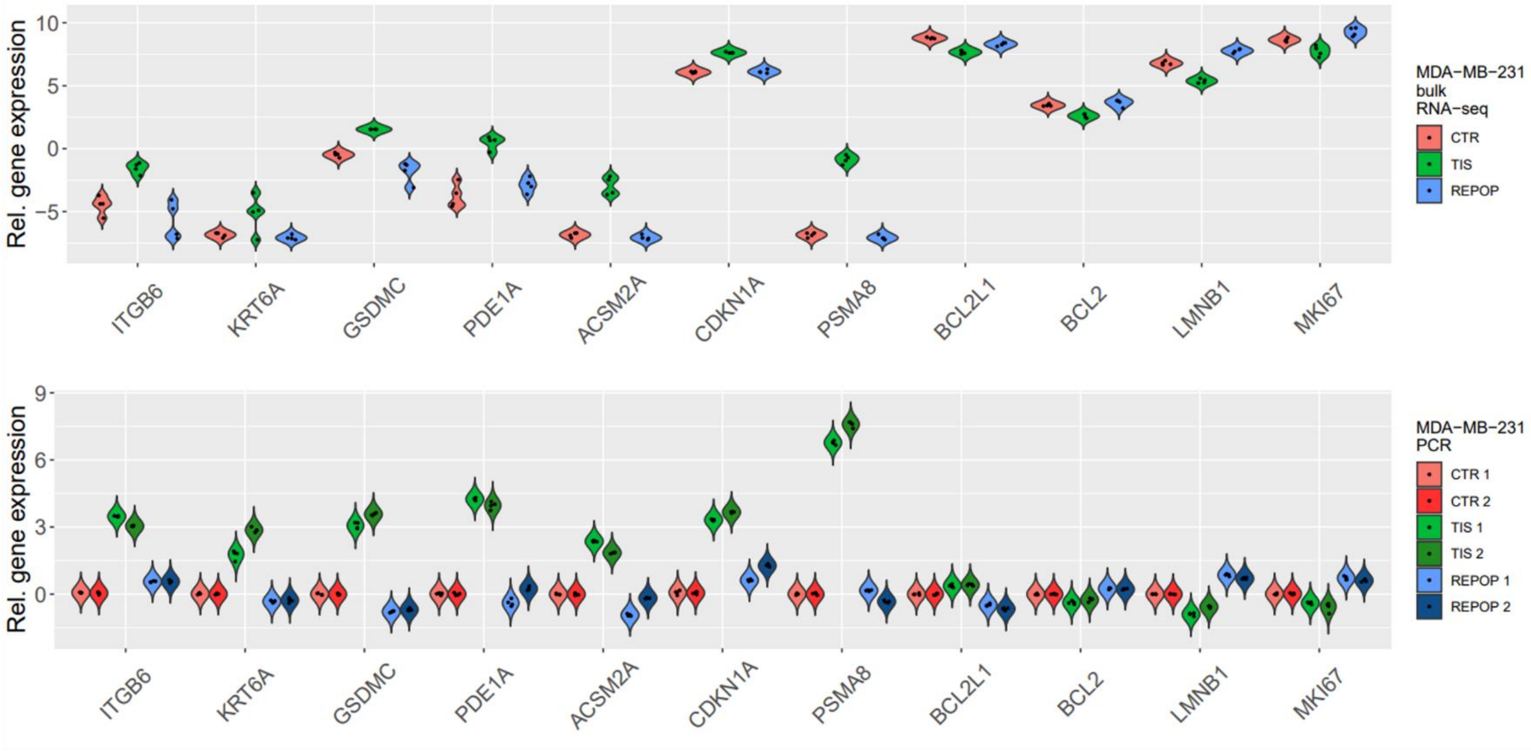
Rel. gene expression
10
5
0
-5
ITGB6
KRT6A
GSDMC
PDE1A
ACSM2A
CDKN1A
PSMA8
BCL2L1
BCL2
LMNB1
MKI67
MDA−MB−231
bulk
RNA−seq
CTR
TIS
REPOP
Rel. gene expression
9
6
3
0
MDA−MB−231
PCR
CTR 1
CTR 2
TIS 1
TIS 2
REPOP 1
REPOP 2

D

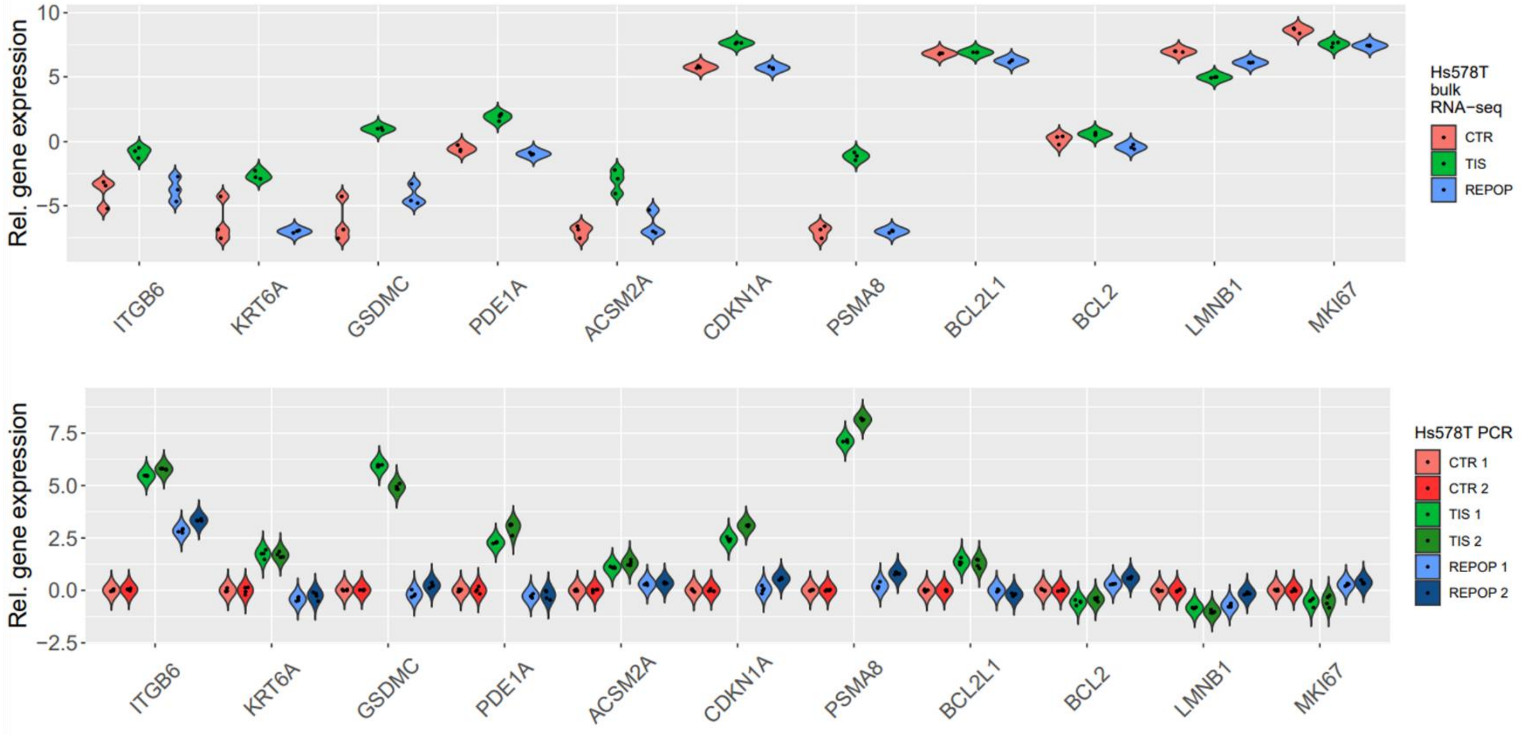
Rel. gene expression
10
5
0
-5
ITGB6
KRT6A
GSDMC
PDE1A
ACSM2A
CDKN1A
PSMA8
BCL2L1
BCL2
LMNB1
MKI67
Hs578T
bulk
RNA−seq
CTR
TIS
REPOP
Rel. gene expression
7.5
5.0
2.5
0.0
−2.5
Hs578T PCR
CTR 1
CTR 2
TIS 1
TIS 2
REPOP 1
REPOP 2

Supplementary Figure S6. Bulk RNA-seq and qPCR validation of gene expression in all breast cancer cell lines

(A) MCF7 cell line: The top panel presents relative gene expression levels measured by bulk RNA sequencing, while the bottom panel shows qPCR validation results of two biological replicates of each group (CTR, TIS, REPOP) measured by four technical replicates.

(B) T47D cell line: The top panel presents relative gene expression levels measured by bulk RNA sequencing, while the bottom panel shows qPCR validation results of two biological replicates of each group (CTR, TIS, REPOP) measured by four technical replicates.

(C) MDA-MB-231 cell line: The top panel presents relative gene expression levels measured by bulk RNA sequencing, while the bottom panel shows qPCR validation results of two biological replicates of each group (CTR, TIS, REPOP) measured by four technical replicates.

(D) Hs578T cell line: The top panel presents relative gene expression levels measured by bulk RNA sequencing, while the bottom panel shows qPCR validation results of two biological replicates of each group (CTR, TIS, REPOP) measured by four technical replicates.

Color coding represents different experimental conditions, including control (CTR), treatment-induced senescence (TIS), and reprogrammed (REPOP) cells.

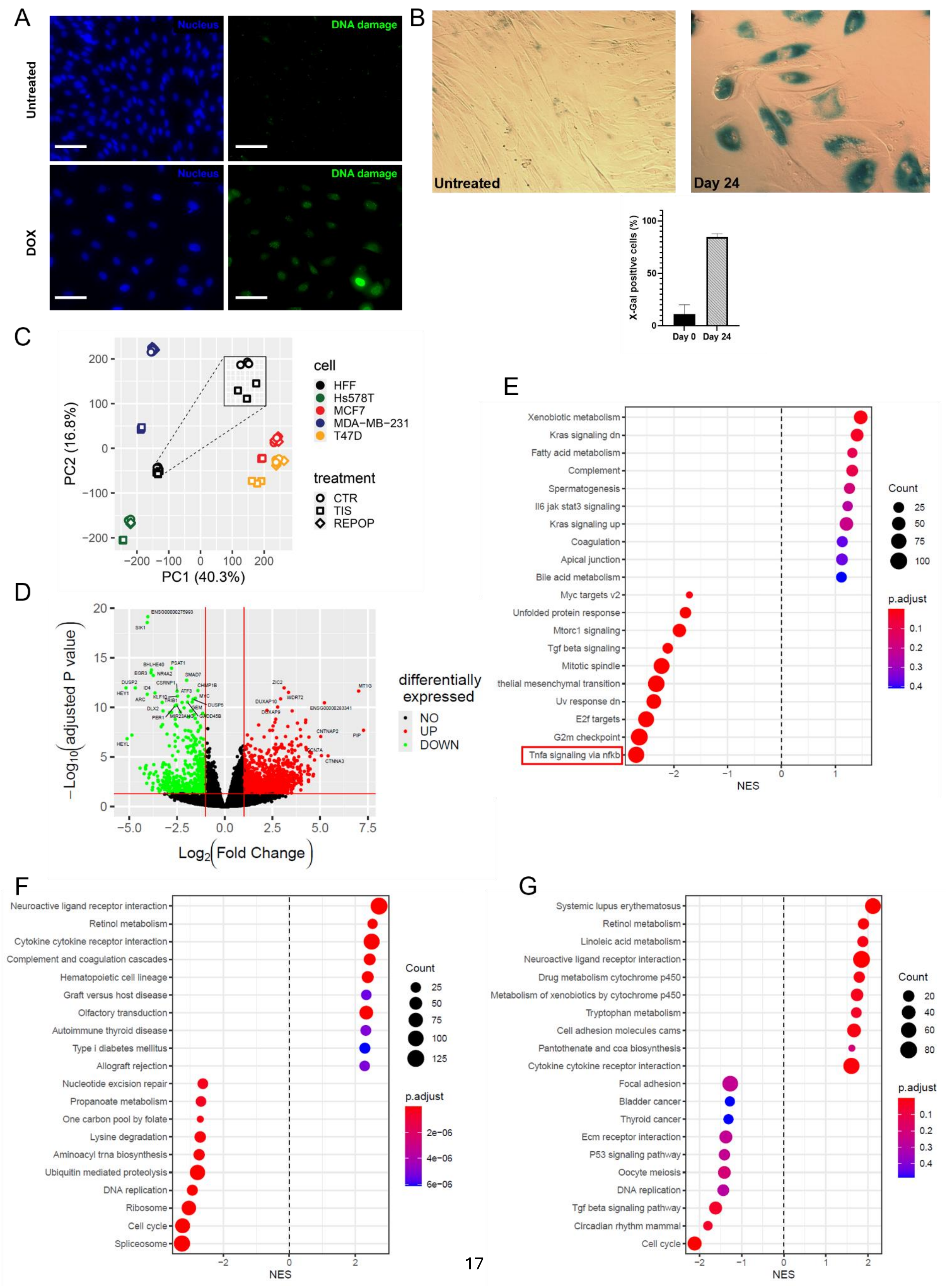

A
Untreated
DOX
Nucleus
DNA damage
B
Untreated
Day 24
X-Gal positive cells (%)
100
50
0
Day 0
Day 24
C
PC2 (16.8%)
PC1 (40.3%)
cell
HFF
Hs578T
MCF7
MDA−MB−231
T47D
treatment
CTR
TIS
REPOP
D
−Log10(adjusted P value)
Log2(Fold Change)
differentially expressed
NO
UP
DOWN
E
Xenobiotic metabolism
Kras signaling dn
Fatty acid metabolism
Complement
Spermatogenesis
Il6 jak stat3 signaling
Kras signaling up
Coagulation
Apical junction
Bile acid metabolism
Myc targets v2
Unfolded protein response
Mtorc1 signaling
Tgf beta signaling
Mitotic spindle
thelial mesenchymal transition
Uv response dn
E2f targets
G2m checkpoint
Tnfa signaling via nfkb
NES
Count
p.adjust
F
Neuroactive ligand receptor interaction
Retinol metabolism
Cytokine cytokine receptor interaction
Complement and coagulation cascades
Hematopoietic cell lineage
Graft versus host disease
Olfactory transduction
Autoimmune thyroid disease
Type i diabetes mellitus
Allograft rejection
Nucleotide excision repair
Propanoate metabolism
One carbon pool by folate
Lysine degradation
Aminoacyl trna biosynthesis
Ubiquitin mediated proteolysis
DNA replication
Ribosome
Cell cycle
Spliceosome
NES
Count
p.adjust
G
Systemic lupus erythematosus
Retinol metabolism
Linoleic acid metabolism
Neuroactive ligand receptor interaction
Drug metabolism cytochrome p450
Metabolism of xenobiotics by cytochrome p450
Tryptophan metabolism
Cell adhesion molecules cams
Pantothenate and coa biosynthesis
Cytokine cytokine receptor interaction
Focal adhesion
Bladder cancer
Thyroid cancer
Ecm receptor interaction
P53 signaling pathway
Oocyte meiosis
DNA replication
Tgf beta signaling pathway
Circadian rhythm mammal
Cell cycle
NES
Count
p.adjust

Supplementary Figure S7. Characterization of therapy-induced senescence (TIS) in non-malignant HFF cells.

(A) Visualization of DNA double-strand breaks (green) in HFF cells during TIS induction by DOX treatment. Nuclei are counterstained with DAPI (blue).

(B) Senescence-associated β-galactosidase activity detected via X-gal staining at day 0 and day 24 of TIS induction. Bar graphs quantify β-gal staining intensity, confirming senescence induction over time.

(C) Principal component analysis (PCA) of CTR and TIS HFF cells, shown alongside malignant breast cancer cell lines from Figure 4C for comparative context.

(D) Volcano plot illustrating mRNA expression changes during the CTR-to-TIS transition in HFF cells, highlighting significant genes.

(E) Hallmark pathway analysis (MSigDB) based on RNA-seq data from CTR and TIS HFF cells. Upregulated and downregulated pathways are identified with FDR <0.05. The red box highlights pathways that are shared between healthy and malignant TIS cells but display opposite regulatory trends.

(F, G) Gene Ontology (GO) Biological Processes analysis of RNA-seq data. *(F)* Pathways identified in CTR vs. TIS transitions in four breast cancer cell lines. *(G)* Pathways identified in HFF cells. Upregulated and downregulated pathways are shown, with results filtered for FDR <0.05.

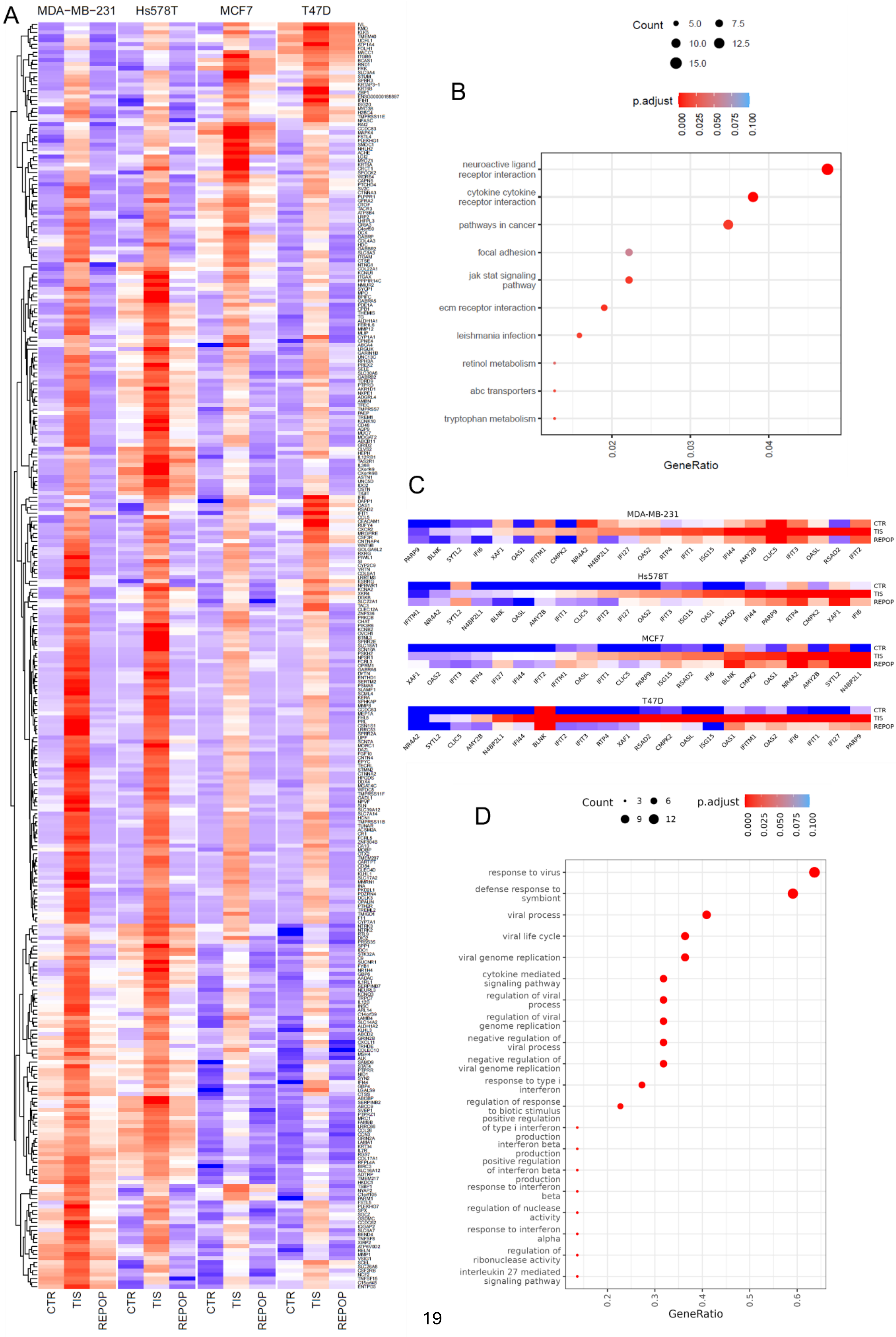
A
MDA−MB−231
Hs578T
MCF7
T47D
CTR
TIS
REPOP
B
Count
5.0
7.5
10.0
12.5
15.0
p.adjust
0.000
0.025
0.050
0.075
0.100
neuroactive ligand receptor interaction
cytokine cytokine receptor interaction
pathways in cancer
focal adhesion
jak stat signaling pathway
ecm receptor interaction
leishmania infection
retinol metabolism
abc transporters
tryptophan metabolism
0.02
0.03
0.04
GeneRatio
C
MDA-MB-231
Hs578T
MCF7
T47D
CTR
TIS
REPOP
D
Count
3
6
9
12
p.adjust
0.000
0.025
0.050
0.075
0.100
response to virus
defense response to symbiont
viral process
viral life cycle
viral genome replication
cytokine mediated signaling pathway
regulation of viral process
regulation of viral genome replication
negative regulation of viral process
negative regulation of viral genome replication
response to type i interferon
regulation of response to biotic stimulus
positive regulation of type i interferon production
interferon beta production
positive regulation of interferon beta production
response to interferon beta
regulation of nuclease activity
response to interferon alpha
regulation of ribonuclease activity
interleukin 27 mediated signaling pathway
0.2
0.3
0.4
0.5
0.6
GeneRatio

Supplementary Figure S8. Unique gene expression profiles of TIS shared across four breast cancer cell lines.

(A) Heatmap displaying 316 genes uniquely overexpressed in TIS cells, which return to baseline expression levels in REPOP cells, highlighting genes specific to the senescent state.

(B) KEGG pathway analysis of the 316 TIS-specific genes, identifying key pathways enriched in the senescent transcriptome.

(C) Heatmap showing the 22 genes consistently overexpressed in both TIS and REPOP cells but not in CTR cells, suggesting potential roles in persistent phenotypic alterations following senescence.

(D) Gene Ontology (GO) pathway analysis of the 22 overexpressed genes shared by TIS and REPOP cells, identifying biological processes relevant to sustained gene expression changes beyond the senescent state.

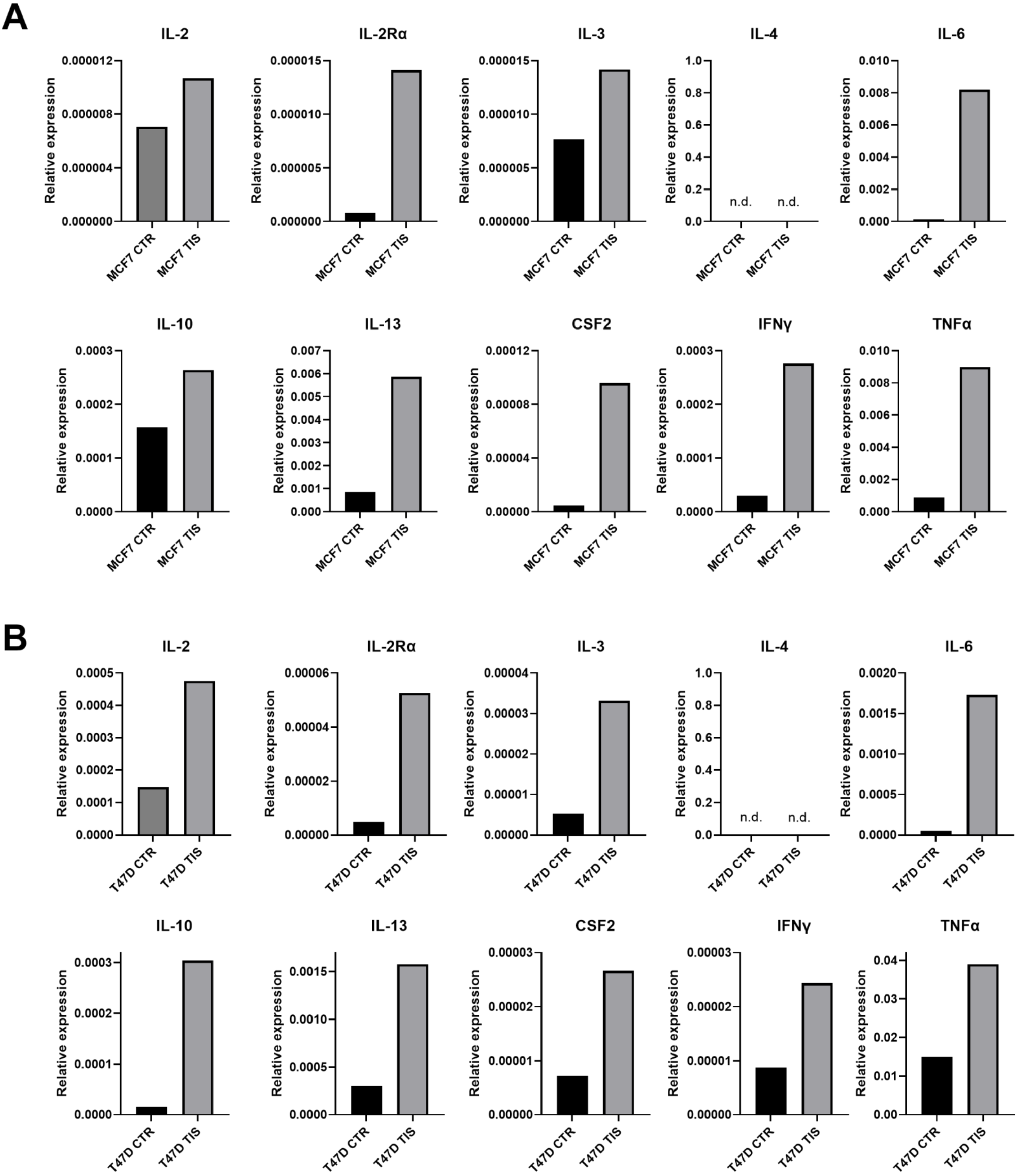
A
IL-2
IL-2Rα
IL-3
IL-4
IL-6
IL-10
IL-13
CSF2
IFNγ
TNFα
Relative expression
n.d.
MCF7 CTR
MCF7 TIS
B
T47D CTR
T47D TIS

Supplementary Figure S9. Cytokine expression profiling of CTR and TIS cells.

Cytokine expression profiling of CTR and TIS cells from *(A)* MCF7 or *(B)* T47D cultures. Relative expression of 10 immunosuppressing cytokines. Note that IL-4 was not detectable (n.d.) in neither cell line.

Cytokine Expression Assay

Total RNA was isolated from MCF7 and T47D parental (CTR) and senescent (TIS) cells using TRI Reagent® (Molecular Research Center, Cincinnati, OH), following the manufacturer's instructions. RNA was resuspended in 14 µL diethyl pyrocarbonate-treated water, and its concentration and purity were assessed using a NanoDrop 1000 spectrophotometer (Thermo Fisher Scientific, Waltham, MA). mRNA was reverse transcribed using the Maxima First Strand cDNA Synthesis Kit (Thermo Fisher Scientific) according to the manufacturer's protocol. Cytokine mRNA expression was analyzed using the high-throughput BioMark HD real-time qPCR platform (Fluidigm, South San Francisco, CA) with the Flex Six™ Gene Expression IFC chip (Fluidigm). Before qPCR analysis, a 12-cycle cDNA preamplification step and an exonuclease treatment were performed, followed by a 10-fold dilution of the final product. Cytokine expression levels were quantified using SsoFast™ EvaGreen® Supermix with Low ROX (Bio-Rad Laboratories, Hercules, CA) and primers specific to cytokines and reference genes, according to the manufacturer's recommendations. The cytokine panel included IL-2, IL-2Rα, IL-3, IL-4, IL-6, IL-10, IL-13, GM-CSF, TNFα, and IFNγ, while expression was normalized using four reference genes: G6PD, GAPDH, PPIB, and RPIIA. This cytokine expression assay was originally developed in our laboratory to assess immunosuppression efficiency in heart transplant patients receiving tacrolimus and methylprednisolone. Cytokine expression data were normalized to the average cycle threshold (Ct) of the four reference genes to ensure accurate quantification.

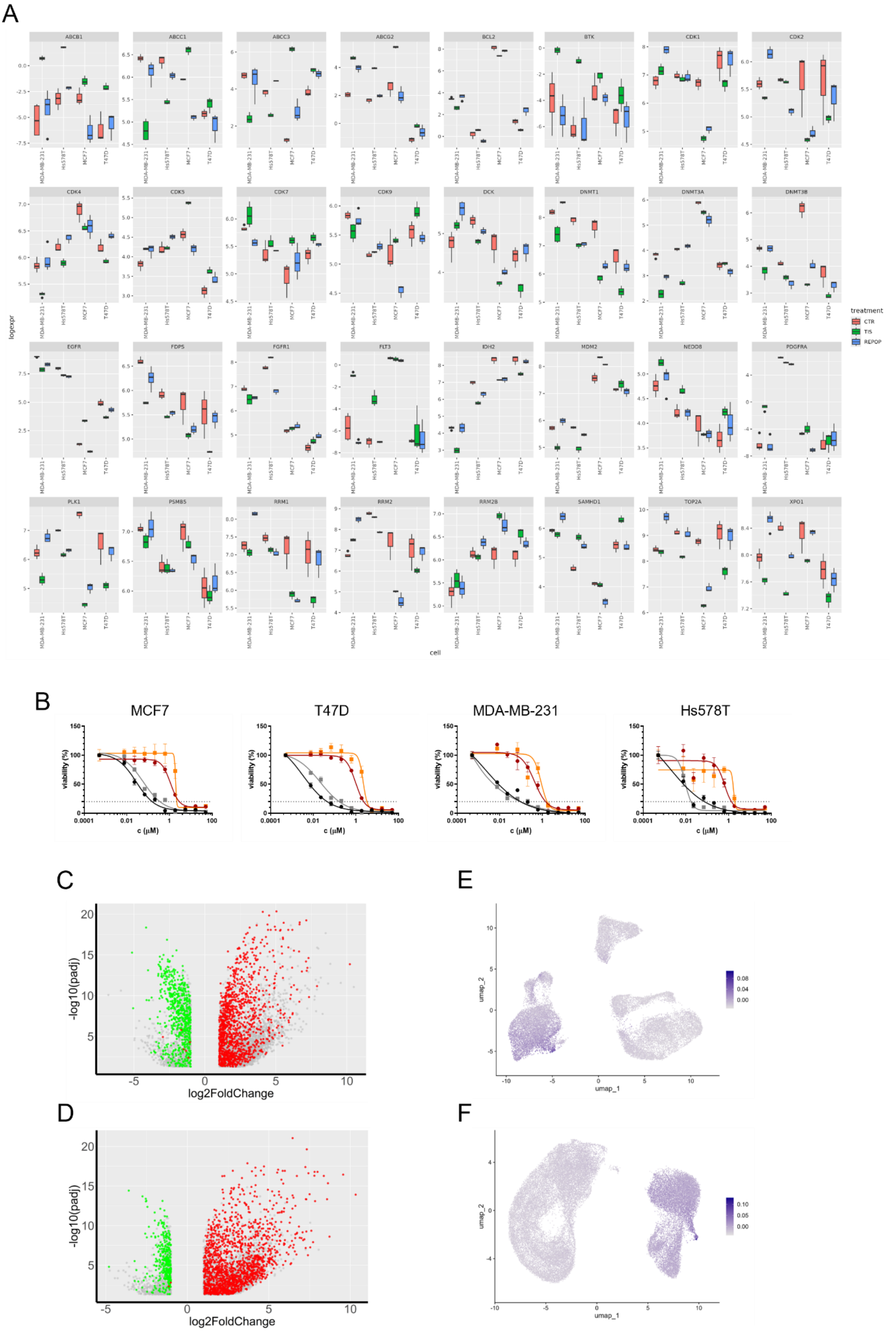
A
ABCB1
ABCC1
ABCC3
ABCG2
BCL2
BTK
CDK1
CDK2
CDK4
CDK5
CDK7
CDK9
DCK
DNMT1
DNMT3A
DNMT3B
EGFR
FDPS
FGFR1
FLT3
IDH2
MDM2
NEDD8
PDGFRA
PLK1
PSMB5
RRM1
RRM2
RRM2B
SAMHD1
TOP2A
XPO1
logexpr
cell
MDA-MB-231
Hs578T
MCF7
T47D
treatment
CTR
TIS
REPOP
B
MCF7
T47D
MDA-MB-231
Hs578T
viability (%)
c (µM)
C
-log10(padj)
log2FoldChange
D
-log10(padj)
log2FoldChange
E
umap_2
umap_1
F
umap_2
umap_1

Supplementary Figure S10. Investigation of drug resistance mechanisms and integration of bulk and single-cell RNA-seq data.

(A) Heatmap showing the expression levels of 32 genes associated with drug resistance or sensitivity in CTR, TIS, and REPOP cells. These genes provide insights into mechanisms underlying senescence-associated drug resistance.

(B) Effect of ABCB1 inhibition on drug sensitivity in CTR and TIS cells. DOX (50 μM/3) sensitivity was assessed in CTR (black) and TIS (red) cells without inhibitor and in CTR (gray) and TIS (orange) cells treated with 1 μM tariquidar, a potent third-generation ABCB1 inhibitor, highlighting ABCB1's role in drug resistance.

(C, D) Pseudo-bulk RNA-seq analysis derived from scRNA-seq data compared to bulk RNA-seq results for *(C)* MCF7 and *(D)* T47D breast cancer cell lines. This comparison demonstrates concordance between single-cell and bulk transcriptomic analyses.

(E, F) Projection of the 316 TIS-specific gene expression signature onto scRNA-seq data of *(E)* MCF7 and *(F)* T47D cells, showing strong correlation with the TIS population, confirming the robustness of the TIS-specific transcriptomic profile in single-cell analyses.

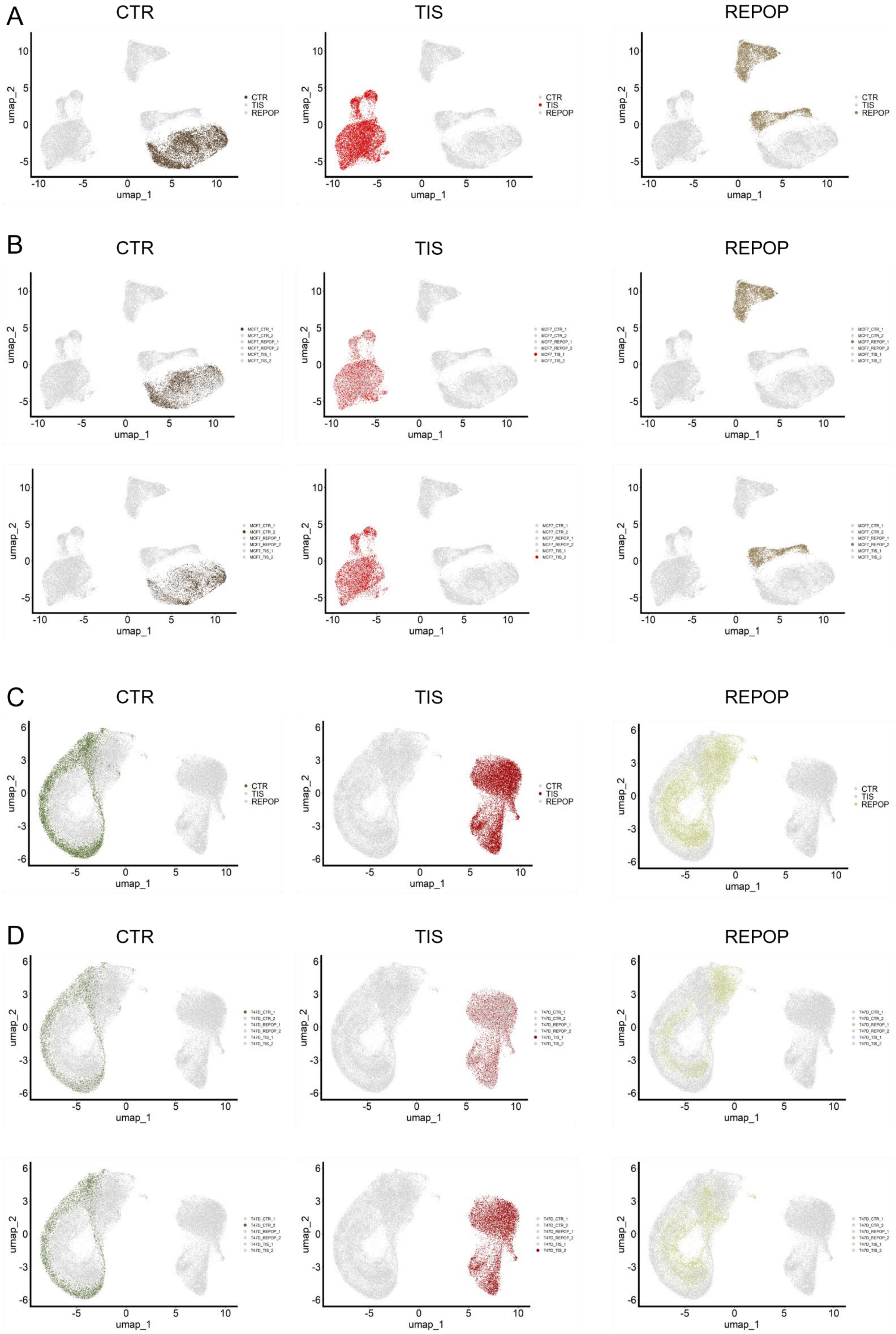
A
B
C
D
CTR
TIS
REPOP
umap_1
umap_2
CTR
TIS
REPOP
MCF7_CTR_1
MCF7_CTR_2
MCF7_REPOP_1
MCF7_REPOP_2
MCF7_TIS_1
MCF7_TIS_2
T47D_CTR_1
T47D_CTR_2
T47D_REPOP_1
T47D_REPOP_2
T47D_TIS_1
T47D_TIS_2

Supplementary Figure S11. Batch comparison of scRNA-seq data across biological replicates for MCF7 and T47D cells.

(A, C) UMAP projections of combined biological replicates for MCF7 *(A)* and T47D *(C)* cells, illustrating the distribution of CTR (dark brown for MCF7, dark green for T47D), REPOP (light brown for MCF7, light green for T47D), and TIS (scarlet for MCF7, crimson for T47D) populations. These panels highlight the consistency of transcriptional landscapes across replicates.

(B, D) UMAP projections of individual biological replicates for MCF7 *(B)* and T47D *(D)* cells. Each replicate is displayed separately to highlight batch-specific variations and the overall reproducibility within the CTR (dark brown for MCF7, dark green for T47D), REPOP (light brown for MCF7, light green for T47D), and TIS (scarlet for MCF7, crimson for T47D) clusters. The top panels show replicate 1, while the bottom panels display replicate 2, clearly illustrating the similarities and differences between the biological replicates.

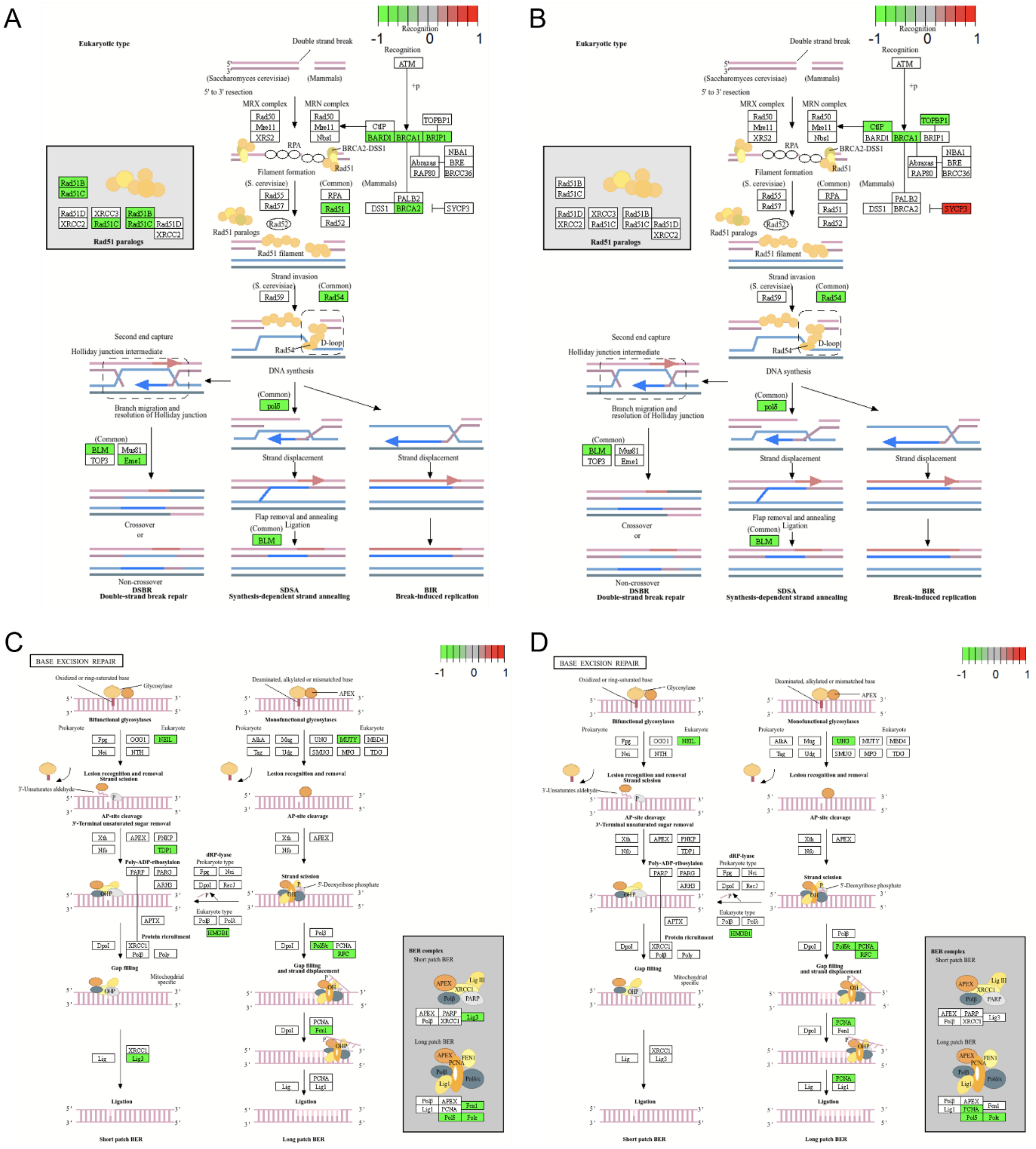

A
B
C
D
Eukaryotic type
Double strand break
Recognition
ATM
(Saccharomyces cerevisiae)
(Mammals)
5' to 3' resection
MRX complex
MRN complex
Rad50
Mre11
XRS2
Nbs1
CtIP
TOPBP1
BARD1
BRCA1
BRIP1
RPA
BRCA2-DSS1
NBA1
Abraxas
BRE
RAP80
BRCC36
Filament formation
(S. cerevisiae)
(Common)
(Mammals)
Rad55
Rad57
Rad51
Rad52
PALB2
DSS1
BRCA2
SYCP3
Rad51B
Rad51C
Rad51D
XRCC3
XRCC2
Rad51 paralogs
Rad51 filament
Strand invasion
Rad59
Rad54
Second end capture
Holliday junction intermediate
D-loop
DNA synthesis
polδ
Branch migration and resolution of Holliday junction
BLM
Mus81
TOP3
Eme1
Strand displacement
Flap removal and annealing
Ligation
Crossover
or
Non-crossover
DSBR
Double-strand break repair
SDSA
Synthesis-dependent strand annealing
BIR
Break-induced replication
-1
0
1
BASE EXCISION REPAIR
Oxidized or ring-saturated base
Glycosylase
Bifunctional glycosylases
Prokaryote
Eukaryote
Fpg
OGG1
NEIL
Nei
NTH
Deaminated, alkylated or mismatched base
APEX
Monofunctional glycosylases
AlkA
Mug
UNG
MUTY
MBD4
Tag
Udg
SMUG
MPG
TDG
Lesion recognition and removal
Strand scission
3'-Unsaturated aldehyde
AP-site cleavage
3'-Terminal unsaturated sugar removal
Xth
Nfo
PNKP
TDP1
Poly-ADP-ribosylation
PARP
PARG
ARH3
APTX
dRP-lyase
Prokaryote type
Eukaryote type
DpoI
RecJ
Polβ
Polλ
HMGB1
Protein ricruitment
XRCC1
Polι
Gap filling
Mitochondrial specific
5'-Deoxyribose phosphate
Polδ/ε
PCNA
RFC
Gap filling and strand displacement
Fen1
Lig
Lig3
Lig1
Short patch BER
Long patch BER
BER complex
Short patch BER
Lig III
Long patch BER
FEN1

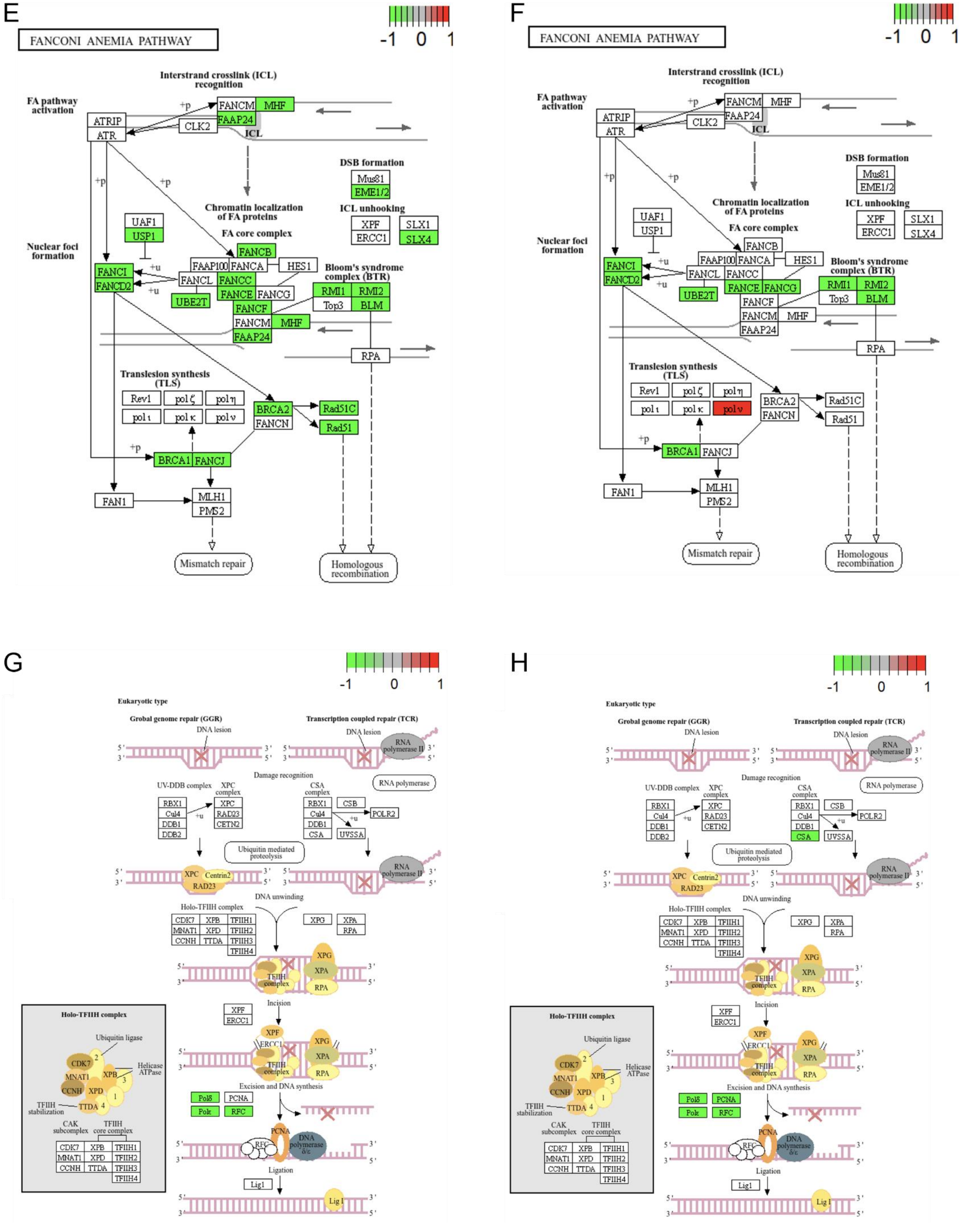
E
F
G
H
FANCONI ANEMIA PATHWAY
-1 0 1
Interstrand crosslink (ICL) recognition
FA pathway activation
ATRIP
ATR
CLK2
FANCM
MHF
FAAP24
ICL
DSB formation
Mus81
EME1/2
ICL unhooking
XPF
ERCC1
SLX1
SLX4
Chromatin localization of FA proteins
FA core complex
UAF1
USP1
Nuclear foci formation
FANCB
FAAP100
FANCA
HES1
FANCL
FANCC
FANCE
FANCG
FANCF
FANCI
FANCD2
UBE2T
Bloom's syndrome complex (BTR)
RMI1
RMI2
Top3
BLM
RPA
Translesion synthesis (TLS)
Rev1
pol ζ
pol η
pol ι
pol κ
pol ν
BRCA2
FANCN
Rad51C
Rad51
BRCA1
FANCJ
FAN1
MLH1
PMS2
Mismatch repair
Homologous recombination
Eukaryotic type
Grobal genome repair (GGR)
Transcription coupled repair (TCR)
DNA lesion
RNA polymerase II
Damage recognition
UV-DDB complex
XPC complex
CSA complex
RBX1
Cul4
DDB1
DDB2
XPC
RAD23
CETN2
CSB
CSA
UVSSA
POLR2
RNA polymerase
Ubiquitin mediated proteolysis
Centrin2
DNA unwinding
Holo-TFIIH complex
CDK7
XPB
TFIIH1
MNAT1
XPD
TFIIH2
CCNH
TTDA
TFIIH3
TFIIH4
XPG
XPA
TFIIH complex
Incision
Ubiquitin ligase
Helicase ATPase
TFIIH stabilization
CAK subcomplex
TFIIH core complex
Excision and DNA synthesis
Polδ
Polε
PCNA
RFC
DNA polymerase δ/ε
Ligation
Lig1
Lig I

I

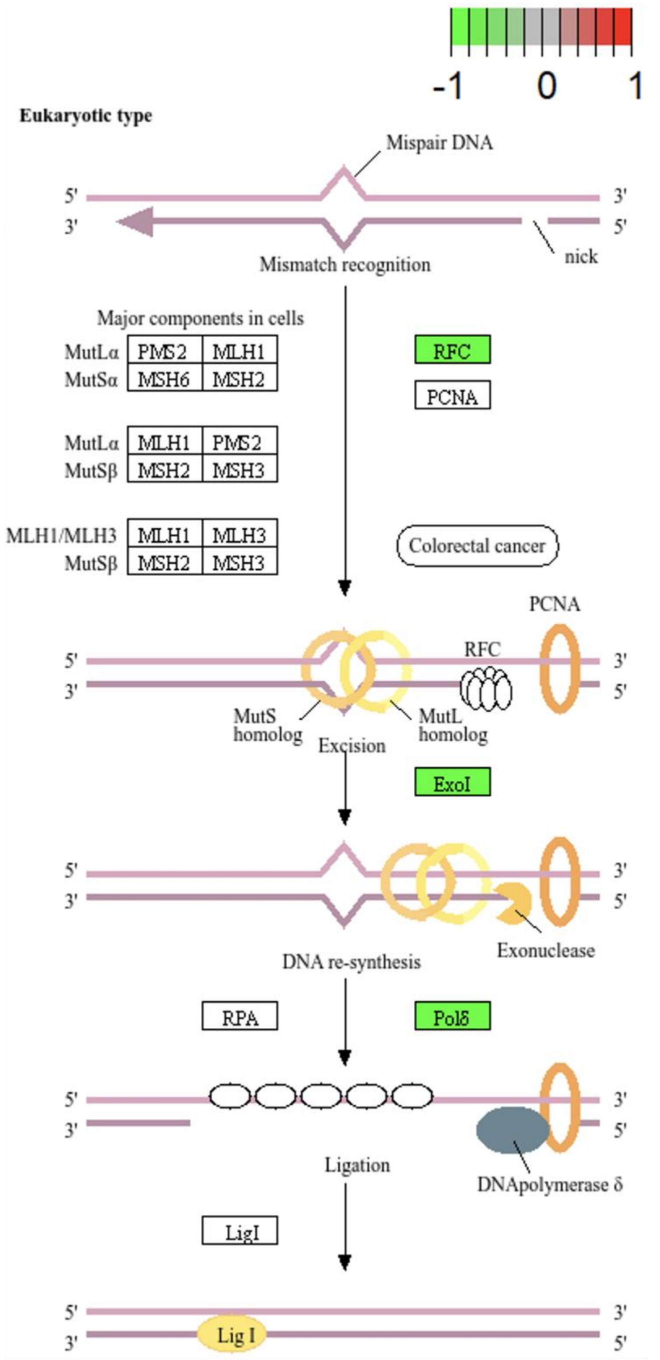

-1
0
1
Eukaryotic type
Mispair DNA
5'
3'
3'
5'
nick
Mismatch recognition
Major components in cells
MutLα PMS2 MLH1
MutSα MSH6 MSH2
MutLα MLH1 PMS2
MutSβ MSH2 MSH3
MLH1/MLH3 MLH1 MLH3
MutSβ MSH2 MSH3
RFC
PCNA
Colorectal cancer
PCNA
RFC
MutS homolog
MutL homolog
Excision
ExoI
Exonuclease
DNA re-synthesis
RPA
Polδ
Ligation
DNApolymerase δ
LigI
Lig I

J

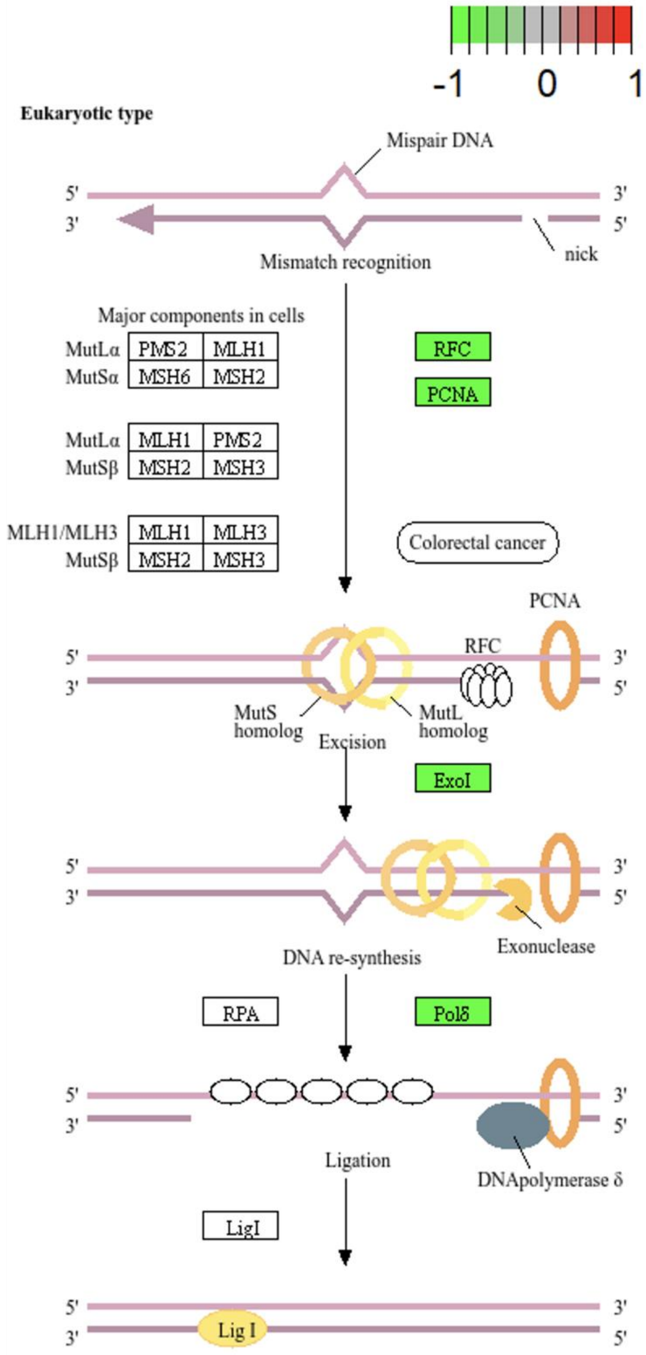

-1
0
1
Eukaryotic type
Mispair DNA
5'
3'
3'
5'
nick
Mismatch recognition
Major components in cells
MutLα PMS2 MLH1
MutSα MSH6 MSH2
MutLα MLH1 PMS2
MutSβ MSH2 MSH3
MLH1/MLH3 MLH1 MLH3
MutSβ MSH2 MSH3
RFC
PCNA
Colorectal cancer
PCNA
RFC
MutS homolog
MutL homolog
Excision
ExoI
Exonuclease
DNA re-synthesis
RPA
Polδ
Ligation
DNApolymerase δ
LigI
Lig I

Supplementary Figure S12. Expression changes of genes involved in different DNA repair pathways.

(A, B) Genes involved in homologous recombination in MCF7 *(A)* and T47D *(B)* TIS cells. Genes downregulated compared to CTR cells are highlighted in green, while upregulated genes are shown in red.

(C, D) Genes involved in base excision repair in MCF7 *(C)* and T47D *(D)* TIS cells.

(E, F) Genes involved in the Fanconi anemia pathway in MCF7 *(E)* and T47D *(F)* TIS cells.

(G, H) Genes involved in nucleotide excision repair in MCF7 *(G)* and T47D *(H)* TIS cells.

(I, J) Genes involved in mismatch repair in MCF7 *(I)* and T47D *(J)* TIS cells.

Pathway visualizations and the depiction of up- and downregulated genes were rendered using Pathview.

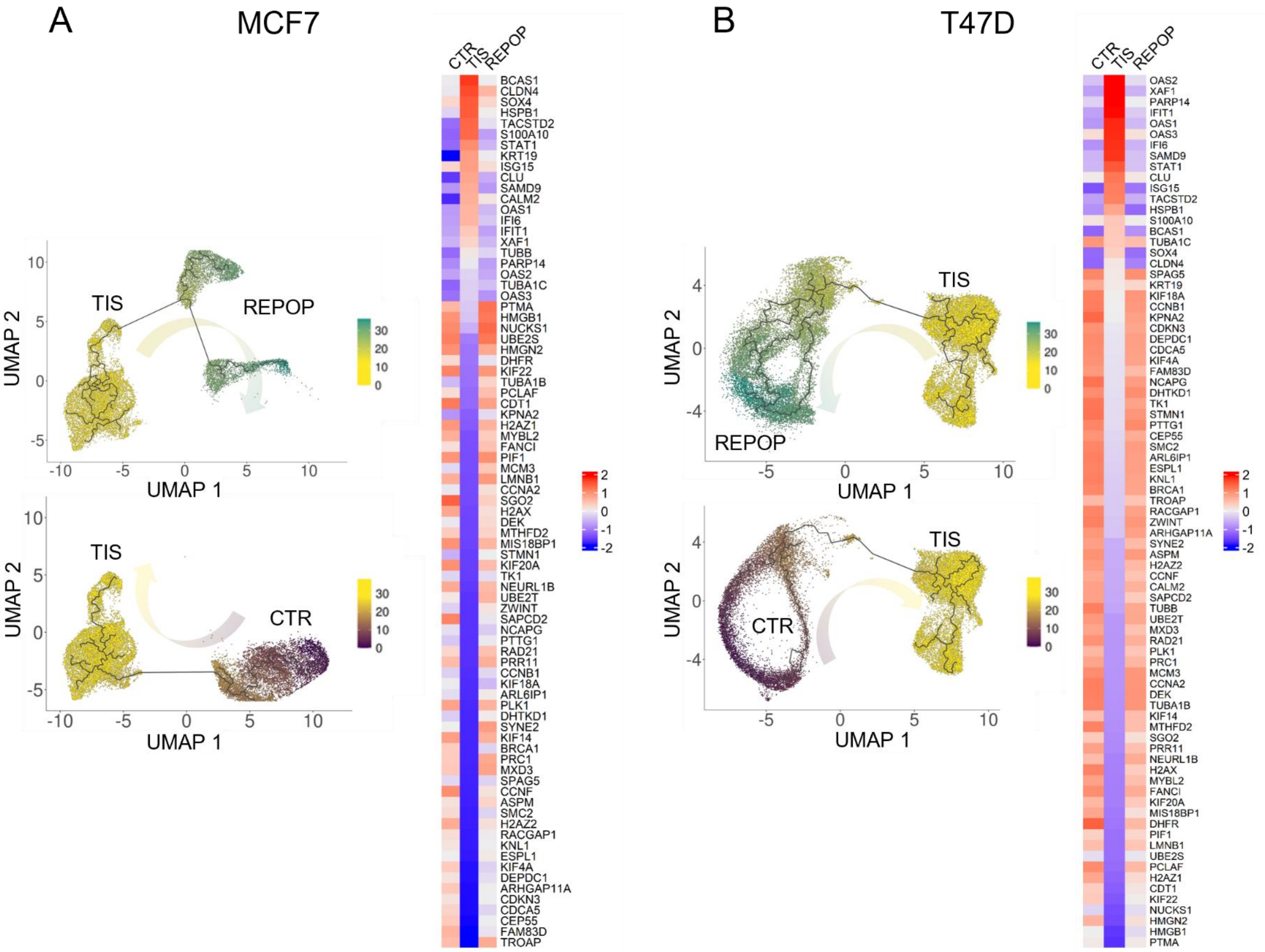

Supplementary Figure S13. Trajectory analysis of CTR, TIS, and REPOP populations excluding genes involved in cell cycle regulation.

(A, B) UMAP visualizations of trajectory analyses for MCF7 *(A)* and T47D *(B)* cells, excluding cell cycle-related genes. The top panels depict the transition trajectory from control (CTR) to the senescent (TIS) state, while the bottom panels illustrate the trajectory from the senescent (TIS) state to the repopulating (REPOP) state. The heatmap on the right displays 93 overlapping genes identified among the top 200 hits from both MCF7 and T47D trajectory analyses, highlighting common transcriptional changes associated with these transitions.

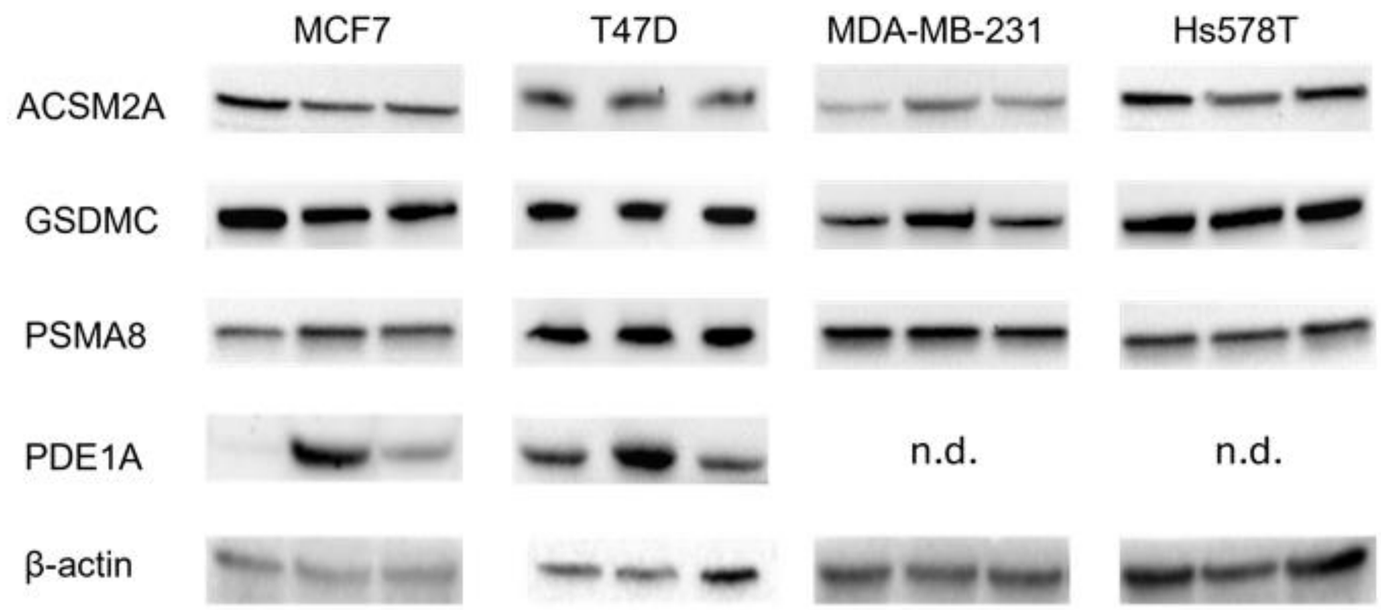

Supplementary Figure S14. Western blot analysis of ACSM2A, GSDMC, PSMA8, and PDE1A protein expression across breast cancer cell lines (MCF7, T47D, MDA-MB-231, and Hs578T) following therapy-induced senescence (TIS) induction. β-actin was used as a loading control.

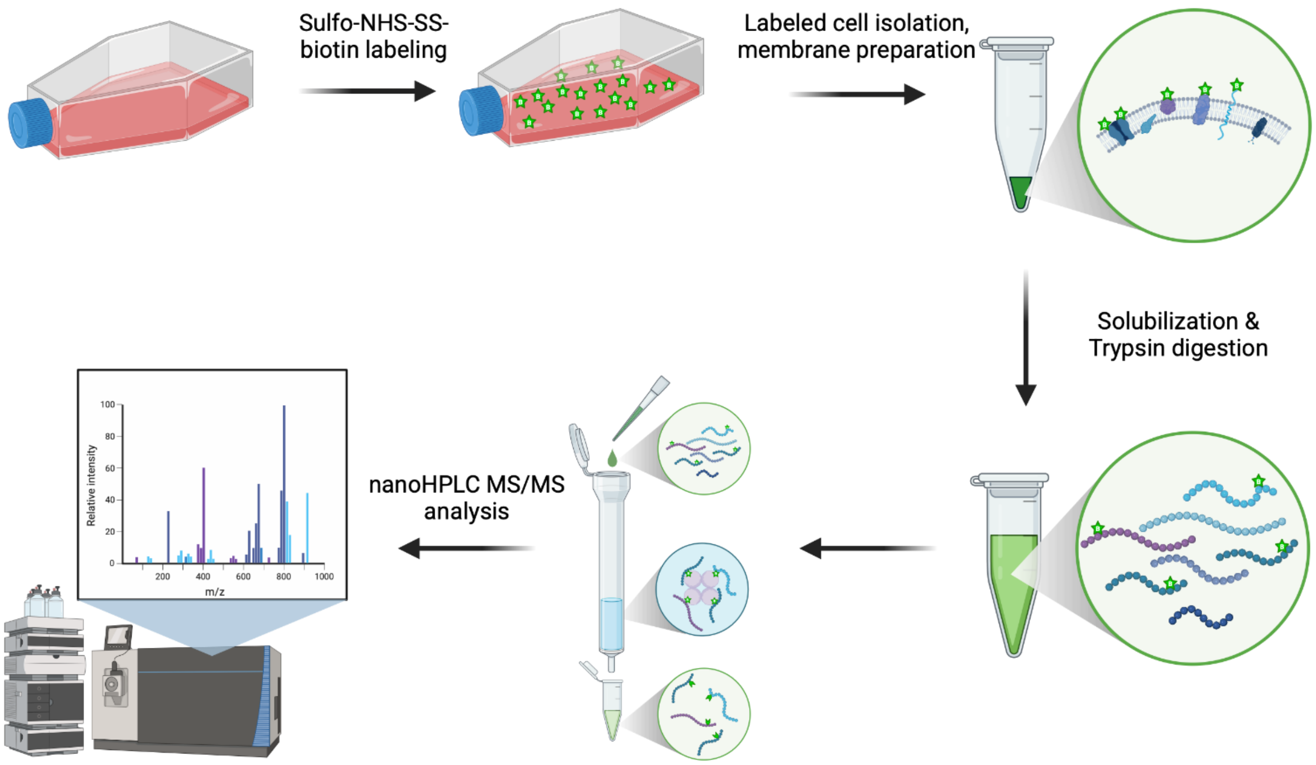

Supplementary Figure S15. Flowchart to identify the cell surface proteins. Stage-specific cells were labeled with a membrane-impermeable, primary amino group-specific labeling agent (Sulfo-NHS-SS-biotin, green stars) at room temperature (RT) for 20 minutes. The reaction was stopped with TBS buffer, cells were lysed; labeled plasma membranes were isolated, then solubilized and digested with trypsin. The biotinylated peptides were immobilized on a neutravidin agarose resin, then eluted with DTT reducing agent, desalted on a C_{18} column and sequenced by nanoHPLC-MS/MS. The surface accessible protein segments and protein abundances are determined in the Parental/TIS/Repopulated MCF7 cells. The green stars represent the Sulfo-NHS-SS-biotin labeling.

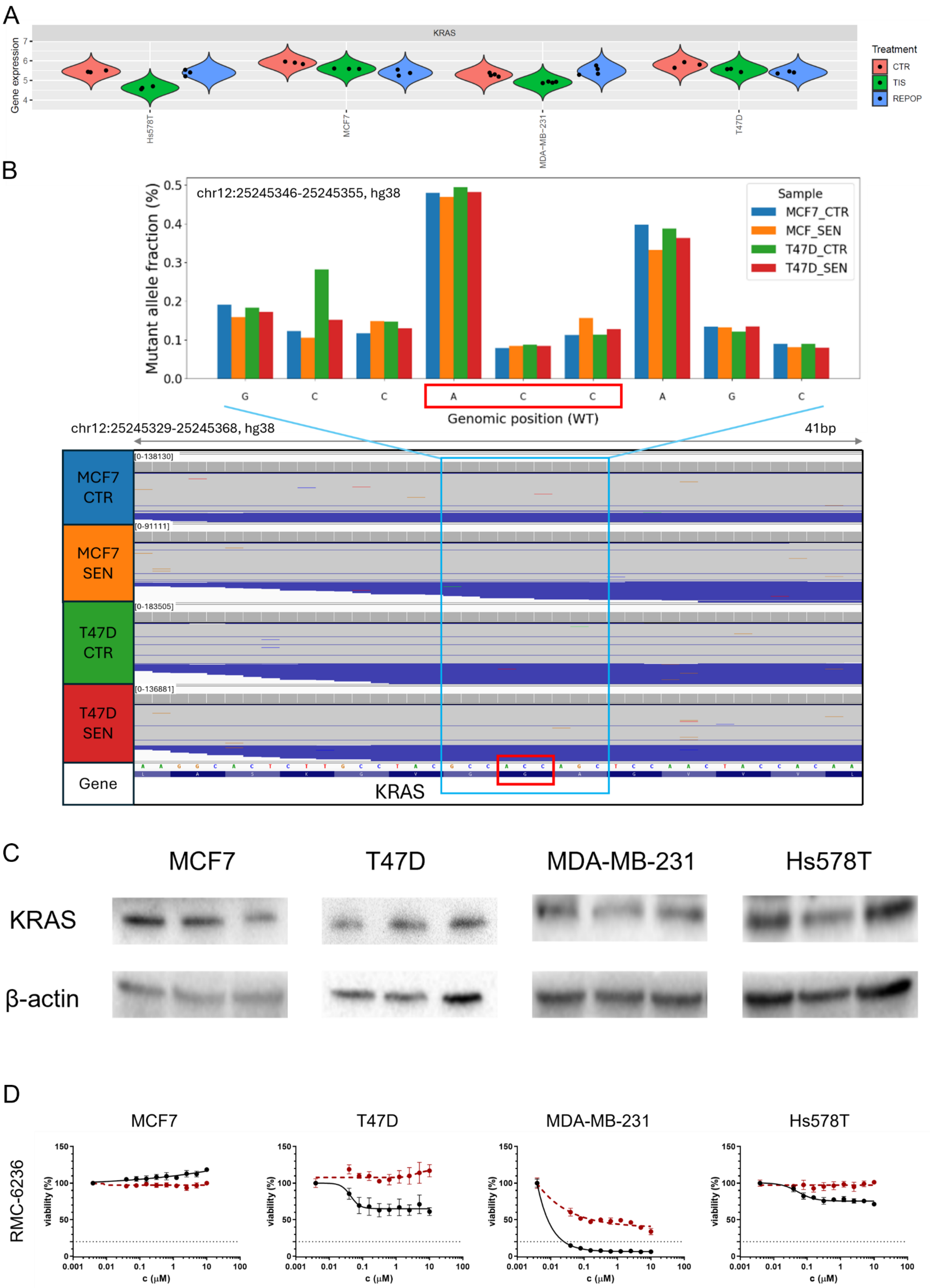
A
KRAS
Gene expression
Hs578T
MCF7
MDA-MB-231
T47D
Treatment
CTR
TIS
REPOP
B
chr12:25245346-25245355, hg38
Mutant allele fraction (%)
Sample
MCF7_CTR
MCF_SEN
T47D_CTR
T47D_SEN
G C C A C C A G C
Genomic position (WT)
chr12:25245329-25245368, hg38
41bp
MCF7 CTR
[0-138130]
MCF7 SEN
[0-91111]
T47D CTR
[0-183505]
T47D SEN
[0-136881]
Gene
KRAS
C
MCF7
T47D
MDA-MB-231
Hs578T
KRAS
β-actin
D
MCF7
T47D
MDA-MB-231
Hs578T
RMC-6236
viability (%)
c (μM)

Supplementary Figure S16. KRAS protein expression, drug sensitivity, and mutational analysis in TIS breast cancer cells.

(A) Violin plot shows normalized *KRAS* mRNA expression levels in MCF7, T47D, MDA-MB-231, and Hs578T breast cancer cell lines.

(B) Western blot analysis of KRAS protein expression in MCF7, T47D, MDA-MB-231, and Hs578T breast cancer cell lines. β-actin was used as a loading control.

C) Mutant allele fraction (%) detected in the KRAS hotspot mutation region across two breast cancer cell lines, MCF7 and T47D, under control (CTR) and senescent (SEN) conditions. Deep sequencing data revealed no significant differences between the conditions, with all detected variants occurring at extremely low frequencies (<0.1%). The red rectangle highlights the most frequent KRAS mutant genomic positions, including codon 12 mutations (e.g., G12C; c.34G>T, p.Gly12Cys). Mutant allele fraction (%) was calculated as (ALT reads / (ALT + WT reads)) × 100. The genomic positions in the upper panel correspond to **9 nucleotides (chr12:25245346-25245355, hg38)**.

(D) Integrative Genomics Viewer (IGV) tracks displaying sequencing coverage and detected variants in the same KRAS region. The sequencing data confirm the absence of condition-specific mutations, suggesting that the observed variations are most likely due to sequencing noise and stochastic fluctuations rather than biologically relevant alterations. The blue lines connect the genomic positions of interest between the panels. The lower panel displays a broader genomic region, spanning **41 nucleotides (chr12:25245329-25245368, hg38)**.

(E) Dose-response curves for RMC-6236, a pan-KRAS inhibitor, in CTR (black) and TIS (red) cells.

A

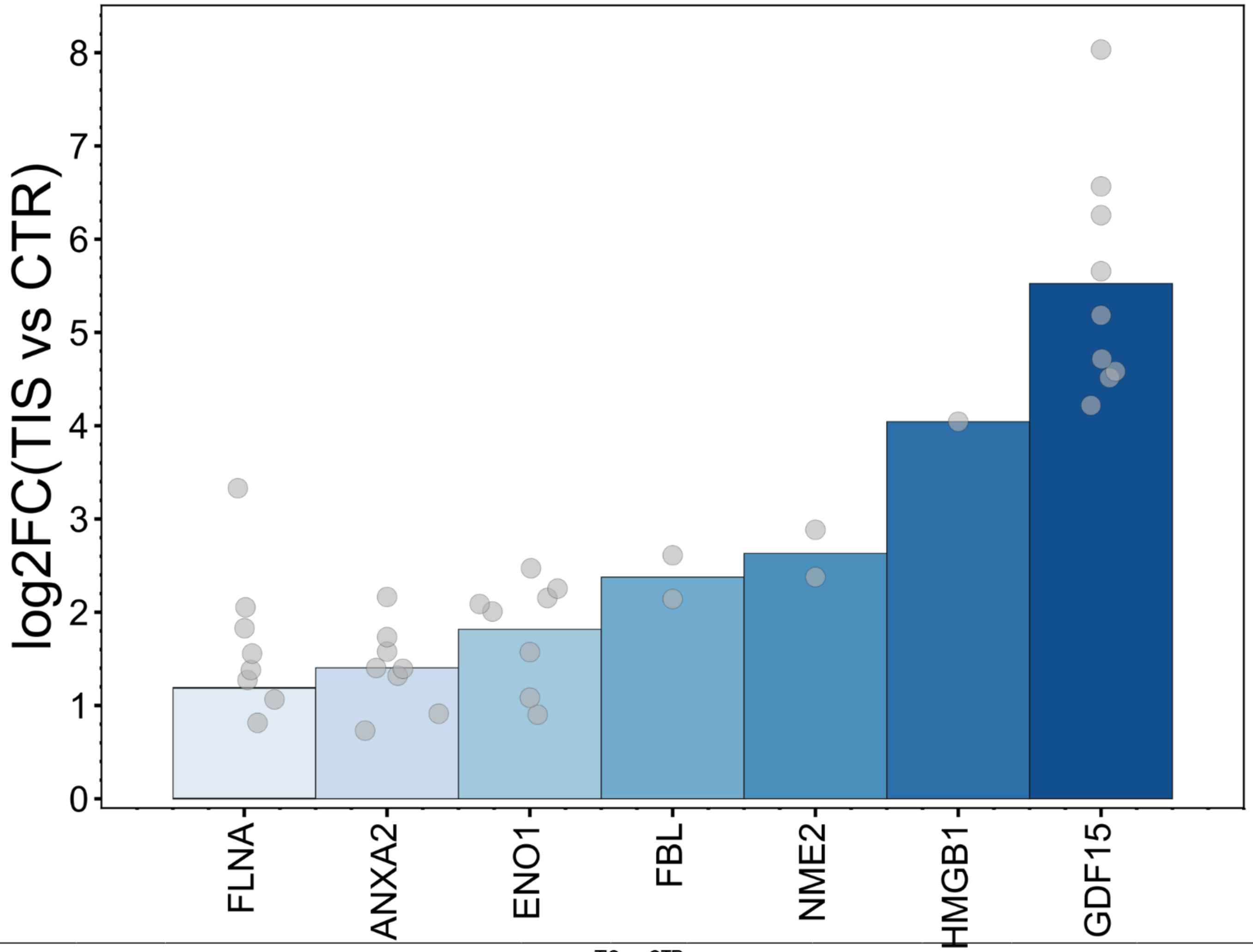

B

TIS vs CTR									
Protein			Total peptides		Bioinylated	Protein level		Peptide level	
Protein ID	Gene	Protein Description	CTR	TIS	Peptides	q-value	log2FC	Significant	Median log2FC
P07355	ANXA2	Annexin A2	26	23	14	0,00075525	1,36769	8	1,398
P06733	ENO1	Alpha-enolase;phosphopyruvate hydratase	14	27	19	0,00135417	1,86241	8	2,04767
P22087	FBL	rRNA 2'-O-methyltransferase fibrillarin	4	20	16	0,00121547	3,62619	2	2,3763
P21333	FLNA	Filamin A;Filamin-A	30	52	24	0,00158952	1,12099	13	1,06482
Q99988	GDF15	Growth/differentiation factor 15	9	71	23	0	6,14864	9	5,18416
P09429	HMGB1	High mobility group protein B1	7	47	42	0,00021505	4,21458	1	4,04352
P22392	NME2	Nucleoside diphosphate kinase B	3	6	3	0	2,42693	2	2,63034

TIS vs REPOP									
Protein			Total peptides		Bioinylated	Protein level		Peptide level	
Protein ID	Gene	Protein Description	TIS	REPOP	Peptides	q-value	log2FC	Significant	Median log2FC
P07355	ANXA2	Annexin A2	23	17	14	0,0004918	1,43996	7	1,44603
P06733	ENO1	Alpha-enolase;phosphopyruvate hydratase	27	14	19	0,0131572	1,3447	7	1,7206
P22087	FBL	rRNA 2'-O-methyltransferase fibrillarin	20	3	16	0	4,03434	2	2,17858
P21333	FLNA	Filamin A;Filamin-A	52	24	24	0	2,16553	8	1,96366
Q99988	GDF15	Growth/differentiation factor 15	71	11	23	0	5,07606	12	4,13933
P09429	HMGB1	High mobility group protein B1	47	29	42	0,00351781	1,35286	10	1,58163
P22392	NME2	Nucleoside diphosphate kinase B	6	3	3	0,00027273	1,85008	2	1,66623

REPOP vs CTR									
Protein			Total peptides		Bioinylated	Protein level		Peptide level	
Protein ID	Gene	Protein Description	CTR	REPOP	Peptides	q-value	log2FC	Significant	Median log2FC
P07355	ANXA2	Annexin A2	26	17	14	0,86039	-0,07227	1	-0,607377
P06733	ENO1	Alpha-enolase;phosphopyruvate hydratase	14	14	19	0,447176	0,517707	1	-0,702771
P22087	FBL	rRNA 2'-O-methyltransferase fibrillarin	4	3	16	0,766651	-0,40815	0	
P21333	FLNA	Filamin A;Filamin-A	30	24	24	0,0177021	-1,04453	2	-0,634064
Q99988	GDF15	Growth/differentiation factor 15	9	11	23	0,24247	1,07257	3	2,91945
P09429	HMGB1	High mobility group protein B1	7	29	42	0,00228571	2,86172	2	2,00316
P22392	NME2	Nucleoside diphosphate kinase B	3	3	3	0,157329	0,576849	1	1,36935

Supplementary Figure S17. Peptide analysis of selected proteins validates proteomic results.

(A) Distribution of log2 Fold-changes of individual peptides assigned to selected SASP marker proteins, confirming protein-level changes of those proteins in MCF7 cell proteomics experiments. Data for for TIS vs CTR sample comparisons are shown.

(B) Protein- and peptide-level identification and differential expression data for selected SASP markers. Median peptide fold-changes and difference in number of observed peptides confirm the protein level changes. Number of biotinylated peptides confirm extracellular presence. Significant q values are highlighted with shaded backgrounds.

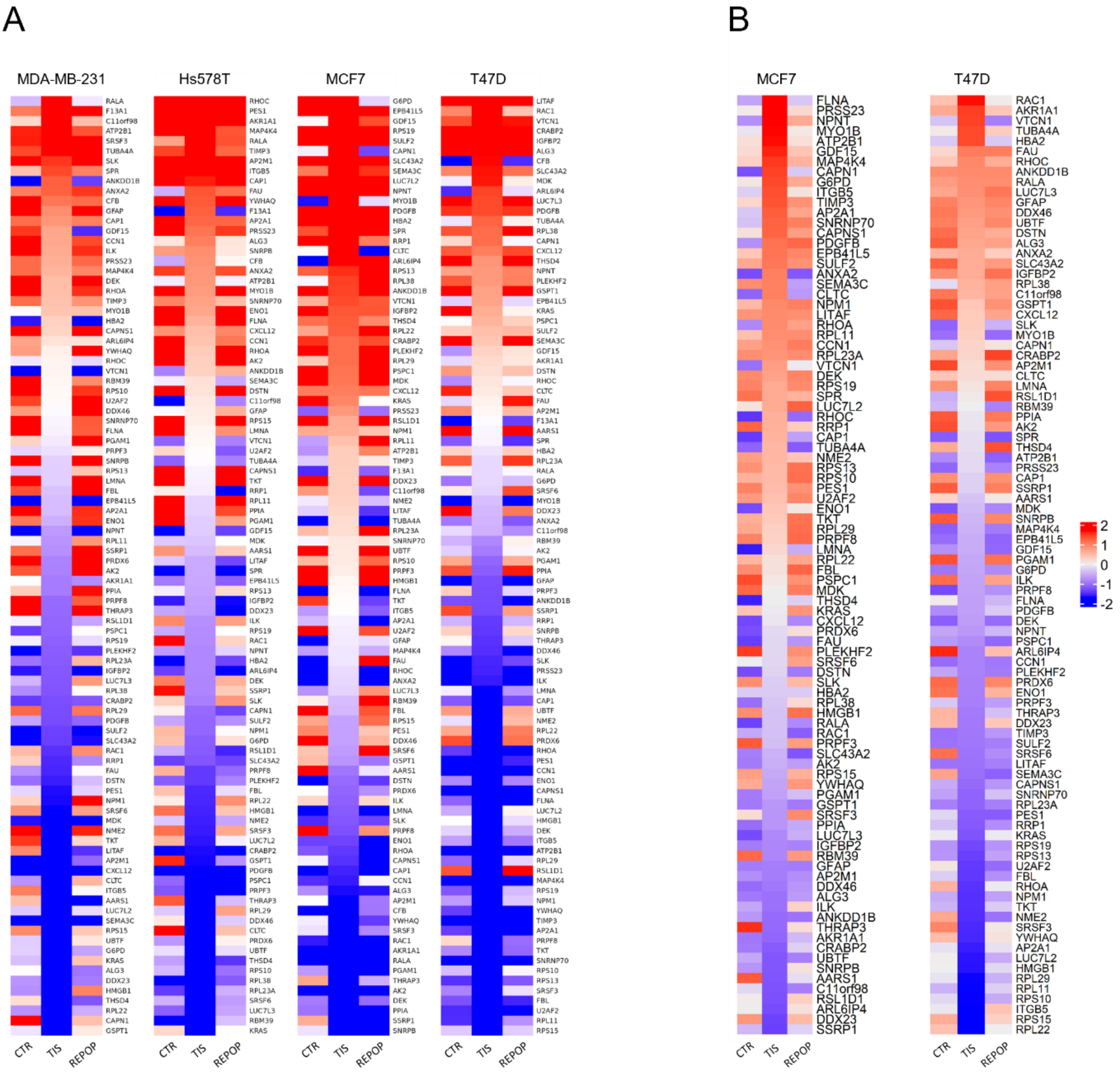
A
MDA-MB-231
Hs578T
MCF7
T47D
CTR
TIS
REPOP
B
MCF7
FLNA
PRSS23
NPNT
MYO1B
ATP2B1
GDF15
MAP4K4
CAPN1
G6PD
ITGB5
TIMP3
AP2A1
SNRNP70
CAPNS1
PDGFB
EPB41L5
SULF2
ANXA2
SEMA3C
CLTC
NPM1
LITAF
RHOA
RPL11
CCN1
RPL23A
VTCN1
DEK
RPS19
SPR
LUC7L2
RHOC
RRP1
CAP1
TUBA4A
NME2
RPS13
RPS10
PES1
U2AF2
ENO1
TKT
RPL29
PRPF8
LMNA
RPL22
FBL
PSPC1
MDK
THSD4
KRAS
CXCL12
PRDX6
FAU
PLEKHF2
SRSF6
DSTN
SLK
HBA2
RPL38
HMGB1
RALA
RAC1
PRPF3
SLC43A2
AK2
RPS15
YWHAQ
PGAM1
GSPT1
SRSF3
PPIA
LUC7L3
IGFBP2
RBM39
GFAP
AP2M1
DDX46
ALG3
ILK
ANKDD1B
THRAP3
AKR1A1
CRABP2
UBTF
SNRPB
AARS1
C11orf98
RSL1D1
ARL6IP4
DDX23
SSRP1
T47D
RAC1
AKR1A1
VTCN1
TUBA4A
HBA2
FAU
RHOC
ANKDD1B
RALA
LUC7L3
GFAP
DDX46
UBTF
DSTN
ALG3
ANXA2
SLC43A2
IGFBP2
RPL38
C11orf98
GSPT1
CXCL12
SLK
MYO1B
CAPN1
CRABP2
AP2M1
CLTC
LMNA
RSL1D1
RBM39
PPIA
AK2
SPR
THSD4
ATP2B1
PRSS23
CAP1
SSRP1
AARS1
MDK
SNRPB
MAP4K4
EPB41L5
GDF15
PGAM1
G6PD
ILK
PRPF8
FLNA
PDGFB
DEK
NPNT
PSPC1
ARL6IP4
CCN1
PLEKHF2
PRDX6
ENO1
PRPF3
THRAP3
DDX23
TIMP3
SULF2
SRSF6
LITAF
SEMA3C
CAPNS1
SNRNP70
RPL23A
PES1
RRP1
KRAS
RPS19
RPS13
U2AF2
FBL
RHOA
NPM1
TKT
NME2
SRSF3
YWHAQ
AP2A1
LUC7L2
HMGB1
RPL29
RPL11
RPS10
ITGB5
RPS15
RPL22
2
1
0
-1
-2

Supplementary Figure S18. Expression profile of 95 genes encoding proteins overexpressed exclusively on the surface of TIS cells.

(A) Gene expression levels of the 95 surface protein-coding genes identified as overexpressed exclusively in TIS cells, based on bulk RNA-seq data.

(B) Gene expression levels of the same 95 genes analyzed using scRNA-seq data, illustrating single-cell resolution of their expression profiles.

Supplementary Table S1. RT-PCR primer sequences.

Gene Symbol	Gene Name	Target Exon	Forward Sequence (5' -> 3')	Reverse Sequence (5' -> 3')	Amplicon Size (bp)
ACTB	Beta-actin	(3, 4)	CAACCGCGAGAAGATGACCC	GAGGCGTACAGGGATAGCAC	92
ACSM2A	Acyl-CoA synthetase medium-chain family member 2A	(5, 6)	CTGTGATGGGTGGCTGAACT	CAGATGCTTCCTGGCTTCCA	89
GSDMC	Gasdermin C	(2, 3)	CTCAATGACATCCTGGAGCCA	GGAACGGTCCTGTCACAACA	61
KRT6A	Keratin 6A	(1, 2)	GCTGAGGAGCGTGAACAGAT	CTTGTTCTGCTGCTCCAGGA	87
PDE1A	Phosphodiesterase 1A	(2, 3)	ATTGAATATGCGGCATCTGTG	CCGGACTTCAGATGGGACTG	117
PSMA8	Proteasome subunit alpha type-8	(4, 5)	CCTTCTGGTACTTATCATGCTTGG	ACAGTTTTAGCACTTCGGCCT	59
LMNB1	Lamin B1	(1, 2)	CAAGTGCAAGGCGGAACAC	TCTCGAAGCTTGATCTGGGC	87
CDKN1A	Cyclin-dependent kinase inhibitor 1A (p21)	(2)	CTGCCCAAGCTCTACCTTCC	CTGCCTCCTCCCAACTCATC	61
BCL2	B-cell lymphoma 2	(1, 2)	GAACTGGGGGAGGATTGTGG	GACGCTCTCCACACACATGA	60
BCL2L1	Bcl-2-like protein 1	(2)	ACATCCCAGCTCCACATCAC	CGACCCCAGTTTACCCCATC	91
MKI67	Marker of proliferation Ki-67	(4, 5)	CCTGTACGGCTAAAACATGGAG	TGACTTCCTTCCATTCTGAAGACT	90
ITGB6	Integrin subunit beta 6	(6, 7)	GACGGGCTCTGTCACTTGG	GCGAAGATCAATAACACGTTGT	119

Supplementary Table S2. IC50 values of FDA-approved drugs measured on CTR, TIS, REPOP and re-TIS breast cancer cells. The mean and standard deviation (SD) of IC50 values were calculated based on results from at least three independent experiments for each condition.

		MCF7				T47D			
		CTR	TIS	REPOP	re-TIS	CTR	TIS	REPOP	re-TIS
1	Doxorubicin	44.99 nM ± 25.85	2.11 µM ± 0.67	113.84 nM ± 119.80	5.65 µM ± 5.72	11.53 nM ± 6.42	0.93 µM ± 0.20	15.32 nM ± 5.26	0.54 µM ± 0.07

		MDA-MB-231				Hs578T			
		CTR	TIS	REPOP	re-TIS	CTR	TIS	REPOP	re-TIS
1	Doxorubicin	13.53 nM ± 5.05	0.31 µM ± 0.12	26.28 nM ± 23.19	0.26 µM ± 0.11	26.53 nM ± 21.38	1.00 µM ± 0.24	19.43 nM ± 11.50	1.18 µM ± 0.42

		MCF7				T47D			
		CTR	TIS	REPOP	re-TIS	CTR	TIS	REPOP	re-TIS
1	5-fluoro-2'-deoxycytidine	~ 50 µM	> 100 µM	~ 50 µM	> 100 µM	~ 50 µM	> 100 µM	~ 50 µM	> 100 µM
2	Bisantrene	0.52 µM ± 0.18	7.88 µM ± 1.26	0.73 µM ± 0.26	6.38 µM ± 4.18	0.21 µM ± 0.08	3.22 µM ± 0.77	0.43 µM ± 0.05	6.04 µM ± 3.16
3	Chlormethine	1.79 µM ± 1.46	13.49 µM ± 5.36	2.33 µM ± 1.29	18.41 µM ± 3.16	0.34 µM ± 0.09	2.00 µM ± 0.17	0.99 µM ± 0.73	4.55 µM ± 4.06
4	Cladribine	2.46 µM ± 0.81	> 100 µM	1.90 µM ± 0.32	> 100 µM	2.63 µM ± 0.27	> 100 µM	3.11 µM ± 0.51	83.88 µM ± 12.87
5	Clofarabine	1.17 µM ± 0.65	> 100 µM	2.07 µM ± 1.29	> 100 µM	2.77 µM ± 1.31	> 100 µM	3.94 µM ± 3.60	> 100 µM
6	Cytarabine	63.58 nM ± 3.07	> 100 µM	70.68 nM ± 43.29	> 100 µM	0.34 µM ± 0.10	> 100 µM	0.48 µM ± 0.17	> 100 µM
7	Gilteritinib	1.61 µM ± 1.41	45.59 µM ± 20.00	1.60 µM ± 1.09	32.25 µM ± 18.81	3.75 µM ± 1.76	12.50 µM ± 1.91	1.68 µM ± 1.07	14.04 µM ± 2.51
8	Mitoxantrone	0.14 µM ± 0.09	7.64 µM ± 0.75	74.09 nM ± 4.85	12.78 µM ± 3.73	62.76 nM ± 5.09	3.53 µM ± 3.06	83.37 nM ± 15.73	1.54 µM ± 0.31
9	Pevonedistat	0.32 µM ± 0.25	> 100 µM	0.39 µM ± 0.17	> 100 µM	63.21 nM ± 6.52	6.84 µM ± 2.56	104.35 nM ± 32.52	8.39 µM ± 4.36
10	Pixantrone	0.42 µM ± 0.39	16.35 µM ± 4.22	0.67 µM ± 0.24	16.97 µM ± 1.80	0.24 µM ± 0.08	4.82 µM ± 2.68	0.71 µM ± 0.38	4.25 µM ± 1.68
11	Troxacitabine	7.48 µM ± 2.43	> 100 µM	2.98 µM ± 1.31	> 100 µM	0.79 µM ± 0.48	> 100 µM	2.04 µM ± 0.22	> 100 µM
12	Vincristine	0.78 nM ± 0.30	0.20 µM ± 0.17	0.85 nM ± 0.16	0.57 µM ± 0.01	0.46 nM ± 0.16	0.43 µM ± 0.18	0.49 nM ± 0.08	0.16 µM ± 0.12
13	Volasertib	63.62 nM ± 7.96	1.52 µM ± 0.66	84.47 nM ± 2.91	1.05 µM ± 0.23	58.20 nM ± 1.44	2.70 µM ± 1.00	79.23 nM ± 13.99	1.13 µM ± 0.17
14	Voreloxin	2.56 µM ± 2.26	41.91 µM ± 13.02	4.25 µM ± 1.90	64.57 µM ± 22.16	1.59 µM ± 1.13	54.34 µM ± 14.33	4.00 µM ± 4.68	26.22 µM ± 34.93

		MDA-MB-231				Hs578T			
		CTR	TIS	REPOP	re-TIS	CTR	TIS	REPOP	re-TIS
1	5-fluoro-2'-deoxycytidine	0.79 µM ± 0.61	> 100 µM	0.52 µM ± 0.35	> 100 µM	~ 50 µM	> 100 µM	~ 50 µM	> 100 µM
2	Bisantrene	0.18 µM ± 0.14	2.38 µM ± 0.55	0.11 µM ± 0.01	1.27 µM ± 0.38	0.11 µM ± 0.04	5.56 µM ± 5.66	0.29 µM ± 0.05	9.72 µM ± 5.26
3	Chlormethine	0.59 µM ± 0.24	29.47 µM ± 19.63	0.88 µM ± 0.38	27.11 µM ± 11.08	6.47 µM ± 2.43	> 100 µM	8.23 µM ± 3.29	78.38 µM ± 18.28
4	Cladribine	0.15 µM ± 0.05	30.26 µM ± 39.78	0.13 µM ± 0.02	14.70 µM ± 15.20	8.29 µM ± 2.67	> 100 µM	7.71 µM ± 2.39	89.15 µM ± 5.48
5	Clofarabine	0.13 µM ± 0.02	> 100 µM	0.12 µM ± 0.03	> 100 µM	2.37 µM ± 1.58	> 100 µM	1.19 µM ± 0.70	> 100 µM
6	Cytarabine	0.17 µM ± 0.10	29.20 µM ± 24.33	0.20 µM ± 0.08	61.83 µM ± 19.01	0.19 µM ± 0.10	> 100 µM	0.13 µM ± 0.01	> 100 µM
7	Gilteritinib	0.57 µM ± 0.07	3.64 µM ± 2.02	0.49 µM ± 0.06	1.78 µM ± 0.28	1.98 µM ± 0.26	9.96 µM ± 2.75	2.16 µM ± 0.19	19.69 µM ± 13.50
8	Mitoxantrone	55.28 nM ± 17.90	0.78 µM ± 0.36	62.22 nM ± 2.35	0.36 µM ± 0.13	67.25 nM ± 4.02	0.85 µM ± 0.40	95.36 nM ± 18.81	1.92 µM ± 0.60
9	Pevonedistat	0.11 µM ± 0.03	12.65 µM ± 5.35	0.12 µM ± 0.05	4.40 µM ± 1.02	0.87 µM ± 0.79	55.70 µM ± 39.21	0.40 µM ± 0.04	21.61 µM ± 10.12
10	Pixantrone	0.20 µM ± 0.19	3.29 µM ± 1.19	0.09 µM ± 0.01	5.21 µM ± 3.00	0.18 µM ± 0.15	2.92 µM ± 1.73	0.37 µM ± 0.33	8.50 µM ± 0.62
11	Troxacitabine	0.17 µM ± 0.03	> 100 µM	0.13 µM ± 0.05	> 100 µM	6.66 µM ± 4.75	> 100 µM	6.11 µM ± 4.77	> 100 µM
12	Vincristine	0.69 nM ± 0.11	> 1 µM	0.68 nM ± 0.08	> 1 µM	1.20 nM ± 0.41	0.57 µM ± 0.40	0.87 nM ± 0.12	0.49 µM ± 0.13
13	Volasertib	57.83 nM ± 21.23	1.25 µM ± 0.21	57.43 nM ± 0.69	1.60 µM ± 0.61	61.82 nM ± 11.70	2.26 µM ± 0.77	38.50 nM ± 10.71	2.86 µM ± 0.28
14	Voreloxin	0.87 µM ± 0.44	11.17 µM ± 6.99	1.25 µM ± 1.01	8.10 µM ± 3.22	1.39 µM ± 0.91	10.48 µM ± 3.59	1.71 µM ± 1.39	6.07 µM ± 2.48

		MCF7		T47D		MDA-MB-231		Hs578T	
		CTR	TIS	CTR	TIS	CTR	TIS	CTR	TIS
1	5-Azacytidine	27.02 µM ± 9.33	> 100 µM	23.64 µM ± 4.43	80.04 µM ± 10.85	9.82 µM ± 3.71	64.91 µM ± 18.82	22.57 µM ± 13.07	72.54 µM ± 26.68
2	AT-7519	0.15 µM ± 0.09	0.31 µM ± 0.06	1.11 µM ± 0.98	1.92 µM ± 0.88	0.50 µM ± 0.18	0.59 µM ± 0.20	0.33 µM ± 0.15	0.86 µM ± 0.75
3	Belinostat	0.58 µM ± 0.32	0.47 µM ± 0.21	0.31 µM ± 0.14	0.42 µM ± 0.15	70.19 nM ± 4.27	209.63 nM ± 45.37	0.39 µM ± 0.14	0.99 µM ± 1.13
4	Bortezomib	49.07 nM ± 18.00	465.80 nM ± 380.42	12.73 nM ± 6.63	20.22 nM ± 12.44	35.48 nM ± 20.62	22.44 nM ± 11.25	16.27 nM ± 2.42	11.11 nM ± 1.78
5	Carfilzomib	0.18 µM ± 0.25	0.58 µM ± 0.37	2.79 nM ± 0.37	56.55 nM ± 36.63	1.56 nM ± 0.66	5.34 nM ± 2.45	3.19 nM ± 2.59	14.22 nM ± 9.72
6	Chlorambucil	26.83 µM ± 3.37	> 100 µM	11.37 µM ± 7.64	> 100 µM	7.58 µM ± 1.11	> 100 µM	28.48 µM ± 8.20	> 100 µM
7	Crenolanib	3.58 µM ± 1.73	29.06 µM ± 28.17	2.20 µM ± 0.38	10.99 µM ± 5.56	2.01 µM ± 0.37	7.50 µM ± 0.44	1.07 µM ± 0.45	3.63 µM ± 2.78
8	Dinaciclib	10.28 nM ± 9.59	129.92 nM ± 153.88	17.16 nM ± 18.99	130.89 nM ± 164.48	12.01 nM ± 6.85	16.90 nM ± 16.58	16.47 nM ± 17.60	326.77 nM ± 443.59
9	Duvelisib	10.40 µM ± 6.92	3.59 µM ± 0.82	3.52 µM ± 1.44	7.61 µM ± 1.46	8.40 µM ± 6.47	> 100 µM	21.28 µM ± 10.04	33.96 µM ± 9.24
10	Enasidenib	39.57 µM ± 22.68	34.95 µM ± 16.60	12.36 µM ± 2.67	22.00 µM ± 5.91	17.48 µM ± 6.65	65.10 µM ± 19.89	35.22 µM ± 24.45	53.35 µM ± 12.64
11	Gefitinib	48.88 µM ± 17.43	70.80 µM ± 28.70	21.51 µM ± 10.42	47.59 µM ± 22.28	16.69 µM ± 0.18	16.67 µM ± 6.15	33.27 µM ± 22.15	35.04 µM ± 26.46
12	HDAC-42	0.25 µM ± 0.11	0.22 µM ± 0.02	0.26 µM ± 0.15	0.24 µM ± 0.04	55.68 nM ± 12.21	259.57 nM ± 37.97	0.46 µM ± 0.26	0.41 µM ± 0.27
13	Homoharringtonine	33.02 nM ± 27.70	113.54 nM ± 82.31	11.88 nM ± 6.49	50.15 nM ± 26.77	21.66 nM ± 2.00	70.48 nM ± 26.81	40.60 nM ± 20.82	128.17 nM ± 66.86
14	Ibrutinib	58.55 µM ± 29.18	62.71 µM ± 31.44	17.32 µM ± 5.71	36.65 µM ± 23.45	51.13 µM ± 25.16	31.18 µM ± 19.98	49.50 µM ± 23.26	44.56 µM ± 21.15
15	Idasanutlin	0.27 µM ± 0.06	16.84 µM ± 12.96	8.75 µM ± 6.41	37.25 µM ± 29.38	15.71 µM ± 10.39	17.54 µM ± 2.16	18.42 µM ± 3.38	37.63 µM ± 19.25
16	Ixazomib	0.19 µM ± 0.14	9.80 µM ± 5.88	0.25 µM ± 0.21	0.62 µM ± 0.79	86.60 nM ± 51.46	158.79 nM ± 109.93	0.11 µM ± 0.08	1.41 µM ± 1.77
17	Masitinib	17.62 µM ± 1.00	48.81 µM ± 16.61	27.35 µM ± 20.55	47.70 µM ± 18.68	12.27 µM ± 2.24	20.29 µM ± 7.59	18.42 µM ± 2.37	43.37 µM ± 25.79
18	Melphalan	10.31 µM ± 8.14	77.79 µM ± 5.11	10.74 µM ± 5.46	58.46 µM ± 27.85	4.62 µM ± 1.82	38.33 µM ± 14.75	28.04 µM ± 5.15	> 100 µM
19	Nintedanib	5.25 µM ± 5.08	17.43 µM ± 2.84	3.03 µM ± 1.11	7.89 µM ± 3.76	5.74 µM ± 2.39	9.53 µM ± 2.81	5.75 µM ± 3.07	11.62 µM ± 5.45
20	Panobinostat	15.06 nM ± 0.27	13.42 nM ± 6.61	9.28 nM ± 4.62	8.70 nM ± 4.81	3.47 nM ± 0.69	6.10 nM ± 3.94	12.45 nM ± 3.05	18.85 nM ± 7.62
21	Pracinostat	0.36 µM ± 0.12	0.76 µM ± 0.05	0.21 µM ± 0.05	0.35 µM ± 0.21	0.11 µM ± 0.05	0.16 µM ± 0.03	0.58 µM ± 0.33	0.30 µM ± 0.29
22	Quizartinib	25.19 µM ± 8.76	44.88 µM ± 33.25	29.32 µM ± 18.23	24.29 µM ± 4.29	11.40 µM ± 9.20	17.19 µM ± 14.56	9.68 µM ± 4.93	13.30 µM ± 12.50
23	Ricolinostat	4.91 µM ± 2.91	7.07 µM ± 0.74	3.23 µM ± 1.70	4.12 µM ± 2.89	2.62 µM ± 2.97	1.96 µM ± 0.28	5.51 µM ± 2.24	1.69 µM ± 1.15
24	Romidepsin	3.77 nM ± 2.25	3.00 nM ± 1.54	2.36 nM ± 0.22	4.92 nM ± 1.18	0.91 nM ± 0.09	1.64 nM ± 0.39	1.09 nM ± 0.34	1.93 nM ± 1.20
25	SB-1317	49.37 nM ± 15.04	474.50 nM ± 263.03	60.47 nM ± 16.23	326.93 nM ± 54.52	42.81 nM ± 18.12	57.93 nM ± 28.50	61.99 nM ± 10.64	174.61 nM ± 75.32
26	Selinexor	91.38 nM ± 38.37	401.60 nM ± 265.22	75.41 nM ± 3.74	707.73 nM ± 252.61	30.51 nM ± 3.52	91.24 nM ± 12.79	42.53 nM ± 6.14	526.27 nM ± 346.33
27	Sorafenib	11.69 µM ± 5.70	19.24 µM ± 6.13	7.04 µM ± 3.55	12.94 µM ± 8.13	0.94 µM ± 0.30	40.39 µM ± 33.22	9.21 µM ± 3.93	5.52 µM ± 1.94
28	Sunitinib	11.53 µM ± 1.42	14.84 µM ± 2.11	25.27 µM ± 17.58	23.69 µM ± 9.07	3.74 µM ± 1.85	5.24 µM ± 2.64	9.07 µM ± 9.09	20.27 µM ± 20.57
29	Tipifarnib	10.21 µM ± 5.23	17.07 µM ± 0.25	2.21 µM ± 1.24	13.56 µM ± 4.09	0.76 µM ± 0.30	24.45 µM ± 3.88	6.76 µM ± 1.68	31.73 µM ± 27.62
30	Venetoclax	26.28 µM ± 24.20	9.65 µM ± 5.76	20.65 µM ± 7.15	22.52 µM ± 11.03	26.38 µM ± 20.41	35.94 µM ± 19.75	12.39 µM ± 3.72	14.52 µM ± 6.53
31	Vorinostat	1.61 µM ± 0.31	1.79 µM ± 0.75	0.87 µM ± 0.19	1.45 µM ± 0.43	0.32 µM ± 0.15	1.35 µM ± 0.52	1.07 µM ± 0.22	1.64 µM ± 0.53
32	Zosuquidar	40.86 µM ± 15.43	13.10 µM ± 9.33	12.33 µM ± 2.91	13.63 µM ± 9.67	19.08 µM ± 8.57	17.28 µM ± 8.18	15.91 µM ± 2.94	12.52 µM ± 3.96

		MCF7				T47D			
		CTR	TIS	REPOP	re-TIS	CTR	TIS	REPOP	re-TIS
1	Gemcitabine	< 0.1 µM	> 100 µM	< 0.1 µM	> 100 µM	< 0.1 µM	> 100 µM	< 0.1 µM	> 100 µM
2	Paclitaxel	< 50 nM	17.25 µM ± 10.73	< 50 nM	26.34 µM ± 10.36	< 50 nM	11.12 µM ± 1.71	< 50 nM	17.68 µM ± 14.41
3	Docetaxel	< 50 nM	5.46 µM ± 3.08	< 50 nM	12.77 µM ± 3.62	< 50 nM	8.13 µM ± 4.53	< 50 nM	6.04 µM ± 5.46

		MDA-MB-231				Hs578T			
		CTR	TIS	REPOP	re-TIS	CTR	TIS	REPOP	re-TIS
1	Gemcitabine	< 0.1 µM	3.03 µM ± 1.68	< 0.1 µM	1.57 µM ± 0.81	< 0.1 µM	> 100 µM	< 0.1 µM	> 100 µM
2	Paclitaxel	< 50 nM	17.31 µM ± 8.82	< 50 nM	21.66 µM ± 12.27	< 50 nM	18.01 µM ± 12.29	< 50 nM	22.32 µM ± 4.28
3	Docetaxel	< 50 nM	6.11 µM ± 2.77	< 50 nM	8.13 µM ± 2.99	< 50 nM	7.76 µM ± 2.26	< 50 nM	8.57 µM ± 2.87

Supplementary Table S3. IC50 values of senolytic compounds measured on CTR, TIS, REPOP and re-TIS breast cancer cells. The mean and standard deviation (SD) of IC50 values were calculated based on results from at least three independent experiments for each condition.

		MCF7				T47D			
		CTR	TIS	REPOP	re-TIS	CTR	TIS	REPOP	re-TIS
1	Navitoclax	13.56 µM ± 3.81	3.68 µM ± 1.63	10.55 µM ± 3.20	1.35 µM ± 0.51	13.67 µM ± 5.72	3.72 µM ± 2.22	10.92 µM ± 6.28	4.21 µM ± 1.87

		MDA-MB-231				Hs578T			
		CTR	TIS	REPOP	re-TIS	CTR	TIS	REPOP	re-TIS
1	Navitoclax	3.23 µM ± 1.89	0.12 µM ± 0.13	1.77 µM ± 0.97	0.10 µM ± 0.06	9.90 µM ± 5.62	0.88 µM ± 0.42	7.63 µM ± 5.42	0.91 µM ± 0.54

		MCF7		T47D		MDA-MB-231		Hs578T	
		CTR	TIS	CTR	TIS	CTR	TIS	CTR	TIS
1	ABT-737	14.49 µM ± 7.00	4.55 µM ± 0.90	10.32 µM ± 1.80	4.25 µM ± 1.55	3.74 µM ± 0.61	0.38 µM ± 0.20	9.73 µM ± 1.42	3.29 µM ± 2.09
2	A-1331852	29.04 µM ± 2.23	6.87 µM ± 6.22	26.16 µM ± 9.33	0.49 µM ± 0.62	0.16 µM ± 0.09	< 7.62 nM	4.31 µM ± 1.85	0.01 µM ± 0.01
3	Piperlongumine	2.76 µM ± 1.22	13.76 µM ± 0.37	2.04 µM ± 0.15	9.51 µM ± 3.40	1.53 µM ± 0.52	3.04 µM ± 0.63	2.64 µM ± 0.21	6.17 µM ± 4.30
4	Dasatinib	24.80 µM ± 4.80	> 50 µM	32.47 µM ± 12.42	47.23 µM ± 2.34	0.01 µM ± 0.00	0.81 µM ± 0.78	0.09 µM ± 0.04	1.26 µM ± 1.15
5	Quercetin	20.00 µM ± 9.44	> 50 µM	31.61 µM ± 13.44	> 50 µM	12.40 µM ± 8.32	> 50 µM	26.03 µM ± 14.85	40.23 µM ± 13.82
6	Fisetin	24.95 µM ± 11.76	> 50 µM	35.23 µM ± 10.59	> 50 µM	14.28 µM ± 8.46	> 50 µM	23.04 µM ± 11.47	36.87 µM ± 11.36

Supplementary Table S4. Expression data of the 22 genes overexpressed in TIS and REPOP cells but not in CTR. This table presents the expression levels of 22 genes that are consistently overexpressed in both TIS and REPOP cells while showing no significant expression in CTR cells. The data highlights potential genes involved in maintaining features unique to the senescent and repopulating states.

gene name	MCF7 ctr to sen logFC	MCF7 ctr to sen adj.P.Val	MCF7 ctr to repop logFC	MCF7 ctr to repop adj.P.Val	T47D ctr to sen logFC	T47D ctr to sen adj.P.Val	T47D ctr to repop logFC	T47D ctr to repop adj.P.Val	Hs578T ctr to sen logFC	Hs578T ctr to sen adj.P.Val	Hs578T ctr to repop logFC	Hs578T ctr to repop adj.P.Val	MDA-MB-231 ctr to sen logFC	MDA-MB-231 ctr to sen adj.P.Val	MDA-MB-231 ctr to repop logFC	MDA-MB-231 ctr to repop adj.P.Val
**IFIT1**	4.856463292	3.15753E-12	3.593045972	5.87604E-09	7.795487514	4.97582E-17	2.864282244	6.54674E-06	3.254137344	1.60116E-08	1.921749705	0.000678229	2.313881397	3.46958E-07	1.094629942	0.015723944
**IFIT3**	3.809210136	2.47336E-10	2.964121585	9.44168E-08	7.804800179	1.62112E-17	2.199028057	0.000183134	3.442453861	2.08617E-09	1.287750633	0.015515944	1.687496674	2.49784E-05	0.376664885	0.471695097
**OAS1**	4.909928108	1.04848E-11	3.606516989	1.87352E-08	6.115042852	1.08321E-13	2.540130102	0.000111785	7.026822261	3.973E-15	4.855006839	5.06091E-10	3.453411034	5.07097E-10	0.277337254	0.679210817
**SYTL2**	1.791915849	1.13077E-08	1.943784599	5.25382E-09	4.197660848	4.97582E-17	2.052053159	3.09782E-08	-	-	-	-	1.514573205	8.88578E-09	1.110656586	0.000023542
**IFIT2**	3.820105412	2.83715E-11	2.873074893	2.77096E-08	6.721851941	5.31616E-17	1.692946364	0.001424041	4.074179289	9.43979E-12	1.341099476	0.005544607	1.974988718	4.61764E-07	0.339185804	0.477546701
**RTP4**	5.192438697	4.47732E-11	4.38096072	4.48386E-09	8.625350627	4.4676E-16	2.634139086	0.000349478	2.069271651	0.000294994	1.528401773	0.022985627	3.216593299	2.36617E-08	0.886871255	0.117785133
**OASL**	2.149831766	9.61232E-07	1.886623185	1.44265E-05	7.241745383	4.01643E-18	2.554176019	1.45898E-06	4.864080987	4.56511E-14	2.417408181	1.91231E-06	3.248720192	6.34844E-12	0.55337547	0.157456403
**N4BP2L1**	3.134955526	4.99224E-13	2.328313729	1.24636E-09	2.156255362	4.21256E-09	1.287207457	0.000444622	1.314858933	1.21025E-05	1.011747894	0.002462041	0.17855832	0.459392157	-1.251843418	1.97969E-05
**IFITM1**	1.283430025	0.000834298	1.814955144	1.64689E-05	4.450373745	1.84885E-13	1.361768773	0.006009335	2.139943289	4.65245E-07	2.93500191	3.78101E-08	-0.698708475	0.021993556	-0.291202661	0.511377257
**AMY2B**	4.131943985	8.41432E-11	3.438993463	1.03688E-08	5.228583575	7.65397E-13	3.175244052	7.91533E-07	2.51838808	1.14182E-06	2.145762163	0.000170158	1.027450291	0.007162839	0.097729258	0.86598609
**PARP9**	2.136476141	1.32234E-10	1.261171732	6.79277E-06	3.426435391	3.76048E-15	1.020053495	0.001348003	1.622971328	3.4805E-08	1.022692864	0.000531196	0.250561102	0.216025495	0.376429146	0.126565073
**IFI44**	3.378602034	3.79182E-08	2.204306429	8.01005E-05	8.373813718	5.23538E-17	5.537478117	9.48388E-11	3.694781162	5.8129E-09	2.317572552	0.000198643	1.966018923	1.61177E-05	0.514416007	0.35791054
**XAF1**	4.26870109	2.395E-09	5.279289988	9.51706E-11	7.500481196	1.07379E-14	2.635154517	0.000303287	2.461659128	2.80362E-05	1.900627246	0.004144265	-0.40570308	0.392706923	-1.206870674	0.025576816
**ISG15**	2.7201117	2.38083E-10	2.346487503	1.27121E-08	7.32857919	2.27412E-20	2.120133315	1.48544E-06	2.636284997	5.03715E-10	1.129451954	0.002882622	1.797424679	2.97253E-08	0.336354576	0.335367194
**CLIC5**	6.126333704	4.12677E-07	2.840653572	0.01024547	5.23348914	9.01819E-06	3.763145103	0.006714879	5.584477895	1.4793E-06	2.917761863	0.018574416	-0.065213049	0.945939939	-0.821768956	0.506979005
**IFI6**	5.03373559	1.95131E-13	3.547226874	1.629E-09	7.433421459	2.37343E-17	2.745013424	3.27662E-06	3.667308347	3.0985E-10	1.101412689	0.034048569	1.724369654	1.14404E-05	-0.368635347	0.465629975
**IFI27**	3.474026907	1.93598E-07	4.19517652	1.13351E-08	9.559072662	3.60463E-17	4.845737318	1.26696E-08	4.204250531	4.31333E-09	3.185796025	1.10189E-05	1.126696017	0.015487539	-0.990280931	0.077492355
**RSAD2**	5.015806572	4.74641E-09	3.048231926	5.23052E-05	8.569670205	4.58722E-14	3.00660222	0.000670211	8.847736741	2.28405E-14	5.965640143	4.33977E-09	7.669742964	8.60281E-15	0.543164267	0.497386273
**NR4A2**	1.065514187	0.000216557	1.950955762	2.42999E-08	1.665237422	4.13745E-07	1.487768854	4.08201E-05	1.236213951	2.13111E-05	1.609863672	5.81887E-06	-0.851398325	0.000308318	-0.685799934	0.009627482
**BLNK**	1.820489124	0.003448972	2.171723719	0.000933971	-	-	-	-	5.513391503	8.24731E-11	2.311478343	0.001572909	4.524462111	1.17228E-10	1.556508628	0.008356267
**OAS2**	4.078366087	2.50143E-09	3.792813126	2.45699E-08	5.788657079	2.14175E-12	1.827465471	0.011077096	4.732264667	1.21617E-10	3.635641962	4.91171E-07	1.34592054	0.002553685	0.203782545	0.80028441
**CMPK2**	3.945277102	2.35398E-05	2.943006265	0.001396799	7.303749804	5.49435E-10	2.531807126	0.03600038	5.005911818	3.82903E-07	2.292592756	0.025655831	5.908910567	5.70557E-10	1.160268003	0.191932108

Supplementary Table S5. IC50 values of inhibitors measured on CTR and TIS breast cancer cells. The mean and standard deviation (SD) of IC50 values were calculated based on results from at least three independent experiments for each condition.

		MCF7						T47D					
		CTR			TIS			CTR			TIS		
1	Simvastatin	25.45	μM ±	10.97	24.33	μM ±	8.96	19.52	μM ±	5.85	12.64	μM ±	2.22
2	(R)-MG132	60.96	μM ±	6.77	> 100 μM			53.91	μM ±	22.81	77.80	μM ±	10.84
3	Actinomycin D	0.65	nM ±	0.29	4.26	nM ±	0.56	0.27	nM ±	0.01	0.99	nM ±	0.28
4	Thymoquinone	21.23	μM ±	10.15	46.02	μM ±	14.75	6.42	μM ±	3.57	14.59	μM ±	12.86
5	4-Methylsalicyclic acid	> 1000 μM			> 1000 μM			> 1000 μM			> 1000 μM		
6	Dimethyl 2-oxoglutarate	> 1000 μM			> 1000 μM			> 1000 μM			> 1000 μM		

Supplementary Table S6. Proteins identified by proteomics analysis exclusively in TIS cells. *(A)* Proteins identified in TIS cells that have been previously described in connection with senescence, providing insights into known molecular players in the senescent phenotype. *(B)* Proteins identified in TIS cells that have been previously described in connection with the spliceosome, highlighting potential links between senescence and splicing regulation.

A

Gene ID	Name	PMID / doi
**AP2A1**	Adaptor Related Protein Complex 2 Subunit Alpha 1	https://doi.org/10.1101/2023.08.19.553998, PMID: 34705524
**AP2M1**	Adaptor Related Protein Complex 2 Subunit Mu 1	PMID: 29415991
**CAPNS1**	Calpain Small Subunit 1	PMID: 20107320
**CCN1**	Cellular Communication Network Factor 1	PMID: 20526329, PMID: 23508104, PMID: 32145148
**CFB**	Complement Factor B	PMID: 34075561
**CXCL12**	C-X-C Motif Chemokine Ligand 12	PMID: 39251867, PMID: 34801033, PMID: 33643790
**DEK**	DEK Proto-Oncogene	PMID: 22390170, PMID: 16254365
**F13A1**	Coagulation Factor XIII A Chain	PMID: 35906424
**GDF15**	Growth Differentiation Factor 15	PMID: 31945054,PMID: 35051643
**HMGB1**	High Mobility Group Box 1	PMID: 30474841, PMID: 23649808, PMID: 34166567, PMID: 31129019, PMID: 33558529
**IGFBP2**	Insulin Like Growth Factor Binding Protein 2	PMID: 37982669
**ILK**	Integrin Linked Kinase	PMID: 35778385, PMID: 26176204, PMID: 17234816, PMID: 29101014
**LMNA**	Lamin A/C	PMID: 30728349
**LUC7L3**	LUC7 like 3 pre-mRNA splicing factor	PMID: 38590928
**MAP4K4**	Mitogen-Activated Protein Kinase Kinase Kinase Kinase 4	PMID: 38383842, PMID: 28611026, PMID: 35224163
**MDK**	Midkine	PMID: 16229804, PMID: 37766998, PMID: 26717900
**NPM1**	Nucleophosmin 1	PMID: 23536448
**PDGFB**	Platelet Derived Growth Factor Subunit B	PMID: 23934686
**PRDX6**	Peroxiredoxin 6	PMID: 28904819, PMID: 34923300
**PRPF19**	Pre-mRNA Processing Factor 19	PMID: 34144037, PMID: 28703423
**PRPF8**	Pre-MRNA Processing Factor 8	PMID: 34144037
**PRSS23**	Serine Protease 23	PMID: 36617688
**RAC1**	Rac Family Small GTPase 1	PMID: 15024070, PMID: 27818180, PMID: 17032649
**RBM39**	RNA Binding Motif Protein 39	PMID: 34077726
**RHOA**	Ras Homolog Family Member A	PMID: 17658517, PMID: 21273559, PMID: 33361519
**RPL11**	Ribosomal Protein L11	PMID: 25732822, PMID: 37157887
**RPL22**	Ribosomal Protein L22	PMID: 39258545, PMID: 30874462
**RPL29**	Ribosomal Protein L29	PMID: 29941930
**RPS15**	Ribosomal Protein S15	PMID: 31921849
**SEMA3C**	Semaphorin 3C	PMID: 28673354
**SNRPB**	Small Nuclear Ribonucleoprotein Polypeptides B And B1	PMID: 39118304
**SRSF3**	Serine And Arginine Rich Splicing Factor 3	PMID: 22777358
**SRSF6**	Serine And Arginine Rich Splicing Factor 6	PMID: 37751047, PMID: 28703423
**SSRP1**	Structure Specific Recognition Protein 1	PMID: 23839038
**SULF2**	Sulfatase 2	PMID: 19190338, PMID: 27545311
**TIMP3**	TIMP Metallopeptidase Inhibitor 3	PMID: 25347747
**YWHAQ**	Tyrosine 3-Monooxygenase/Tryptophan 5-Monooxygenase Activation Protein Theta	PMID: 33674410

B

Gene ID	Name	PMID
**ARL6IP4**	ADP Ribosylation Factor Like GTPase 6 Interacting Protein 4	PMID: 32047265, PMID: 10708573, PMID: 11884129
**DDX23**	DEAD-Box Helicase 23	PMID: 34966670
**DDX46**	DEAD-Box Helicase 46	PMID: 36797247
**DEK**	DEK Proto-Oncogene	PMID: 35475534
**LUC7L2**	LUC7 Like 2, Pre-MRNA Splicing Factor	PMID: 33852859
**LUC7L3**	LUC7 Like 3 Pre-MRNA Splicing Factor	PMID: 38785515, PMID: 33852859
**PRPF3**	Pre-MRNA Processing Factor 3	PMID: 31926109
**PRPF8**	Pre-MRNA Processing Factor 8	PMID: 26392272
**RBM39**	RNA Binding Motif Protein 39	PMID: 34788094
**SNRNP70**	Small Nuclear Ribonucleoprotein U1 Subunit 70	PMID: 36384140
**SNRPB**	Small Nuclear Ribonucleoprotein Polypeptides B And B1	PMID: 33289700, PMID: 35593225, PMID: 37391593
**SRSF3**	Serine And Arginine Rich Splicing Factor 3	PMID: 22777358
**SRSF6**	Serine And Arginine Rich Splicing Factor 6	PMID: 32901876
**THRAP3**	Thyroid Hormone Receptor Associated Protein 3	PMID: 29112714
**U2AF2**	U2 Small Nuclear RNA Auxiliary Factor 2	PMID: 35524551

REAGENT or RESOURCE	SOURCE	IDENTIFIER
**Cell lines & Cell culture**		
MCF7	Developmental Therapeutic Program of the National Cancer Institute	CVCL_0031
T47D	Developmental Therapeutic Program of the National Cancer Institute	CVCL_0553
MDA-MB-231	Developmental Therapeutic Program of the National Cancer Institute	CVCL_0062
Hs578T	Developmental Therapeutic Program of the National Cancer Institute	CVCL_0332
HFF-1	ATCC	SCRC-1041
RPMI	Thermo Fisher Scientific	Cat# 11875093
DMEM-F12	Thermo Fisher Scientific	Cat# 10565018
FBS	Thermo Fisher Scientific	A5256701
L-glutamine	Euroclone	ECB3000D
Penicillin - Streptomycin	Capricorn Scientific	PS-B
Gentamicin	Thermo Fisher Scientific	15750045
Fibroblast Growth Factor 2	Peprotech	Cat#100-18C-50UG
**Drugs**		
(R)-MG132	Merck Life Science	M8699
4-Methylsalicyclic acid	Merck Life Science	244503
5-Azacytidine	Merck Life Science	A2385
5-fluoro-2'-deoxycytidine	Merck Life Science	F5307
A-1331852	MedChemExpress	HY-19741
ABT-737	MedChemExpress	HY-50907
Actinomycin D	Merck Life Science	A1410
Amifostine	MedChemExpress	HY-B0639
AT-7519	Selleck Chemicals LLC	S1524
Belinostat	Merck Life Science	SML3010
Bisantrene	Selleck Chemicals LLC	S9946
Bortezomib	Merck Life Science	5.04314
Carfilzomib	Selleck Chemicals LLC	S2853
Chlorambucil	Selleck Chemicals LLC	S4288
Chlormethine	MedChemExpress	HY-B1253
Cladribine	Selleck Chemicals LLC	S1199
Clofarabine	Selleck Chemicals LLC	S1218
Covidarabine	MedChemExpress	HY-B0277
Crenolanib	Selleck Chemicals LLC	S2730
Cyclophosphamide	MedChemExpress	HY-17420
Cytarabine	Selleck Chemicals LLC	S1648
Dasatinib	MedChemExpress	HY-10181
Dexamethasone	MedChemExpress	HY-14648

Dimethyl 2-oxoglutarate	Merck Life Science	349631
Dinaciclib	Selleck Chemicals LLC	S2768
Docetaxel	MedChemExpress	HY-B0011
Doxorubicin	Merck Life Science	D1515
Duvelisib	Selleck Chemicals LLC	S7028
Enasidenib	Selleck Chemicals LLC	S8205
Fisetin	MedChemExpress	HY-N0182
Gefitinib	Selleck Chemicals LLC	S1025
Gemcitabine	MedChemExpress	HY-17026
Gilteritinib	Selleck Chemicals LLC	S7754
Glasdegib	MedChemExpress	HY-16391
HDAC-42	Selleck Chemicals LLC	S2244
Histamine	MedChemExpress	HY-B1204
Homoharringtonine	Selleck Chemicals LLC	S9015
Ibrutinib	Selleck Chemicals LLC	S2680
Idasanutlin	Selleck Chemicals LLC	S7205
Ivosidenib	MedChemExpress	HY-18767
Ixazomib	Selleck Chemicals LLC	S2180
Laromustine	MedChemExpress	HY-10537
Lenalidomide	MedChemExpress	HY-A0003
Masitinib	Selleck Chemicals LLC	S1064
Meloxicam	MedChemExpress	HY-B0261
Melphalan	Selleck Chemicals LLC	S8266
Metformin	MedChemExpress	HY-B0627
Methylprednisolone	MedChemExpress	HY-B0260
Mitoxantrone	MedChemExpress	HY-13502
Navitoclax	MedChemExpress	HY-10087
Nintedanib	Selleck Chemicals LLC	S1010
Paclitaxel	MedChemExpress	HY-B0015
Panobinostat	Selleck Chemicals LLC	S1030
Pevonedistat	Selleck Chemicals LLC	S7109
Piperlongumine	MedChemExpress	HY-N2329
Pixantrone	MedChemExpress	HY-13727A
Plerixafor	MedChemExpress	HY-10046
Pracinostat	MedChemExpress	HY-13322
Pravastatin	MedChemExpress	HY-B0165
Quercetin	MedChemExpress	HY-18085
Quizartinib	MedChemExpress	HY-13001
Ricolinostat	MedChemExpress	HY-16026
Romidepsin	MedChemExpress	HY-15149
SB-1317	Selleck Chemicals LLC	S7002
Selinexor	MedChemExpress	HY-17536
Simvastatin	Merck Life Science	S6196
Sorafenib	MedChemExpress	HY-10201
Sunitinib	MedChemExpress	HY-10255A
Temozolomide	MedChemExpress	HY-17364
Thalidomide	MedChemExpress	HY-14658

Thymoquinone	Merck Life Science	274666
Tipifarnib	MedChemExpress	HY-10502
Troxacitabine	MedChemExpress	HY-13770
Valproic acid	MedChemExpress	HY-10585
Venetoclax	MedChemExpress	HY-15531
Vincristine	Selleck Chemicals LLC	S9555
Volasertib	Selleck Chemicals LLC	S2235
Voreloxin	MedChemExpress	HY-10534
Vorinostat	MedChemExpress	HY-10221
Zosuquidar	MedChemExpress	HY-15255
**Antibodies**		
Phospho-Histone H2A.X (Ser139) Monoclonal Antibody (3F2)	Thermo Fisher Scientific	Cat# MA1-2022
Bcl-2 Monoclonal Antibody	Thermo Fisher Scientific	Cat# MA5-11757
Bcl-XL	Thermo Fisher Scientific	Cat# 66020-1-IG
Alexa Fluor™ 488	Thermo Fisher Scientific	Cat# A-11001
p21 Waf1/Cip1 (12D1) Rabbit mAb	Cell Signaling	2947T
Anti-KRAS Antibody	Merck	WH0003845M1
ACSM2A Polyclonal antibody	Proteintech	22862-1-AP
GSDMC Polyclonal antibody	Proteintech	30469-1-AP
PDE1A Polyclonal antibody	Proteintech	12442-2-AP
PSMA8 Monoclonal antibody	Proteintech	68123-1-Ig
Lamin B1 (D9V6H) Rabbit mAb	Cell Signaling	13435T
HRP-conjugated anti-rabbit	Jackson ImmunoResearch	711-035-152
HRP-conjugated anti-mouse	Jackson ImmunoResearch	715-035-151
**In vivo experimental models**		
FVB/NHanHsd	Harlan	86206F
Brca1-/-;p53-/- FVB mouse mammary tumors	The Netherlands Cancer Institute	MDZ1782
Navitoclax	TargetMol	T2101
DOXIL	Janssen Pharmaceuticals	132362608839
**Commercial assays, kits, dyes**		
Senescence β-Galactosidase Staining Kit	Cell Signaling	#9860
PrestoBlue® assay	Thermo Fisher Scientific	Cat# A13262
Nucleolus Bright Red	Dojindo EU	N512
SPiDER-βGal	Dojindo EU	SG03
MitoBright Red	Dojindo EU	MT07
LysoTracker Red	Thermo Fisher Scientific	Cat# L7528
Crystal Violet	Merck	C3886
Annexin V, FITC Apoptosis Detection Kit	Dojindo EU	AD10-10
DAPI	Dojindo EU	D523
Direct-zol® MiniPrep kit	Zymo Research	R2052
RNA Pico Sensitivity Assay Reagents	Perkin Elmer	CLS960012
Quant-iT 1x dsDNA HS Assay kit	Thermo Fisher Scientific	Cat# Q33232
Asteria™ Single-cell RNA-seq Benchtop Kit	SCIPIO BIOSCIENCE	Cat# 001-1000
PolyA beads 2.0 kit	Perkin Elmer	NOVA-512991
NextFlex Rapid Directional RNA-seq Kit 2.0	Perkin Elmer	NOVA-5198-01

DNA NGS 3k Assay kit	Perkin Elmer	CLS960013
**Other**		
PVDF membrane	Bio-Rad	Cat# 1620177
Mini-PROTEAN TGX Gels	Bio-Rad	Cat# 4561093
TRIzol™ Reagent	Thermo Fisher Scientific	AM9738
**Softwares**		
GraphPad Prism version 8.0.1	GraphPad Software	RRID:SCR_002798
Image Lab	Bio-Rad	RRID:SCR_014210
Fastq Toolkit (v2.2.5)	Illumina	
Rstudio		RRID:SCR_000432
edgeR (v4.0.16)		RRID:SCR_012802
limma (v3.58.1)		RRID:SCR_010943
fgsea (v1.28.0)		RRID:SCR_020938
STAR (v2.7.10)		RRID:SCR_004463
Subread (v2.0.3)		RRID:SCR_009803
Cytonaut platform (v2.1.0)	SCIPIO BIOSCIENCE	
Seurat package (v5.1.0)		RRID:SCR_016341
DoubletFinder (v2.0.4)		RRID:SCR_018771
clusterProfiler package (v4.6.2)		RRID:SCR_016884
Monocle3 (v1.3.1)		RRID:SCR_018685
CellChat (v1.6.1)		RRID:SCR_021946
Fragpipe v22.0		RRID:SCR_022864
Perseus 1.6.15		RRID:SCR_015753
Instantclue v0.12.2		RRID:SCR_024673
XcaliburTM 4.6	Thermo Fisher Scientific	RRID:SCR_014593
**Equipments**		
EnSpire Microplate Reader	Perkin Elmer	
Zeiss LSM-710	Zeiss	
Chemidoc MP	Bio-Rad	
Qubit 3.0 Fluorometer	Thermo Fisher Scientific	
DNA 5K/RNA/CZE Chip	Perkin Elmer	
Fluostar Omega	BMG Labtech	
NovaSeq 6000	Illumina	
ACQUITY UPLC M-Class LC system	Waters	
Orbitrap Exploris 240 mass spectrometer	Thermo Fisher Scientific	
JuLI™ Stage	NanoEntek	
