# Supplementary figures and images for: Therapy-induced senescence is a transient drug resistance mechanism in breast cancer
Source: Mol Cancer. 2025 May 1;24:128. doi: 10.1186/s12943-025-02310-0 (PMC12044945; doi:10.1186/s12943-025-02310-0)

MCF7

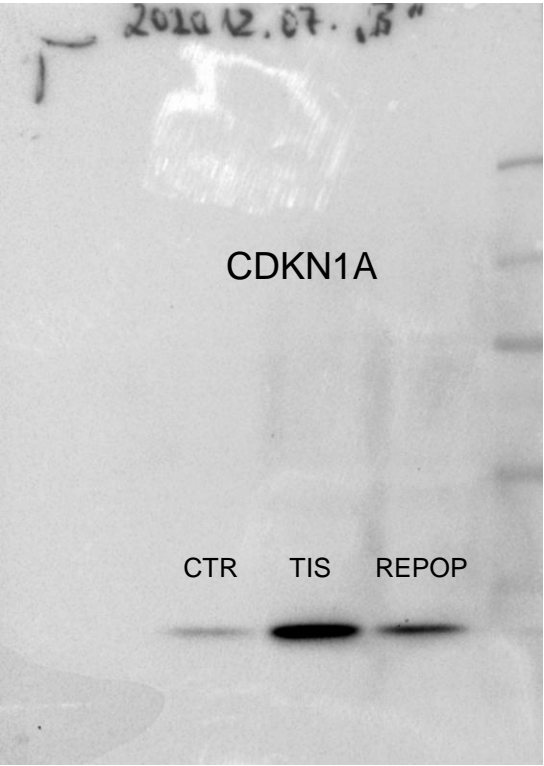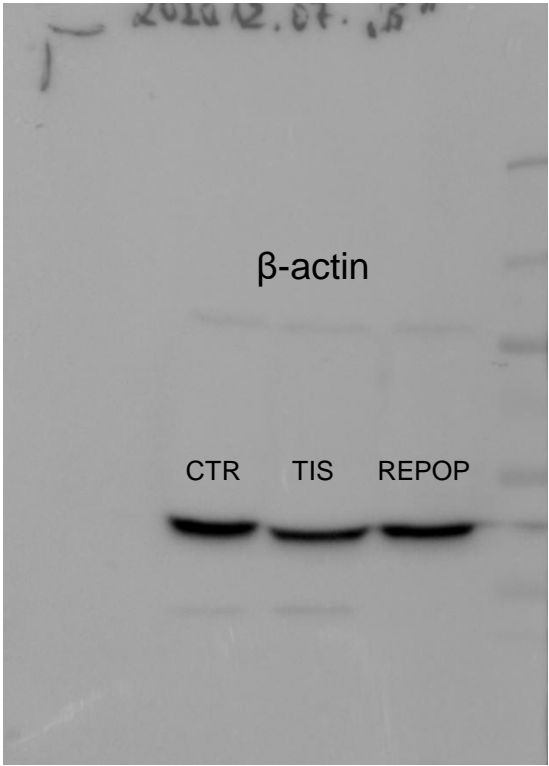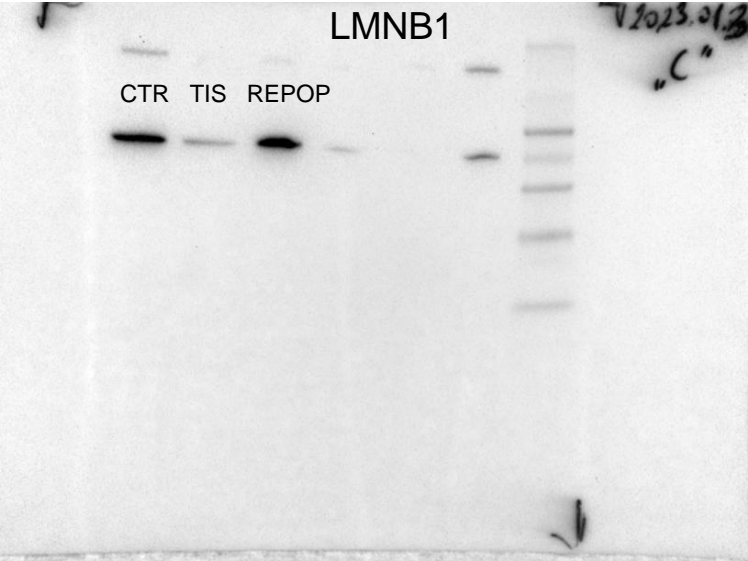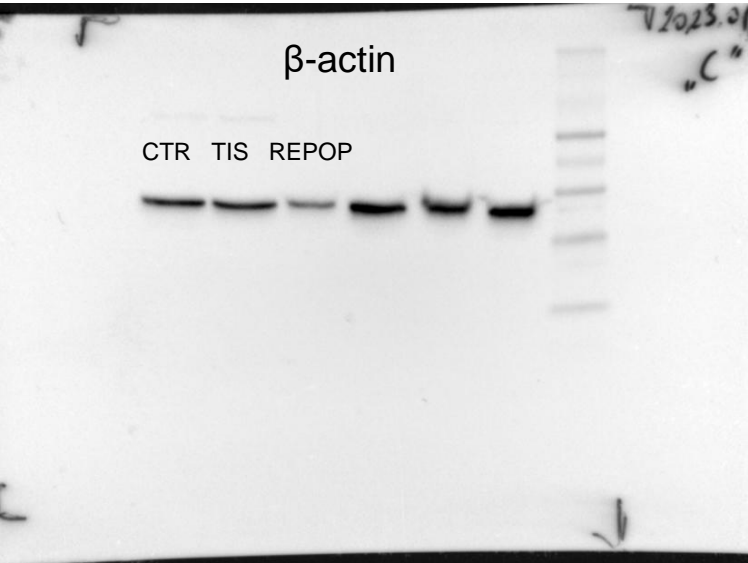

T47D

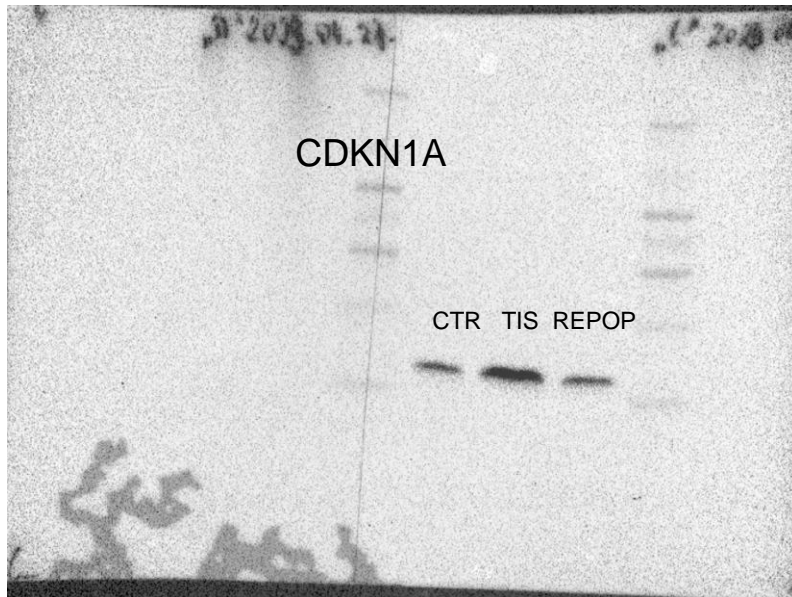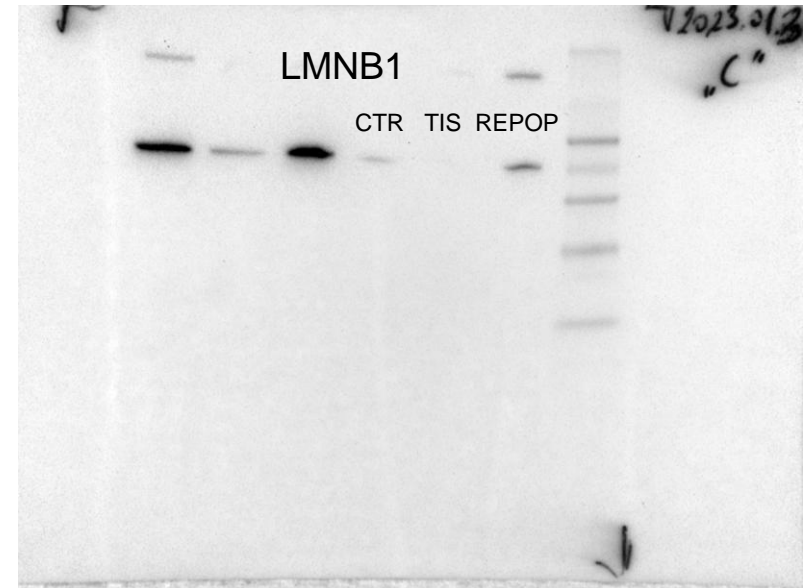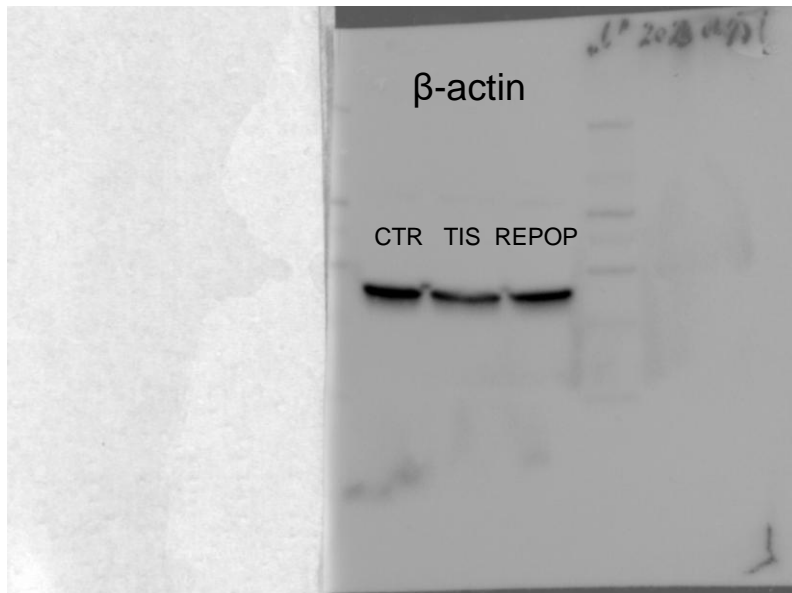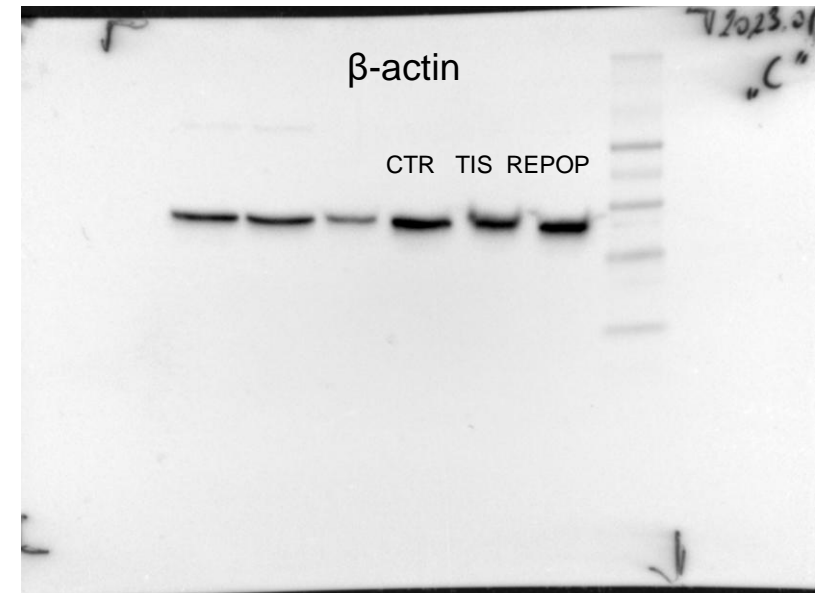

# MDA-MB-231

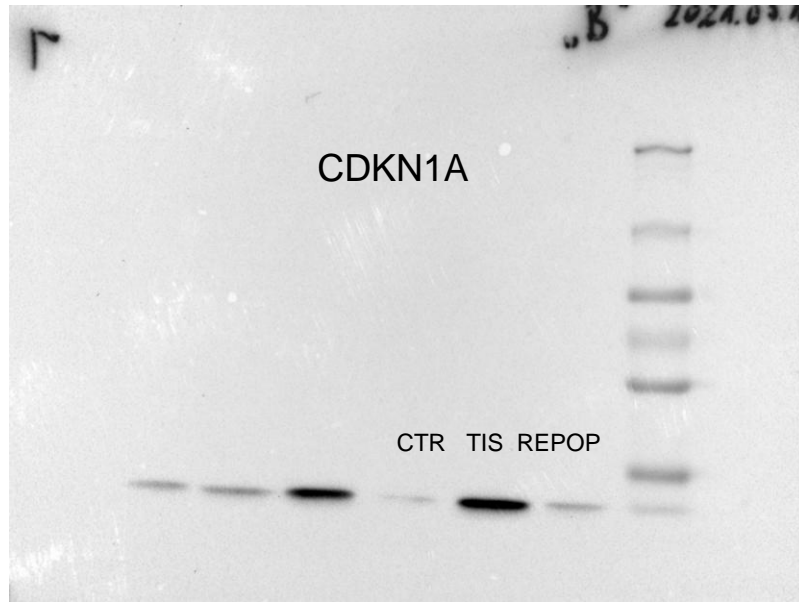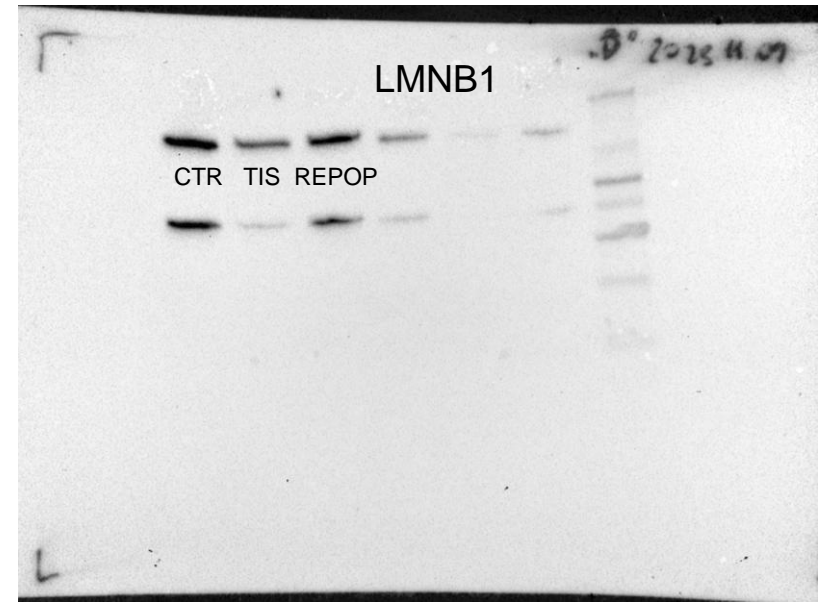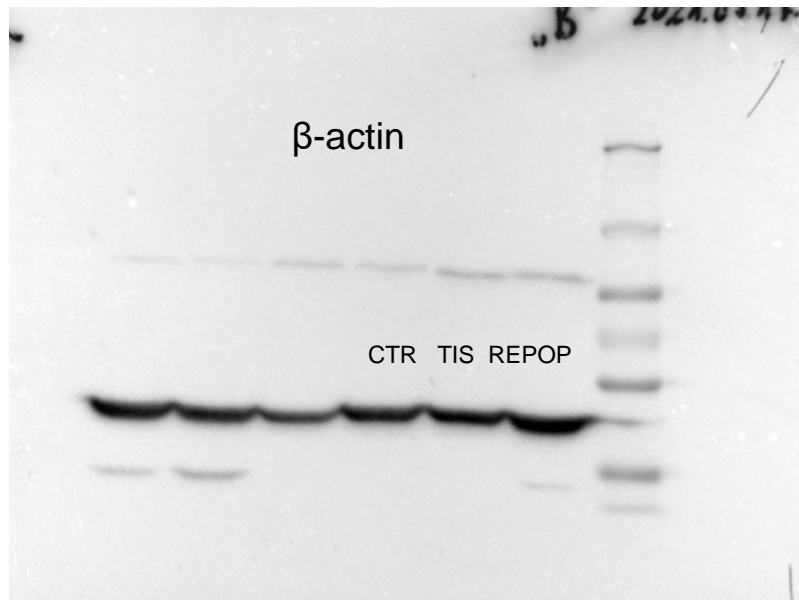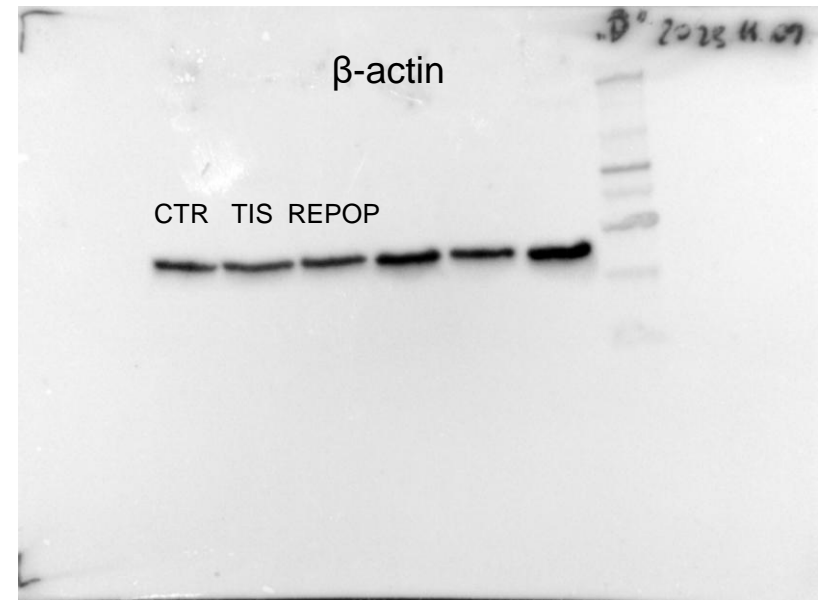

# Hs578T

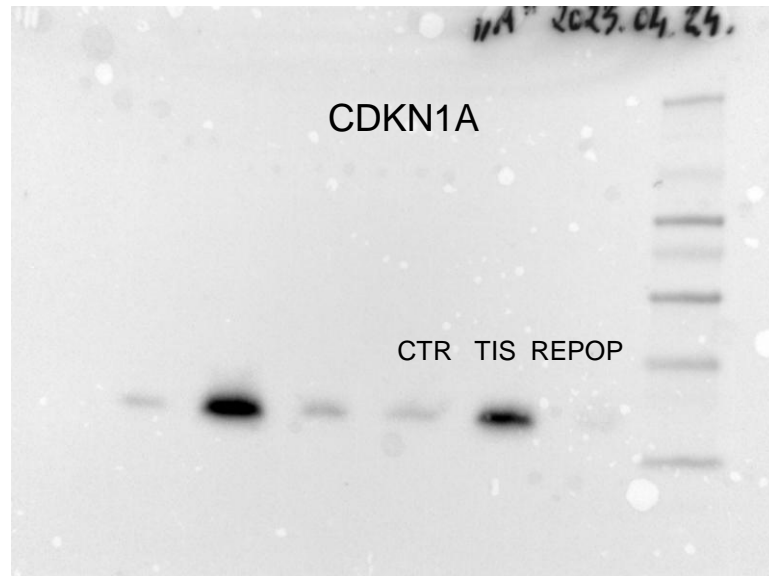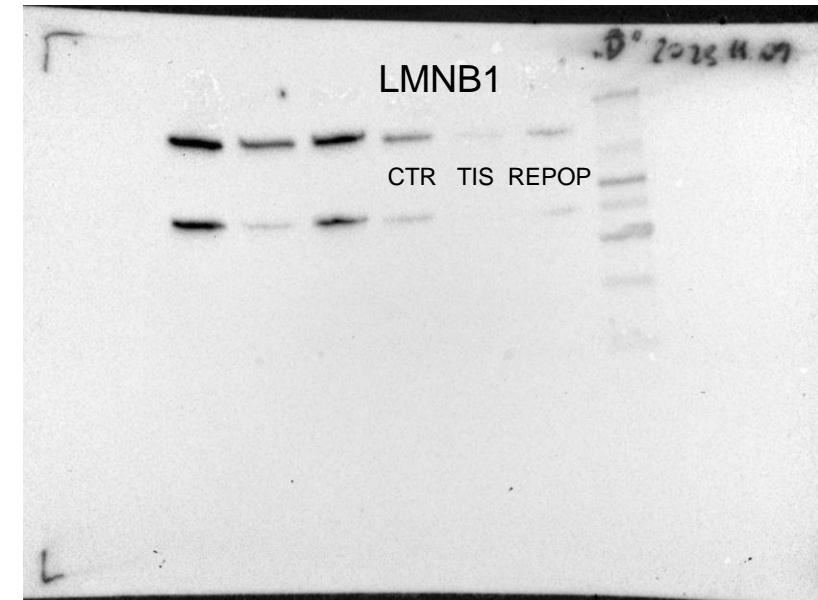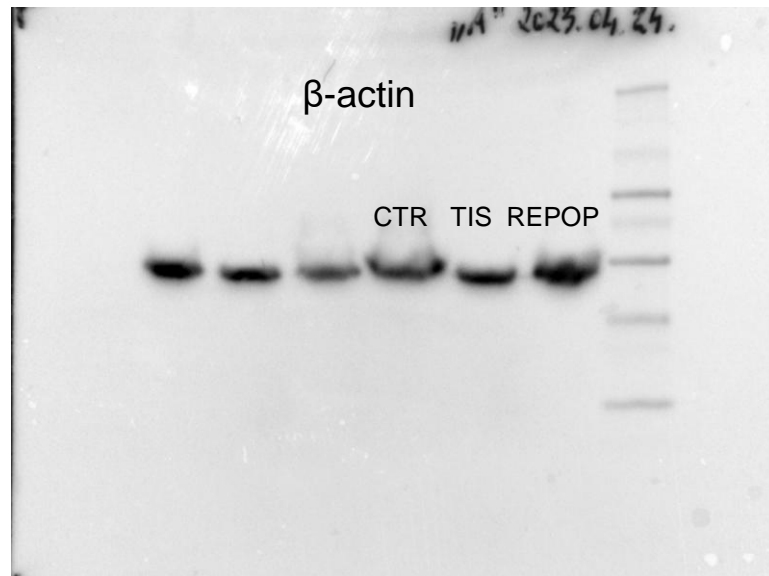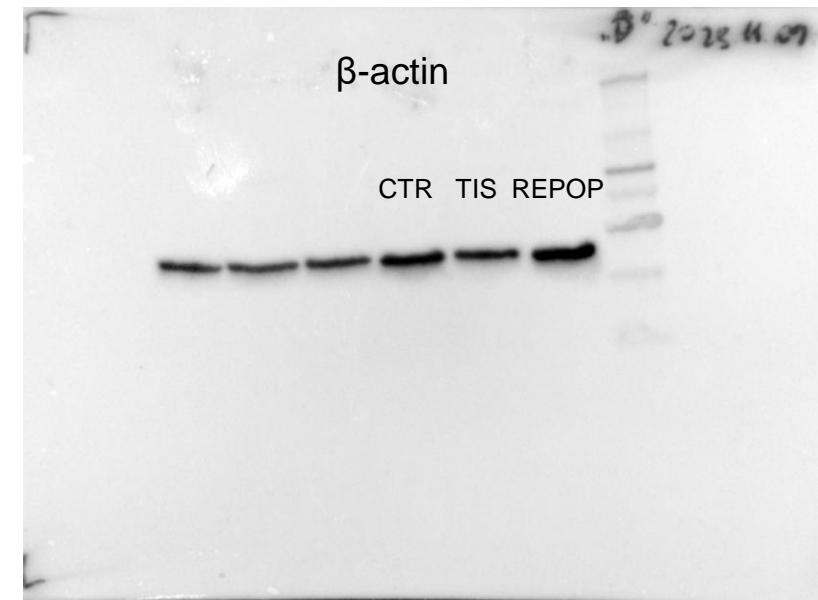

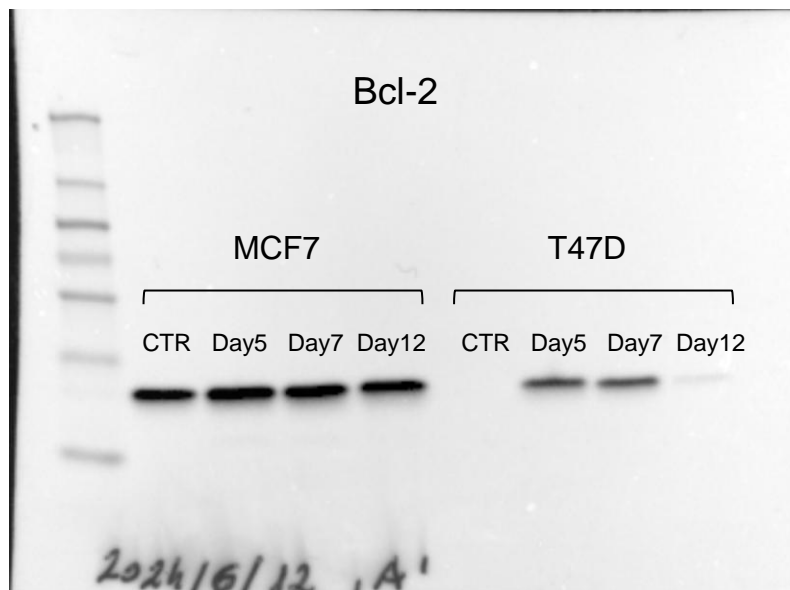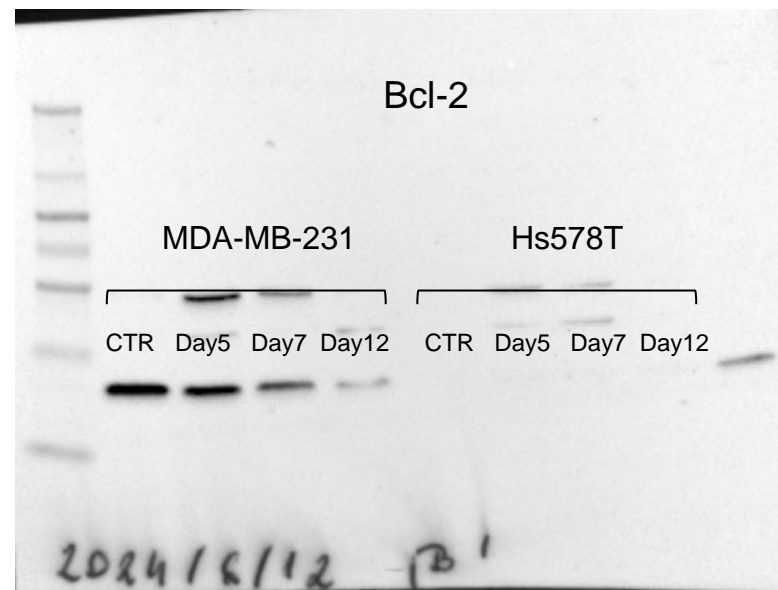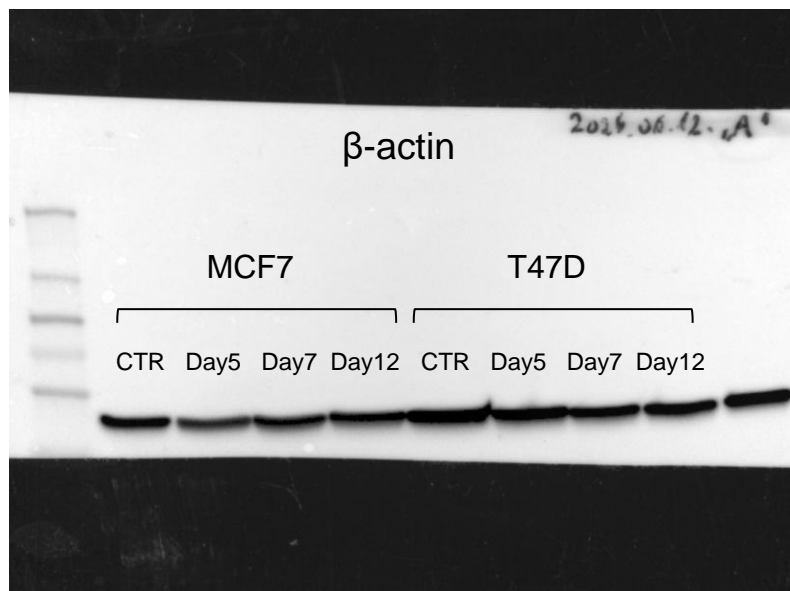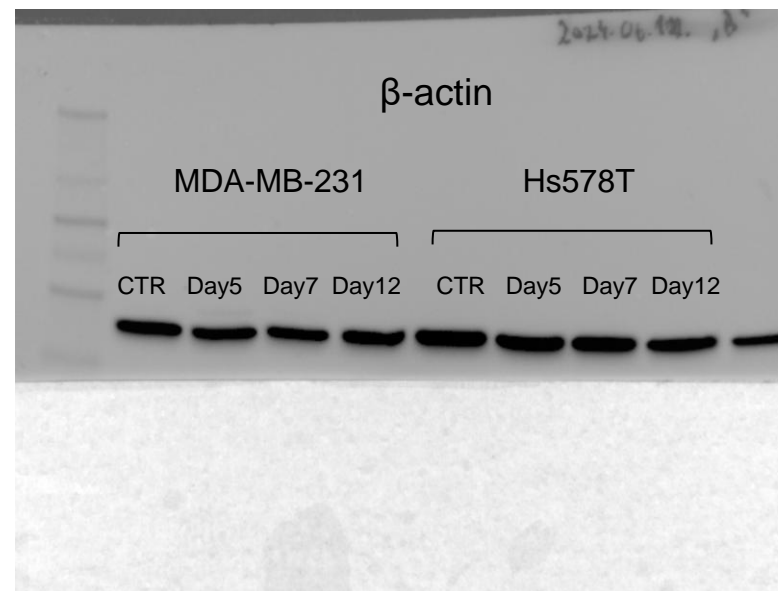

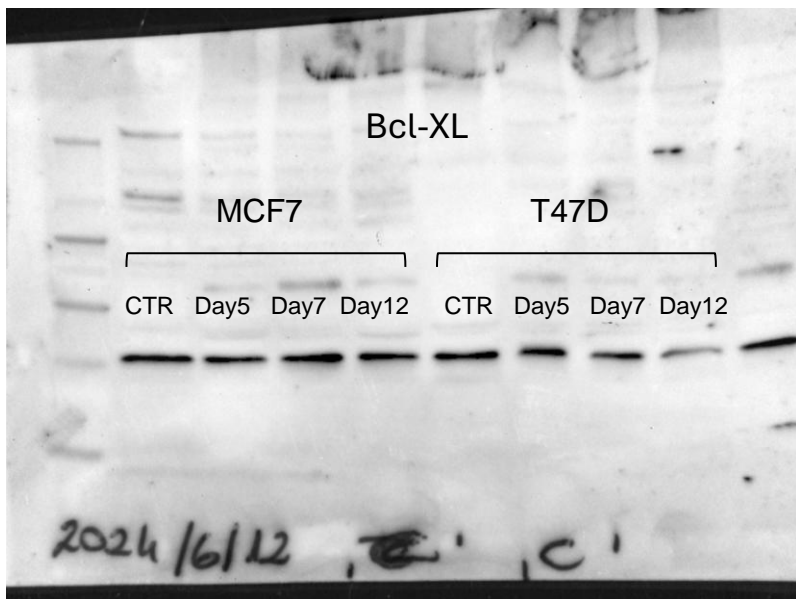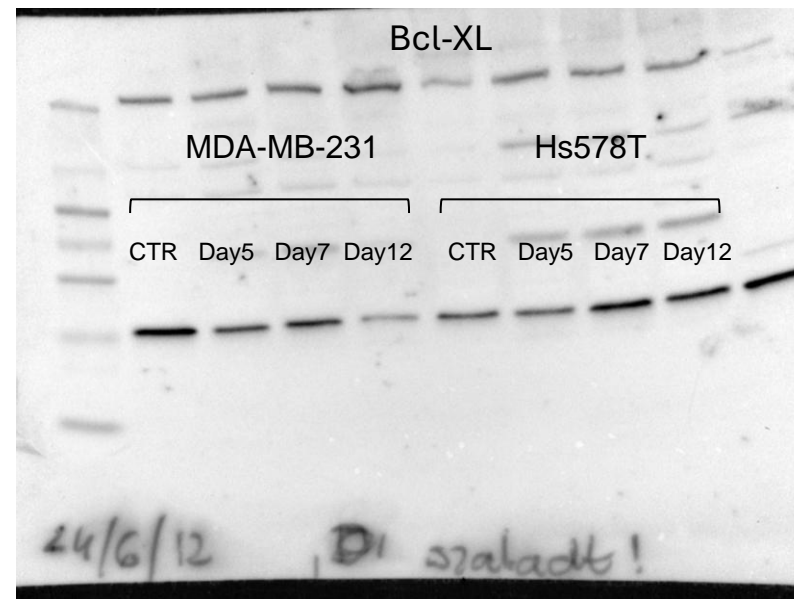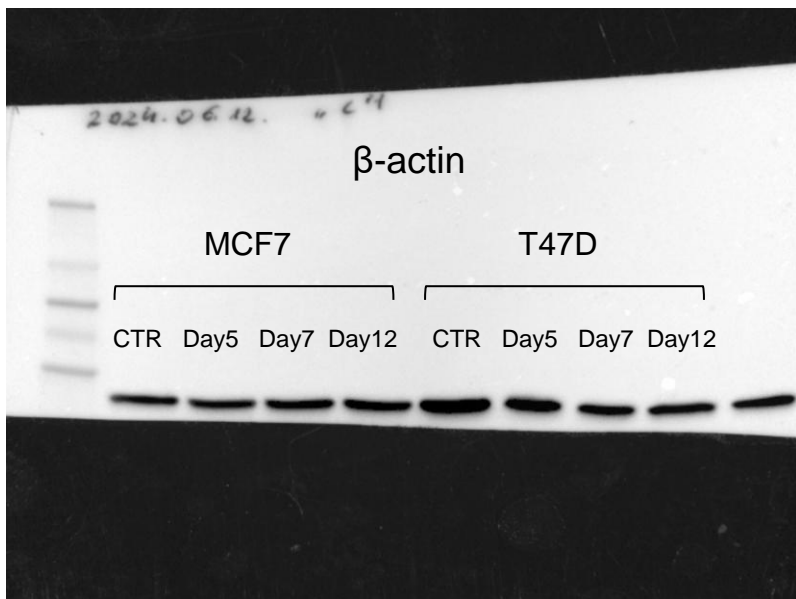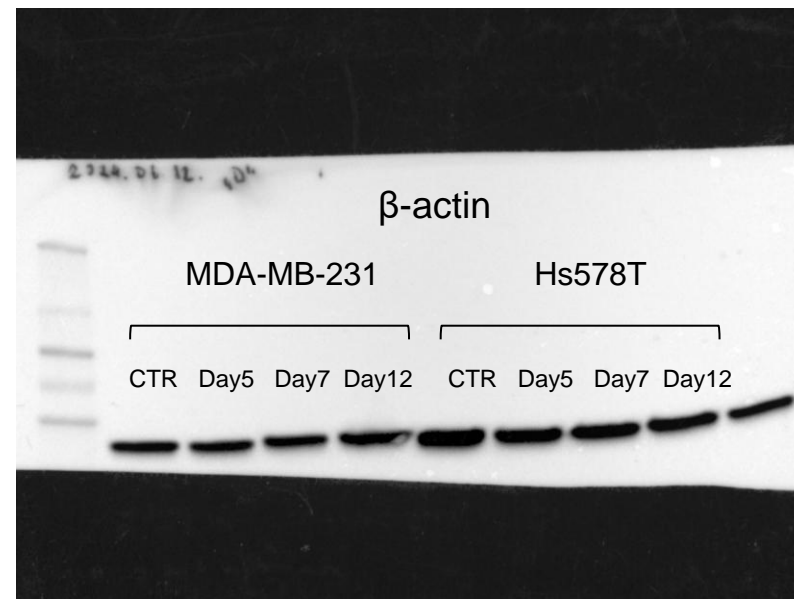

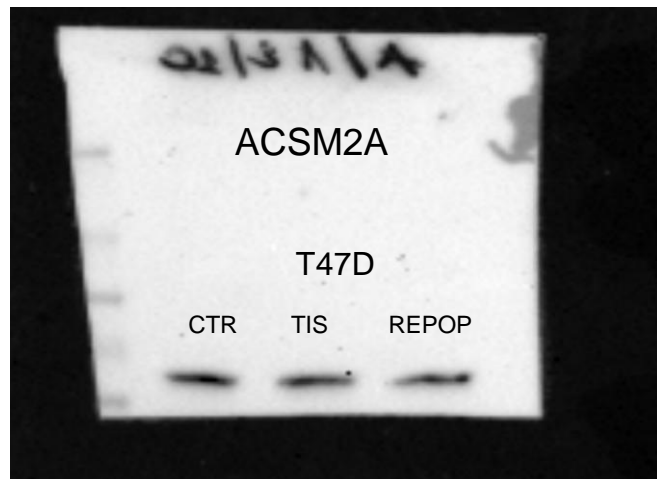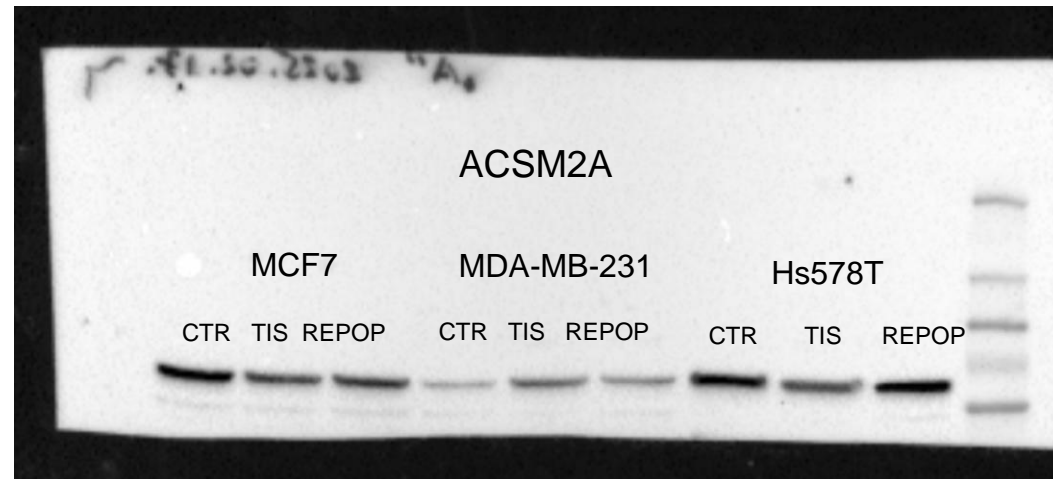

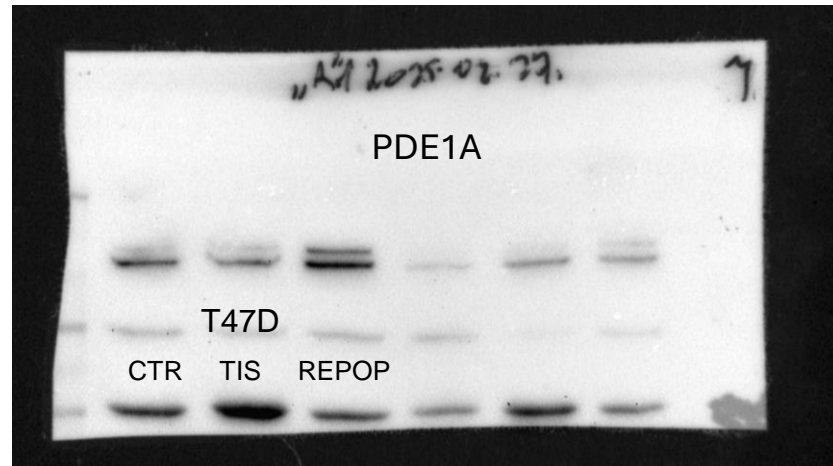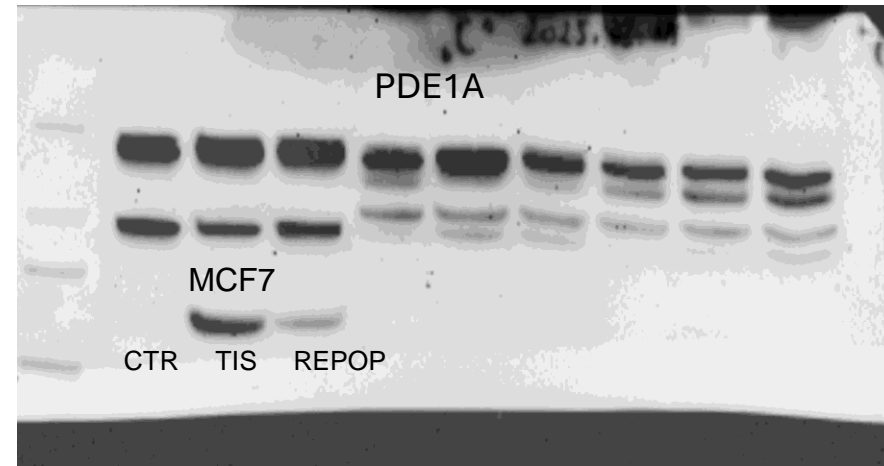

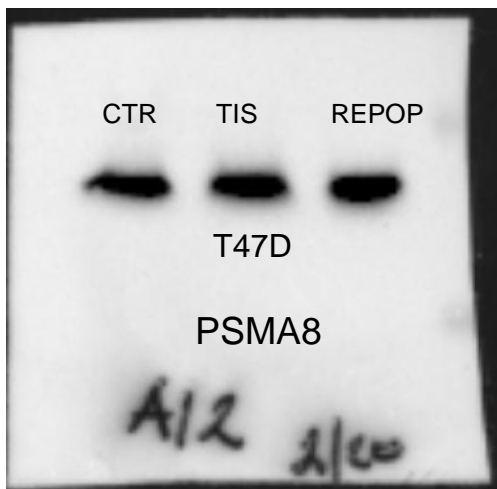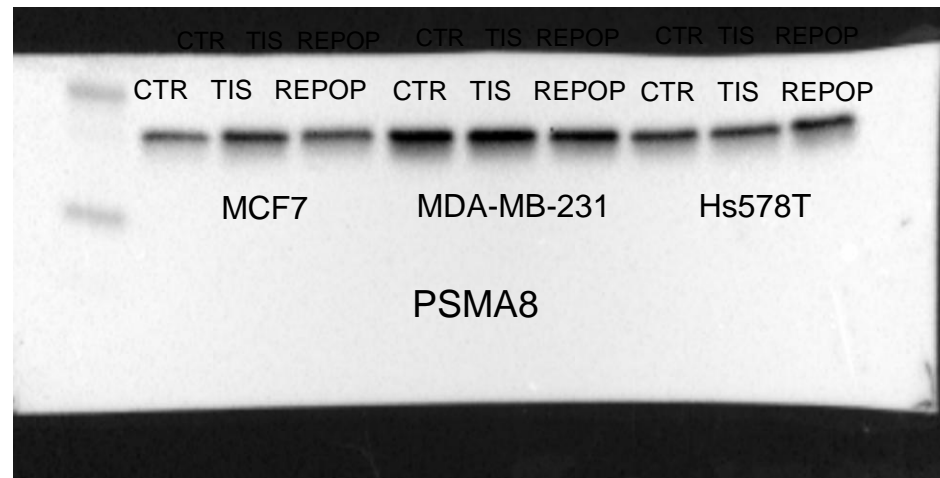

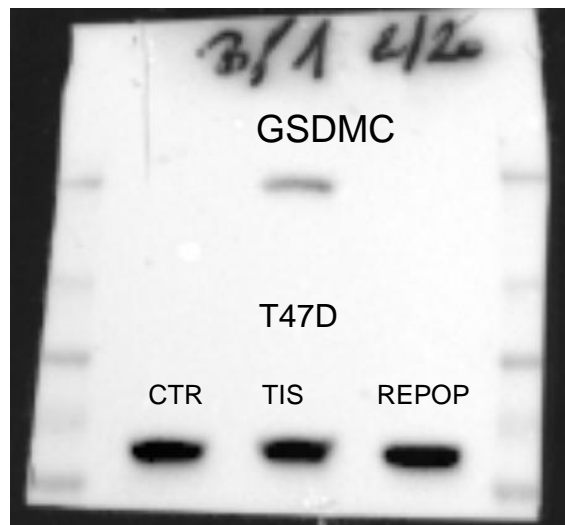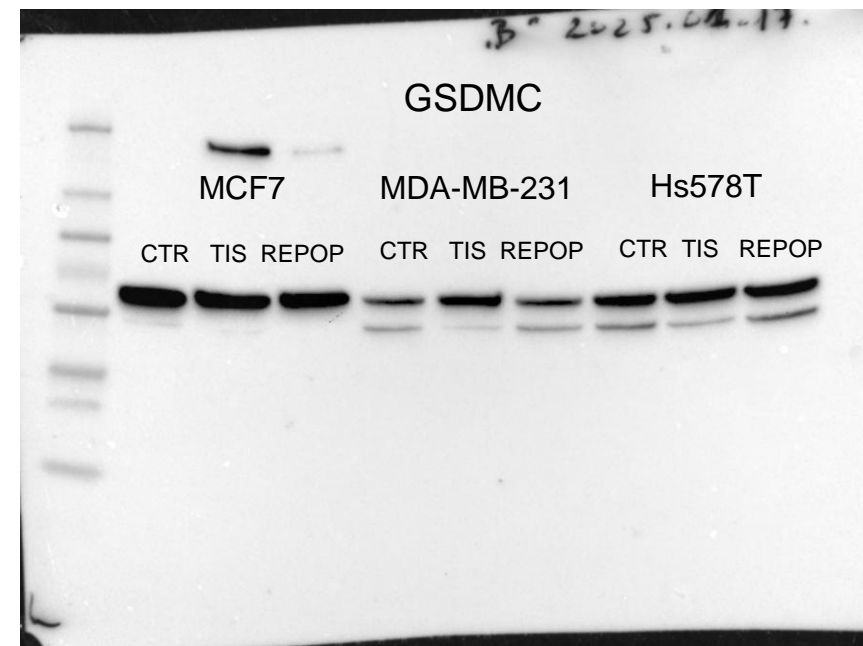

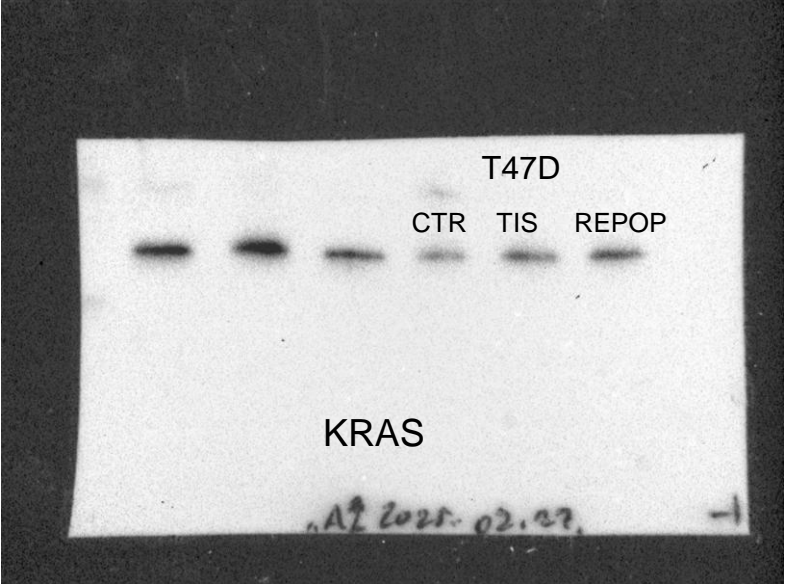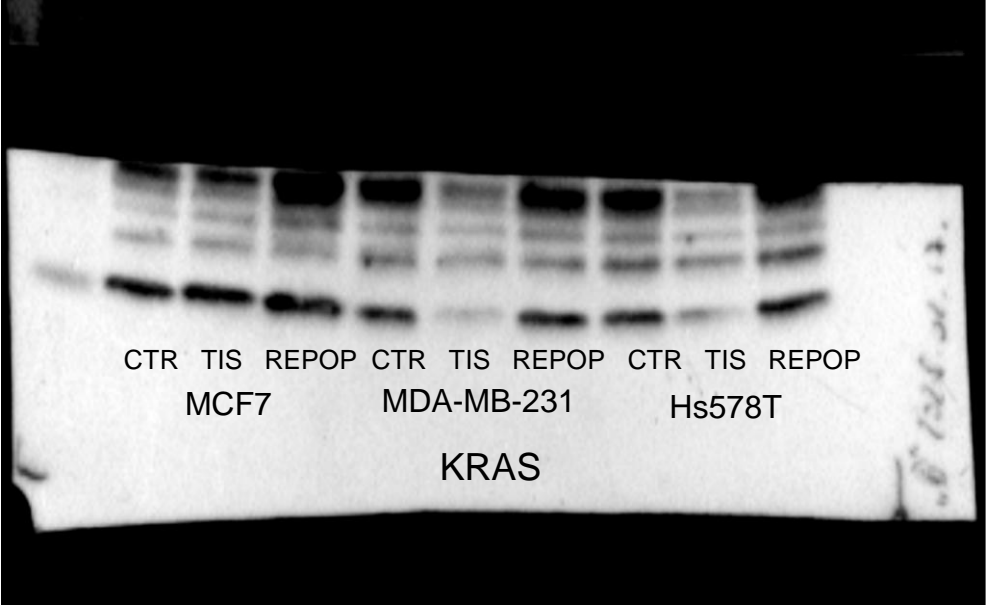

$\beta$ -actin

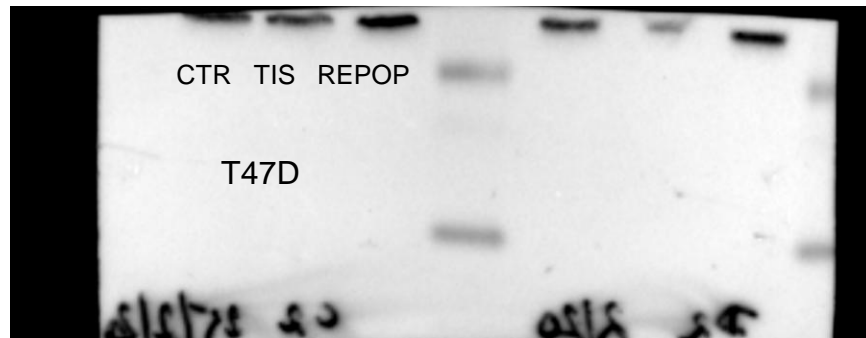

$\beta$ -actin

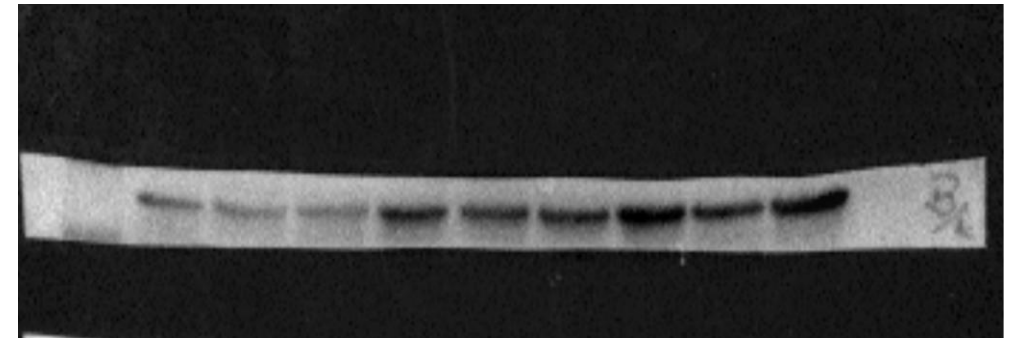

|      |     |       |            |     |       |        |     |       |
|------|-----|-------|------------|-----|-------|--------|-----|-------|
| CTR  | TIS | REPOP | CTR        | TIS | REPOP | CTR    | TIS | REPOP |
| MCF7 |     |       | MDA-MB-231 |     |       | Hs578T |     |       |

Supplement: Supplementary file 2 — Supplementary Material 2 [file 12943_2025_2310_MOESM2_ESM.pdf]
